# Six new species of *Pristimantis* (Anura: Strabomantidae) from Llanganates National Park and Sangay National Park in Amazonian cloud forests of Ecuador

**DOI:** 10.7717/peerj.13761

**Published:** 2022-10-17

**Authors:** Jhael A. Ortega, Jorge Brito, Santiago R. Ron

**Affiliations:** 1 Museo de Zoología, Escuela de Biología, Facultad de Ciencias Exactas y Naturales, Pontificia Universidad Católica del Ecuador, Quito, Pichincha, Ecuador; 2 Instituto Nacional de Biodiversidad (INABIO), Quito, Pichincha, Ecuador

**Keywords:** Andes, Amazon basin, Llanganates, New species, Osteology, Systematics, Taxonomy, Pristimantis, Strabomantidae, Sangay

## Abstract

We describe six new species of rainfrogs of the genus *Pristimantis* (Strabomantidae) from Amazonian cloud forests in Ecuador. We also present a phylogeny showing the relationships of the new species. The phylogeny is based on mitochondrial genes 16S rRNA (16S), 12 rRNA (12S), NADH-ubiquinone oxidoreductase chain 1 (ND1) and the nuclear gene recombination-activating 1 (RAG1). We also describe the osteology of two of the new species using high-resolution x-ray computed tomography. The new species belong to two clades. The first clade is sister to the subgenus *Huicundomantis* and includes *P. tamia* sp. nov., *P. miktos*, and *P. mallii*. *Pristimantis tamia* sp. nov. is morphologically similar to *P. miktos, P. mallii, P. martiae*, and *P. incomptus*, but differs from them by lacking vocal slits and tympanic membrane and by having light greenish blue iris. Based in our results we expand the subgenus *Huicundomantis* to include the *P. miktos* species group. The second clade is remarkable by being highly divergent and consisting exclusively of new species: *P. anaiae* sp. nov., *P. glendae* sp. nov., *P. kunam* sp. nov., *P. resistencia* sp. nov., and *P. venegasi* sp. nov. The new species resemble *P. roni*, *P. yanezi*, *P. llanganati*, *P. katoptroides*, *P. verecundus*, and *P. mutabilis* but can be distinguished from them by lacking vocal slits and tympanic membrane and by having large dark round areas with thin clear borders in the sacral region. All six new species occur in the eastern slopes of the Ecuadorian Andes and are known from a single locality in Llanganates or Sangay National Park. We recommend assigning all of them to the Data Deficient (DD) Red List category. Based in our high-resolution x-ray tomographies, we report the presence of structures that appear to be intercalary elements. This would be the first report of such structures in Terrarana.

## Introduction

With more than 569 species distributed from eastern Honduras and Panama through the Andes to Bolivia, north Argentina, and Brazil, *Pristimantis* is the most speciose genus among land-living vertebrates (Lynch & Duellman, 1997; Frost, 2022). Being direct developers (terrestrial eggs and no tadpole), they do not depend on water bodies for their reproduction (Gomez-Mestre, Pyron & Wiens, 2012). It has been hypothesized that direct development might be one of the reasons explaining their great diversity, especially in the Andes, the region with the highest number of species (Pinto-Sánchez et al., 2012); however, that hypothesis has not been rigorously tested.

The use of genetic data has become crucial to understand species limits, especially in morphologically cryptic groups (Morard et al., 2016). Within *Pristimantis*, cryptic diversity appears to be pervasive as shown by recent molecular phylogenetic studies on which the number of undescribed cryptic species equals or exceeds the number of described species (*e.g*., Elmer & Cannatella, 2008; Padial & De la Riva, 2009; Hutter & Guayasamin, 2015; Ortega-Andrade et al., 2015; Páez & Ron, 2019; Zumel, Buckley & Ron, 2021).

In the last 5 years, the inclusion of molecular information in systematic reviews has allowed the description of 50 species (47 endemic) of *Pristimantis* from Ecuador (Ron, Merino-Viteri & Ortiz, 2021). Of them, 16 were described in 2019 (*e.g*., Páez & Ron, 2019; Urgiles et al., 2019; Reyes-Puig et al., 2019; Yánez-Muñoz et al., 2019). This suggests that species richness of Andean *Pristimantis* is significantly underestimated, and the number of species descriptions will continue to increase, especially in the highlands (*e.g*., Elmer, Davila & Lougheed, 2007; Ortega-Andrade et al., 2015; Navarrete, Venegas & Ron, 2016; Rivera-Correa & Daza, 2016; Szèkely et al., 2016; Reyes-Puig et al., 2019).

Most species richness of *Pristimantis* occurs in the Andes of Colombia and Ecuador. Within Ecuador, the Amazonian Montane Forests is the region with the highest species richness (Navarrete, Venegas & Ron, 2016) and where most species of *Pristimantis* have been discovered during the last years (Ron, Merino-Viteri & Ortiz, 2021). In that region, Llanganates National Park (LNP) and Sangay National Park (SNP) are largely unexplored protected areas comprising 7,375.6 km^2^ of montane humid forests and paramos. Amphibian inventories at Llanganates and Sangay have been scant due to the lack of access roads and hostile geographic and climatic conditions (Ministerio del Ambiente del Ecuador, 2017). Members of the Museo de Zoología, Pontificia Universidad Católica del Ecuador made several expeditions to both parks in 2009, 2015, 2017, and 2018 to carry out biodiversity inventories. In addition, JB made an expedition to Sangay National Park in 2013. So far, from those collections, six new species of *Pristimantis* have been discovered (*i.e.*, Navarrete, Venegas & Ron, 2016; Páez & Ron, 2019; Reyes-Puig et al., 2019; Carrión-Olmedo & Ron, 2021; Zumel, Buckley & Ron, 2021). In this publication, we describe six additional species and infer their evolutionary relationships.

## Materials and Methods

The electronic version of this article in Portable Document Format (PDF) will represent a published work according to the International Commission on Zoological Nomenclature (ICZN), and hence the new names contained in the electronic version are effectively published under that Code from the electronic edition alone. This published work and the nomenclatural acts it contains have been registered in ZooBank, the online registration system for the ICZN. The ZooBank LSIDs (Life Science Identifiers) can be resolved, and the associated information viewed through any standard web browser by appending the LSID to the prefix http://zoobank.org/. The LSID for this publication is: urn:lsid:zoobank.org:pub:AFBD6AC9-D3E2-4112-AC3F-A2156268ECB3. The online version of this work is archived and available from the following digital repositories: PeerJ, PubMed Central SCIE and CLOCKSS.

### Ethics statement

Voucher specimens and tissue samples were obtained following ethical and technical protocols (Esselstyn et al., 2008). We conducted this research under collection permits, N° 005-12-IC-FAU-DNB/MA, N° 008-09 IC-FAU-DNB/MA and MAE-DNB-ARRGG-CM-2014-0002, issued by the Ministerio de Ambiente del Ecuador to the Pontificia Universidad Católica del Ecuador.

### Sampling of species and populations

**Figure 1:**
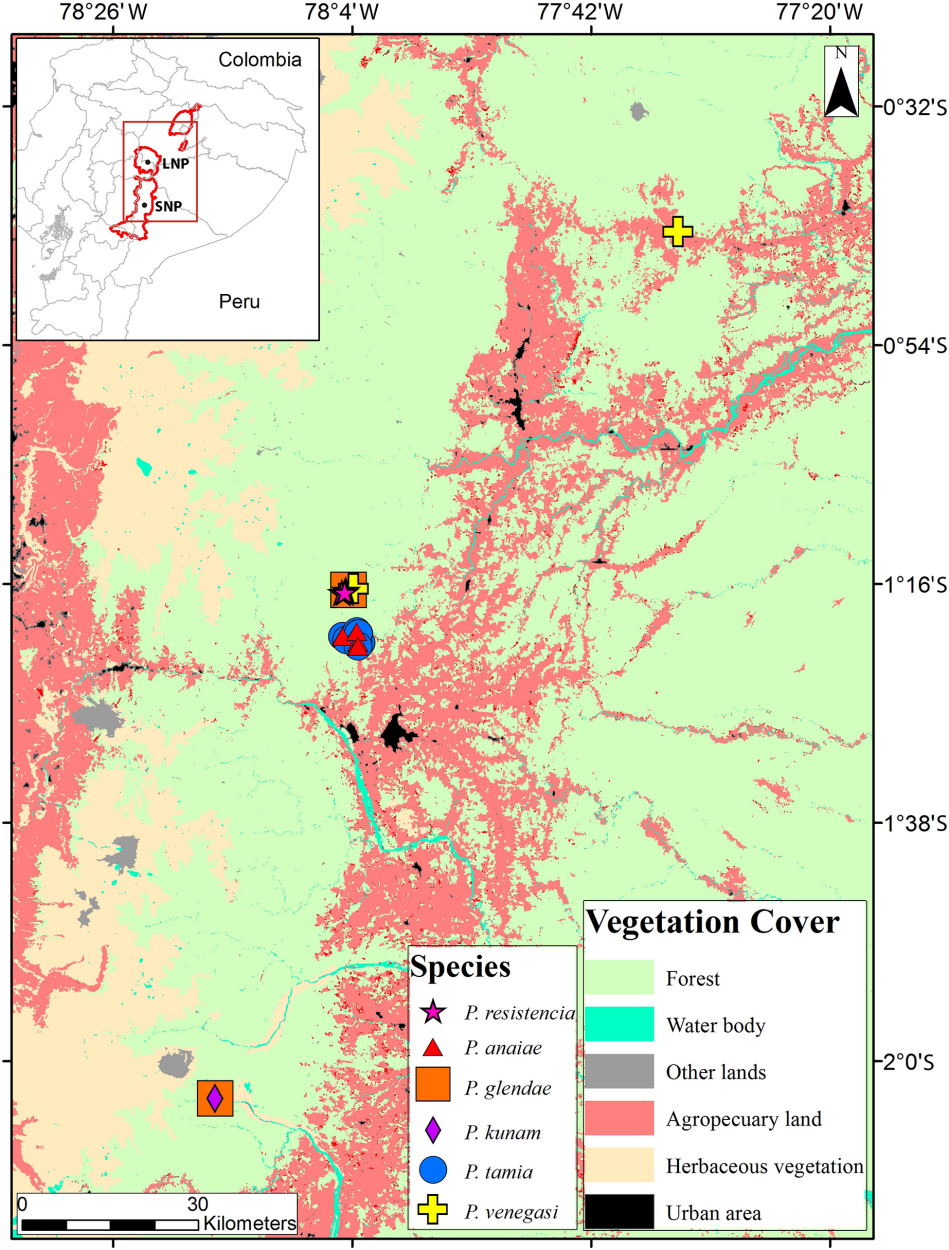
Collection localities of *Pristimantis* new species. The map shows the use of land.

Our analysis focused on *Pristimantis* specimens collected during field trips to Llanganates National Park and Sangay National Park (Fig. 1). The study group was expanded to include species shown to be closely related to the new species based on an unpublished phylogeny of *Pristimantis* obtained by Santiago R. Ron as part of a large-scale review of Ecuadorian *Pristimantis*, which includes sequences previously published by Hedges, Duellman & Heinicke (2008), Padial, Grant & Frost (2014), Páez & Ron (2019), Reyes-Puig et al. (2019).

### DNA extraction, amplification, and sequencing

DNA was extracted from muscle or liver tissue preserved in 95% ethanol. We performed a polymerase chain reaction (PCR) to amplify DNA fragments for mitochondrial genes 12S rRNA (12S), 16S rRNA (16S), NADH-ubiquinone oxidoreductase chain 1 (ND1) and nuclear gene recombination-activating 1 (RAG1) and tRNAs leucine, isoleucine and glutamine. PCR amplification was carried out following standardized protocols (*e.g*., Hedges, Duellman & Heinicke, 2008) using the following primers: MVZ59 and 12Sh (Goebel, Donnelly & Atz, 1999) for 12S rRNA; 16H36E, 16L19, 16H47 and 16L34 (Heinicke, Duellman & Hedges, 2007) for 16S rRNA; 16Sfrog, tMet-frog (Wiens et al., 2005), WL379 and WL384 (Moen & Wiens, 2009) for ND1; and R182 and R270 (Hedges, Duellman & Heinicke, 2008) for RAG1. Sequencing was performed by the Macrogen Sequencing Team (Macrogen Inc., Seoul, Korea).

### Phylogenetic analyses and species delimitation

We generated 143 new sequences (58 of 16S, 17 of 12S, 19 of ND1 and 49 of RAG1) of 59 individuals which were assembled and edited with Geneious 7.1.7 (GeneMatters Corp, Minneapolis, MN, USA). The sequences generated in this study were deposited in GenBank (accession numbers shown in Table 1). We barcoded 33 specimens of *P. tamia* sp. nov. to corroborate their identifications. Those sequences were not included in the phylogeny but are uploaded in GenBank (access numbers shown in Table 1). After assemblage, the new sequences were combined with sequences from GenBank. We blasted our 12S, 16S, ND1 and RAG1 sequences with the GenBank database (*blastn* procedure) to find similar sequences and add them to the matrix. Additionally, we included sequences of *P. bicantus* Guayasamin & Funk, 2009, *P. nelsongalloi* Valencia et al., 2019 and *P. prolatus* (Lynch & Duellman, 1980) to determine their phylogenetic position. For the outgroup (Table S1) we included samples of subgenus *Hypodictyon* (based on Hedges, Duellman & Heinicke, 2008 and Padial, Grant & Frost, 2014). GenBank sequences were originally published by Darst & Cannatella (2004), Duellman & Hedges (2005), Lehr, Fritzsch & Mueller (2005), Wiens et al. (2005), Elmer, Davila & Lougheed (2007), Heinicke, Duellman & Hedges (2007), Hedges, Duellman & Heinicke (2008), Padial et al. (2009), Crawford, Lips & Bermingham (2010), Fouquet et al. (2012), Garcia-R et al. (2012), Kok et al. (2012), Pinto-Sánchez et al. (2012), Arteaga, Yánez-Muñoz & Guayasamin (2013), Ortega-Andrade & Venegas (2014), Ortega-Andrade et al. (2017), Páez & Ron (2019) and Reyes-Puig et al. (2019).

**Table 1:** GenBank accession numbers for newly generated DNA sequences used in the phylogenetic analyses.

Species	Voucher	RAG1	ND1	16S	12S
*Lynchius oblitus*	QCAZ 61035	MZ332965	MZ189449	MZ241534	MZ330741
*Pristimantis anaiae*	QCAZ 59562	MZ332952	–	MZ241520	–
*Pristimantis anaiae*	QCAZ 59566	MZ332953	–	MZ241521	–
*Pristimantis anaiae*	QCAZ 59627	MZ332957	MZ189441	MZ241525	–
*Pristimantis anaiae*	QCAZ 59640	MZ332959	MZ189443	MZ241527	–
*Pristimantis anaiae*	QCAZ 59693	MZ332966	MZ189448	MZ241532	MZ330740
*Pristimantis anaiae*	QCAZ 59720	MZ332964	–	MZ241533	–
*Pristimantis bicantus*	QCAZ 16186	MZ332920	MZ189433	MZ241483	–
*Pristimantis bicantus*	QCAZ 31985	MZ332924	–	MZ241487	–
*Pristimantis bicantus*	QCAZ 31986	MZ332925	–	MZ241488	–
*Pristimantis bicantus*	QCAZ 31988	MZ332926	–	MZ241489	–
*Pristimantis bicantus*	QCAZ 46184	MZ332940	MZ189434	MZ241504	–
*Pristimantis bicantus*	QCAZ 51553	MZ332942	MZ189436	MZ241506	–
*Pristimantis bicantus*	QCAZ 52489	MZ332943	MZ189437	MZ241507	–
*Pristimantis bicantus*	QCAZ 56440	MZ332948	–	MZ241513	–
*Pristimantis bicantus*	QCAZ 58597	–	–	–	MZ330732
*Pristimantis bicantus*	QCAZ 58972	MZ332951	–	MZ241516	–
*Pristimantis glendae*	QCAZ 45784	MZ332935	–	MZ241499	–
*Pristimantis glendae*	QCAZ 45793	MZ332936	–	MZ241500	–
*Pristimantis glendae*	QCAZ 45832	MZ332937	–	MZ241501	MZ330731
*Pristimantis glendae*	QCAZ 45953	MZ332939	–	MZ241503	–
*Pristimantis glendae*	QCAZ 56437	MZ332946	–	MZ241511	–
*Pristimantis kunam*	QCAZ 56438	MZ332947	–	MZ241512	–
*Pristimantis mallii*	QCAZ 45743	MZ332928	–	MZ241491	–
*Pristimantis mallii*	QCAZ 45744	–	–	MZ241492	MZ330729
*Pristimantis mallii*	QCAZ 45745	MZ332929	–	MZ241493	–
*Pristimantis mallii*	QCAZ 45748	MZ332930	–	MZ241494	–
*Pristimantis mallii*	QCAZ 45767	MZ332931	–	MZ241495	–
*Pristimantis mallii*	QCAZ 45770	MZ332932	–	MZ241496	MZ330730
*Pristimantis mallii*	QCAZ 45775	MZ332933	–	MZ241497	–
*Pristimantis miktos*	QCAZ 55445	–	MZ189440	MZ241510	–
*Pristimantis nelsongalloi*	QCAZ 31860	MZ332922	–	MZ241485	–
*Pristimantis nelsongalloi*	QCAZ 31861	MZ332923	–	MZ241486	–
*Pristimantis nelsongalloi*	QCAZ 59202	–	–	MZ241517	MZ330733
*Pristimantis nelsongalloi*	QCAZ 59211	–	–	MZ241518	–
*Pristimantis prolatus*	QCAZ 52515	MZ332945	MZ189439	MZ241509	–
*Pristimantis prolatus*	QCAZ 58963	MZ332950	–	MZ241515	–
*Pristimantis prolatus*	QCAZ 59480	–	–	MZ241519	–
*Pristimantis prolatus*	QCAZ 59586	MZ332955	–	MZ241523	–
*Pristimantis prolatus*	QCAZ 59603	MZ332956	–	MZ241524	–
*Pristimantis resistencia*	QCAZ 66467	–	–	OM729994	OM730031
*Pristimantis resistencia*	QCAZ 66519	–	–	OM729993	OM730030
*Pristimantis resistencia*	QCAZ 66523	–	–	OM729995	OM730032
*Pristimantis sacharuna*	QCAZ 52496	MZ332944	MZ189438	MZ241508	–
*Pristimantis* sp.	QCAZ 45720	MZ332927	–	MZ241490	–
*Pristimantis* sp.	QCAZ 45780	MZ332934	–	MZ241498	–
*Pristimantis* sp.	QCAZ 45945	MZ332938	–	MZ241502	–
*Pristimantis* sp.	QCAZ 46223	MZ332941	MZ189435	MZ241505	–
*Pristimantis* sp.	QCAZ 58855	MZ332949	–	MZ241514	–
*Pristimantis* sp.	QCAZ 69791	–	MZ189450	MZ241535	–
*Pristimantis* sp.	QCAZ 70020	–	MZ189451	MZ241536	–
***Pristimantis tamia***	**QCAZ 59439**	**-**	**-**	**OM729996**	**-**
***Pristimantis tamia***	**QCAZ 59445**	**-**	**-**	**OM729997**	**-**
***Pristimantis tamia***	**QCAZ 59564**	**-**	**-**	**OM729998**	**-**
***Pristimantis tamia***	**QCAZ 59565**	**-**	**-**	**OM729999**	**-**
*Pristimantis tamia*	QCAZ 59573	MZ332954	–	MZ241522	MZ330734
***Pristimantis tamia***	**QCAZ 59581**	**-**	**-**	**OM730000**	**-**
***Pristimantis tamia***	**QCAZ 59582**	**-**	**-**	**OM730001**	**-**
***Pristimantis tamia***	**QCAZ 59584**	**-**	**-**	**OM730002**	**-**
***Pristimantis tamia***	**QCAZ 59585**	**-**	**-**	**OM730003**	**-**
***Pristimantis tamia***	**QCAZ 59593**	**-**	**-**	**OM730004**	**-**
***Pristimantis tamia***	**QCAZ 59619**	**-**	**-**	**OM730005**	**-**
***Pristimantis tamia***	**QCAZ 59620**	**-**	**-**	**OM730006**	**-**
***Pristimantis tamia***	**QCAZ 59629**	**-**	**-**	**OM730007**	**-**
*Pristimantis tamia*	QCAZ 59630	MZ332958	MZ189442	MZ241526	MZ330735
***Pristimantis tamia***	**QCAZ 59635**	**-**	**-**	**OM730008**	**-**
***Pristimantis tamia***	**QCAZ 59636**	**-**	**-**	**OM730009**	**-**
***Pristimantis tamia***	**QCAZ 59639**	**-**	**-**	**OM730010**	**-**
***Pristimantis tamia***	**QCAZ 59642**	**-**	**-**	**OM730011**	**-**
*Pristimantis tamia*	QCAZ 59643	MZ332960	MZ189444	MZ241528	MZ330736
***Pristimantis tamia***	**QCAZ 59644**	**-**	**-**	**OM730012**	**-**
***Pristimantis tamia***	**QCAZ 59650**	**-**	**-**	**OM730013**	**-**
***Pristimantis tamia***	**QCAZ 59651**	**-**	**-**	**OM730014**	**-**
*Pristimantis tamia*	QCAZ 59653	MZ332961	MZ189445	MZ241529	MZ330737
***Pristimantis tamia***	**QCAZ 59656**	**-**	**-**	**OM730015**	**-**
***Pristimantis tamia***	**QCAZ 59660**	**-**	**-**	**OM730016**	**-**
***Pristimantis tamia***	**QCAZ 59664**	**-**	**-**	**OM730017**	**-**
***Pristimantis tamia***	**QCAZ 59666**	**-**	**-**	**OM730018**	**-**
*Pristimantis tamia*	QCAZ 59668	MZ332962	MZ189446	MZ241530	MZ330738
***Pristimantis tamia***	**QCAZ 59672**	**-**	**-**	**OM730019**	**-**
***Pristimantis tamia***	**QCAZ 59675**	**-**	**-**	**OM730020**	**-**
*Pristimantis tamia*	QCAZ 59680	MZ332963	MZ189447	MZ241531	MZ330739
***Pristimantis tamia***	**QCAZ 59696**	**-**	**-**	**OM730021**	**-**
***Pristimantis tamia***	**QCAZ 59701**	**-**	**-**	**OM730022**	**-**
***Pristimantis tamia***	**QCAZ 59702**	**-**	**-**	**OM730023**	**-**
***Pristimantis tamia***	**QCAZ 59704**	**-**	**-**	**OM730024**	**-**
***Pristimantis tamia***	**QCAZ 59705**	**-**	**-**	**OM730025**	**-**
***Pristimantis tamia***	**QCAZ 59710**	**-**	**-**	**OM730026**	**-**
***Pristimantis tamia***	**QCAZ 59713**	**-**	**-**	**OM730027**	**-**
***Pristimantis tamia***	**QCAZ 59719**	**-**	**-**	**OM730028**	**-**
*Pristimantis venegasi*	QCAZ 31130	MZ332921	–	MZ241484	–
*Pristimantis venegasi*	QCAZ 66440	OM752308	–	OM729992	OM730029

**Note:**

Individuals in bold were barcoded to confirm their identification but were not included in the phylogenetic analysis.

We imported the sequences in Mesquite version 2.75 (Maddison & Maddison, 2011). The sequences of each gene were independently aligned using the Muscle extension under default parameters (Edgar, 2004) in Mesquite. Each alignment was inspected visually for unambiguous alignment errors that were adjusted manually. The matrix was concatenated and exported using Mesquite. To ensure the replicability of our results, the aligned matrix is available on Zenodo (http://doi.org/10.5281/zenodo.7055289).

Since different evolutionary processes have molded each gene, we partitioned the matrix by gene and, for coding genes (ND1 and RAG), by codon position to find the best evolution model for each and then to find the best partition scheme. To perform these tasks, we used the command MFP + MERGE (Chernomor, von Haeseler & Minh, 2016; Kalyaanamoorthy et al., 2017) in software IQ-TREE 1.6.8 multicore version 1.6.8 (Nguyen et al., 2015).

Phylogenetic relationships were inferred for all genes (nuclear and mitochondrial) concatenated using maximum likelihood as optimality criterion. To find the best tree we used IQ-TREE under default settings. To evaluate branch support, we made 200 non-parametric bootstrap searches and 1,000 searches for the approximate likelihood ratio test also in IQ-TREE (-b and -alrt commands, respectively).

To determine possible inconsistencies in tree topology, we carried out two independent phylogenetic analyses (nuclear and mitochondrial separately) using maximum likelihood as optimality criterion with the same parameters and on the same server as used for the concatenated search. To calculate uncorrected *p-*distances of 16S we used MEGA 7.0 (Kumar, Stecher & Tamura, 2016). Species were delimited using integrative taxonomy criteria (Dayrat, 2005) by combining morphological and genetic evidence.

### Morphology

Only adults and well-preserved specimens were examined. Morphological descriptions follow Lynch & Duellman (1997) format. The terminology and definition of diagnostic characters follows Duellman & Lehr (2009). Sex was determined by gonadal inspection and by the presence of vocal slits in males. Adulthood in males was assessed by examining secondary sexual characteristics (presence of nuptial pads and/or vocal slits) and testes size; reproductively active males have larger and more swollen testes (Duellman & Lehr, 2009). Adulthood in females was determined by examining the convolution of the oviducts and the presence of ovarian eggs (Duellman & Lehr, 2009).

Our species descriptions follow Vences (2020) recommendations to speed up the inventory of species on Earth, namely emphasizing Diagnosis over descriptions and images over words. To streamline the description of the new species and avoid subjectivity in verbal descriptions of color, we only mention color information in the “Comparison with other species” and “Diagnosis” sections; instead, we provide high-resolution photographs of holotypes and several paratypes to show coloration variation in life (when available) and in color descriptions are based on digital photographs and field notes from Elicio Tapia. We describe coloration in life unless otherwise noticed. The following morphological variables were measured in the holotypes using digital calipers (±0.01 mm): SVL (snout-vent length), TL (tibia length), FL (foot length, distance from proximal margin of inner metatarsal tubercle to tip of Toe IV), HL (head length, distance from angle of jaw to tip of snout), HW (head width, at level of angle of jaw), ED (eye diameter, distance between the anterior and posterior borders of the visible eye), TD (tympanum diameter, horizontal distance between the peripheral borders of the tympanic annulus), IOD (interorbital distance, distance between the medial edge of the orbits), EW (upper eyelid width, perpendicular distance to the outer edge of the eyelid), IND (internarial distance, distance between the inner edges of narial openings), EN (eye-nostril distance, distance between the anterior corner of orbit and the posterior margin of the narial opening). TD was measured also for *P. anaiae* sp. nov. and *P. glendae* sp. nov. paratypes for being the species with the smallest tympanum. For all paratypes (113 in total) only SVL was measured. We did not measure other morphometric variables in the type series because previous taxonomic reviews of *Pristimantis* have shown that most morphometric variables are of low diagnostic value (Páez & Ron, 2019; Carrión-Olmedo & Ron, 2021).

Fingers and toes are numbered from inner to outer from I to IV and I to V respectively. Lengths of Toes III and V were determined when both were adpressed against Toe IV; lengths of Fingers I and II were compared when appressed against each other.

Examined specimens (Tables S2–S4) are stored in the herpetological collection of the Museo de Zoología, Pontificia Universidad Católica del Ecuador, Quito, Ecuador (QCAZ).

#### Osteology and geometric morphometry

We described the cranium, postcranium and osteology of the hand and foot of two of the new species using high-resolution x-ray computed tomography (CT-scanning) images (available at: https://doi.org/10.5281/zenodo.6323699). We could not obtain CT-scans of the remaining new species because we were unaware of them at the time when we had access to the CT-scan. We also made exploratory comparisons between *P. tamia* sp. nov. and their closest relatives using the same images. The scans were made at the Department of Ecology & Evolutionary Biology of Toronto University using a Bruker SkyScan 1173 X-ray Micro-CT scanner. To avoid movements and drying of the specimens during scanning, each individual was placed in a small cylindrical plastic container and mounted with cling wrap. The scanner was set at a source voltage of 45 kV, current of 170 uA, without filters and with a pixel size of 50.0 um. The scans were made at rotations steps of 0.2 degrees in a round trajectory with the “step and shoot” motion. Each scan was made in ~38 min with an exposure time of 950 ms. The CT-dataset was reconstructed using N-Recon software (Bruker MicroCT) and rendered in three dimensions with the Stratovan CheckPoint software.

**Figure 2:**
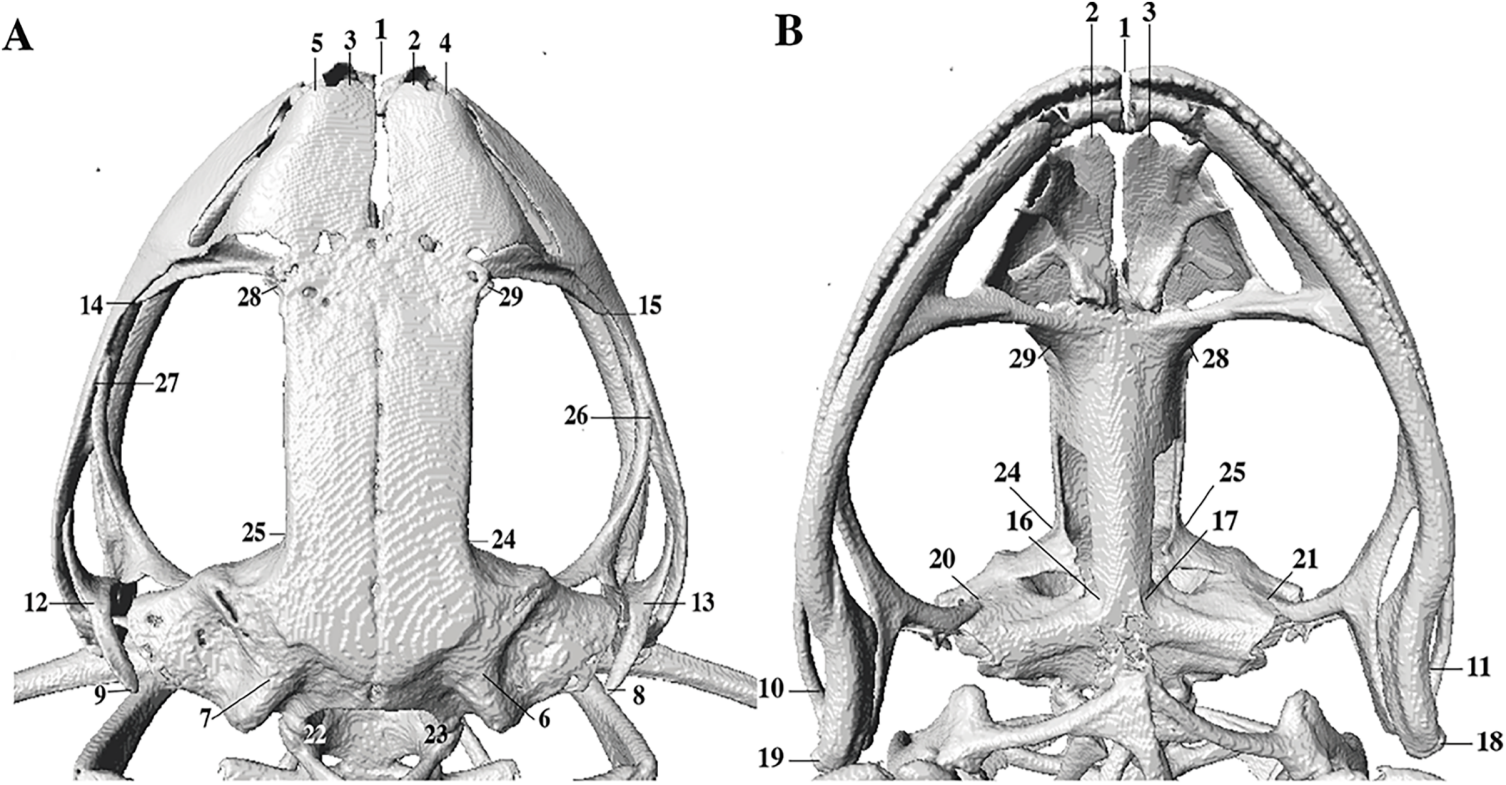
Dorsal and ventral views of an anuran skull. The lines point the places where the landmarks were set, principally articulations, projections and bone angles. (A) Dorsal view. (B) Ventral view.

**Figure 3:**
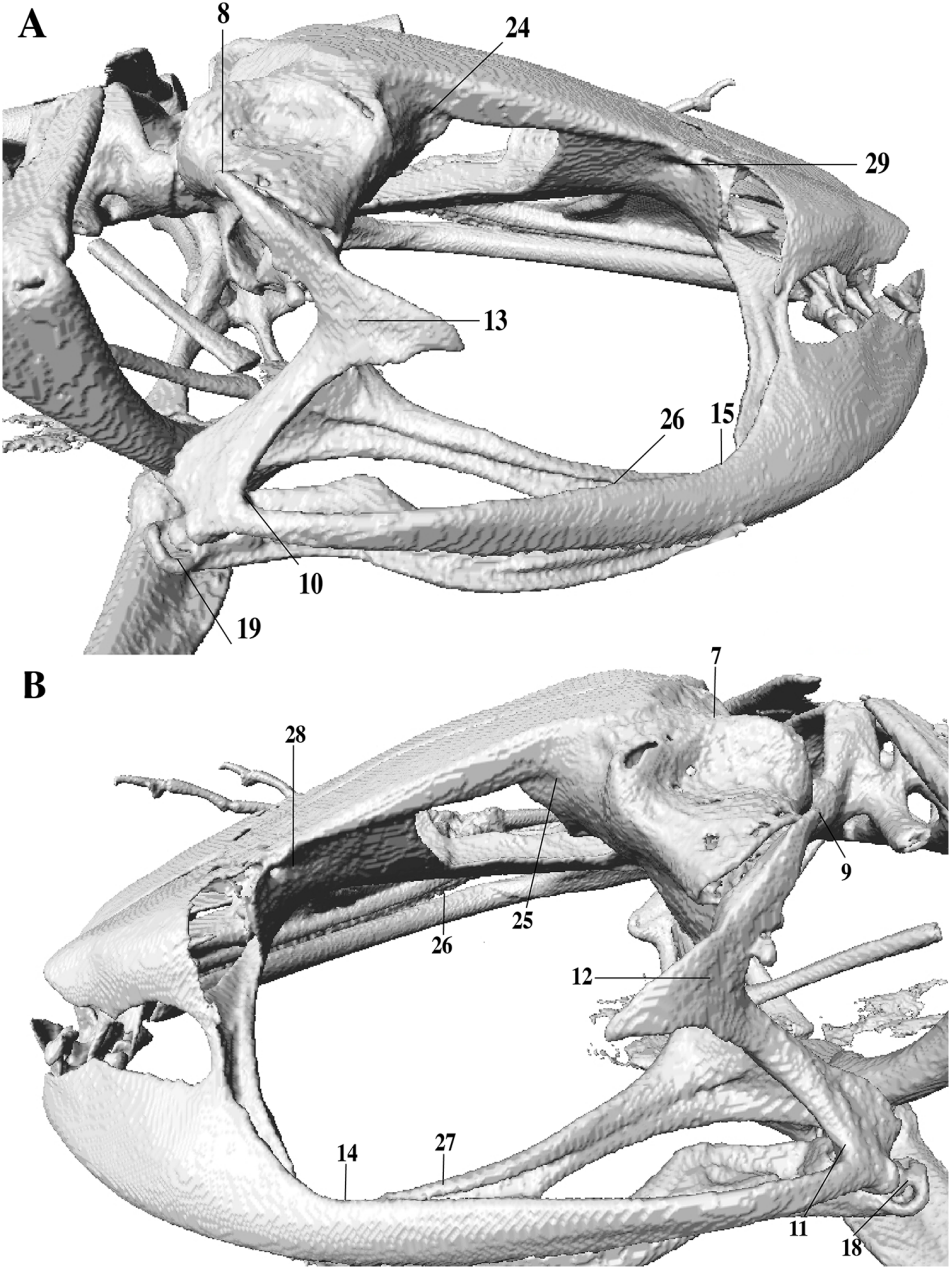
Lateral view of anuran skull. The lines point the places where the landmarks were set, principally articulations, projections and bone angles. (A) Right lateral view. (B) Left lateral view.

In the geometric morphometric analysis, landmarks (Figs. 2, 3) were set in Stratovan CheckPoint software and located, primarily, in joints, bone projections and bone angles following Ponssa & Candioti (2012) and Acevedo, Lampo & Cipriani (2016): (1) premaxillary suture; (2) rostral end of right nasal; (3) rostral end of left nasal; (4) right lateral end of nasal; (5) left lateral end of nasal; (6) crest between frontoparietal and right prootic; (7) crest between frontoparietal and left prootic; (8) otic ramus of right squamosal; (9) otic ramus of left squamosal; (10) angle at the anterior end of quadratojugal-squamosal articulation (right side); (11) angle at the anterior end of quadratojugal-squamosal articulation (left side); (12) point between vertical and transverse branch of left squamosal; (13) point between vertical and transverse branch of right squamosal; (14) lower rostral angle of the left orbit; (15) lower rostral angle of right orbit; (16) vertex between right ala and cultriform process of parasphenoid; (17) vertex between left ala and cultriform process of parasphenoid; (18) caudal end of left angulosplenial; (19) caudal end of right angulosplenial; (20) right articulation medial to the junction point between vertical and transverse branch of squamosal; (21) left articulation medial to the junction point between vertical and transverse branch of squamosal; (22) left atlanto-occipital articulation; (23) right atlanto-occipital articulation; (24) upper caudal angle of right orbit; (25) upper caudal angle of left orbit; (26) rostral angle of right maxilla; (27) rostral angle of left maxilla; (28) upper rostral angle of left orbit; and (29) upper rostral angle of right orbit.

Data obtained from landmarks was exported in Morphologika format (raw data for geometric morphometrics landmarks is available at: https://zenodo.org/record/6323456) and analyzed using a PCA in RStudio using commands g.pagen, pro$Csize and plotTangentSpace available in the Geomorph 3.2.1 Package (Adams, Collyer & Kaliontzopoulou, 2020). One PCAs was performed to compare *P. tamia* sp. nov. cranium with its closest species.

## Results

### Phylogenetic analyses and species delimitation

The concatenated matrix was 4,155 bp in length for 235 samples (available at: http://doi.org/10.5281/zenodo.7055289). IQTree chose nine partitions for the analyses based on the concatenated matrix of mitochondrial and nuclear genes, three partitions for the analyses based on RAG1 only, and six partitions for the analyses based on mitochondrial genes only (Table S5). The phylogenetic trees obtained with the three matrixes are congruent in topology, except for some of the less supported relationships (shown in red in Fig. S1 and Fig. S3-available at: https://doi.org/10.5281/zenodo.6484936), other relationships obtained were consistent among phylogenies.

The phylogenetic relationships inferred with the concatenated matrix were generally congruent with those of Hedges, Duellman & Heinicke (2008), the “tree-alignment + parsimony” of Padial, Grant & Frost (2014), Páez & Ron (2019), and the phylogeny of Carrión-Olmedo & Ron, 2021; topological differences pertain mainly to nodes with low support values (Fig. S1-available at: https://doi.org/10.5281/zenodo.6484936). Comparisons of our phylogeny with previous ones (*e.g*., Hedges, Duellman & Heinicke, 2008; Padial, Grant & Frost, 2014) requires taking into account numerous identification errors on specimens used in the phylogenetic analyses. For example, similarly to us, Hedges, Duellman & Heinicke (2008; pp. 18) found *Huicundomantis* as a strongly supported clade composed of five species. However, three of those species were incorrectly identified: “*P. riveti*” was in fact *P. gloria*, “*P. cryophilius*” was *P. philipi*, and “*P. phoxocephalus*” was *P. totoroi* (see Páez & Ron, 2019 for details). The misidentification of the three specimens persists until now in the GenBank database.

**Figure 4:**
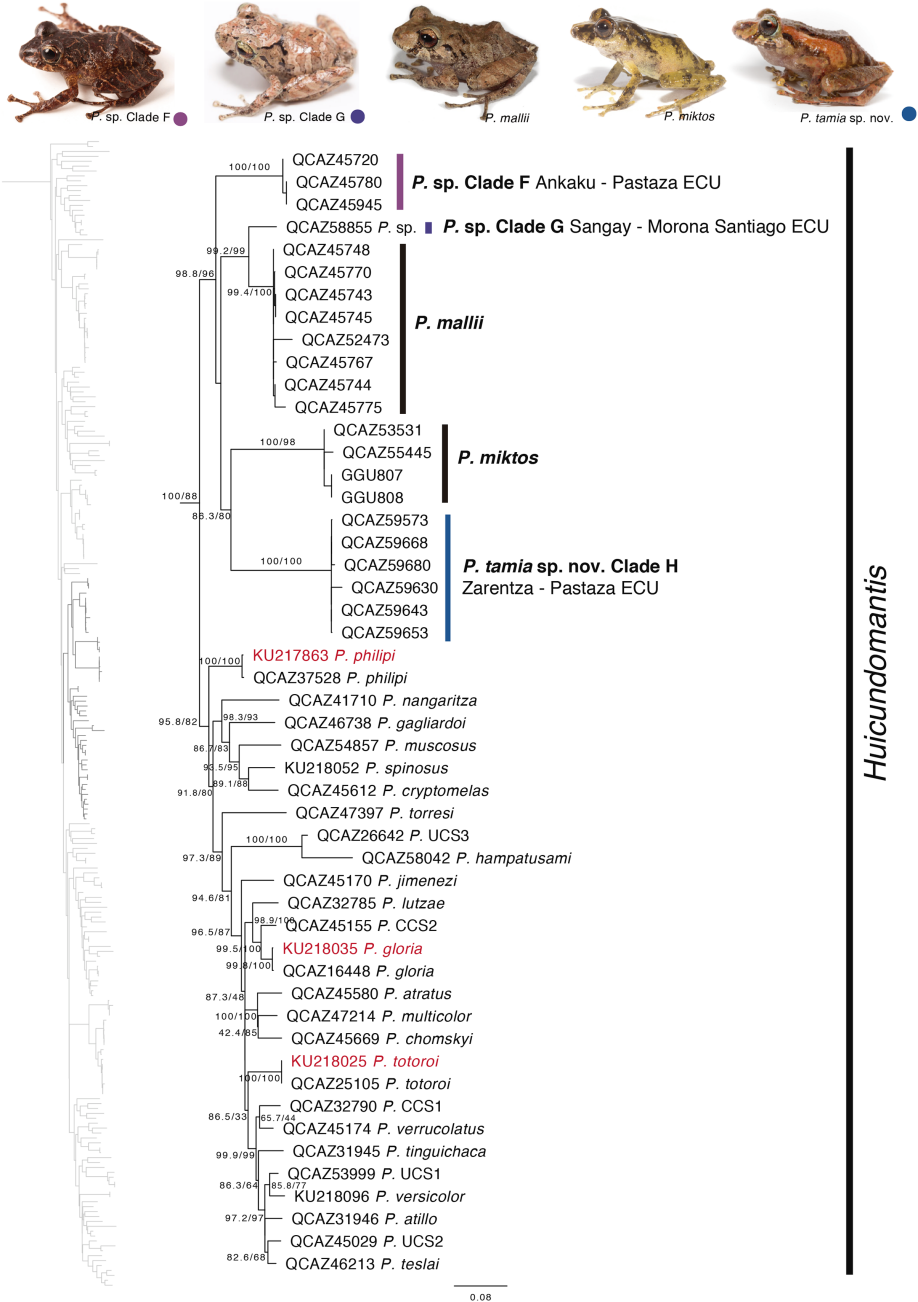
Phylogenetic relationships of *Pristimantis miktos* species group. Maximum likelihood tree obtained for genes 16S, 12S, ND1 and RAG1. Support values are on the corresponding branches: aLRT values before the slash and bootstrap after the slash. The phylogeny was derived from an analysis of 4,155 bp for 235 samples of mitochondrial (gene fragments 12S, 16S and ND1) and nuclear (gene fragments RAG1) DNA sequences. For each specimen, the museum number is shown. Specimens shown in red are those that have a different identification than the one given in the Genbank. Outgroup is not shown. Abbreviations: ECU, Ecuador.

Our phylogeny shows strong support for two previously unknown clades. The first clade is distributed in the eastern Andean slopes of central and southern Ecuador and the Amazonian lowlands of Ecuador (in Tungurahua and from Orellana to Morona Santiago provinces). It is sister to the subgenus *Huicundomantis* and includes *P. mallii* Reyes-Puig et al., 2019, *P. miktos* Ortega-Andrade & Venegas, 2014, a new species that we describe here as *P. tamia* sp. nov., and two seemingly undescribed species (clades F and G) (Fig. 4). Relationships among species within the clade are strongly supported. Mean pairwise genetic distance (uncorrected *p* for gene 16S) between *P. tamia* sp. nov. and its sister species, *P. miktos*, is 9% (Table 2).

**Table 2:** Pairwise genetic distances between species of the *Pristimantis miktos* species group.

	*P. miktos*	*P. tamia*	*P. mallii*	*P*. sp. Clade G	*P*. sp. Clade H
*P. miktos* (2)		**1.3**	**1.4**	**1.1**	**1.3**
*P. tamia* (39)	9.4		**1.2**	**1.2**	**1.2**
*P. mallii* (8)	8.0	12.0		**0.9**	**1.1**
*P. sp. Clade G* (1)	7.9	11.7	6.1		**1.1**
*P. sp. Clade H* (3)	9.2	11.0	10.9	9.5	

**Note:**

Mean uncorrected *p* distances (gene 16S) between species of the *P. miktos* species group. Mean uncorrected *p* distances (%) between groups are shown under the diagonal. Standard error estimates in bold above the diagonal. Number of samples is given in brackets after the name of each clade. Standard error estimates were obtained by a bootstrap procedure in MEGA 7.0.

**Figure 5:**
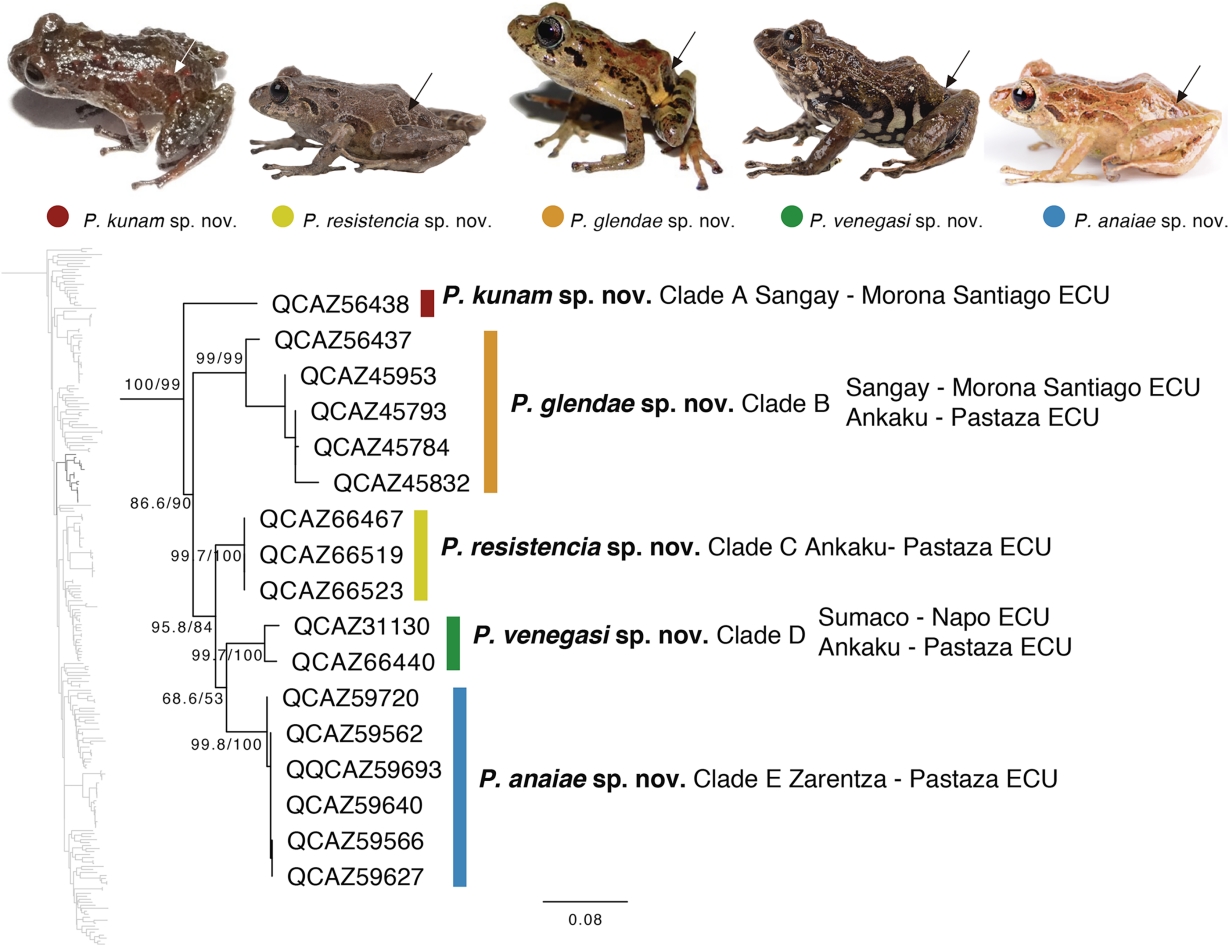
Phylogenetic relationships of *Pristimantis anaiae* species group. Maximum likelihood tree obtained for genes 16S, 12S, ND1 and RAG1. Support values are on the corresponding branches: aLRT values before the slash and bootstrap after the slash. The phylogeny was derived from an analysis of 4,155 bp for 235 samples of mitochondrial (gene fragments 12S, 16S and ND1) and nuclear (gene fragments RAG1) DNA sequences. For each specimen, the museum number is shown, as well as its locality. Outgroup is not shown. Abbreviations: ECU, Ecuador. Arrows in the specimen photos show the putative synapomorphy of the *P. anaiae* species group: the presence of a dark sacral spot surrounded by a lighter border.

The second clade is distributed in cloud forests of the Amazonian Andean slopes of central Ecuador (Napo to Morona Santiago provinces). Remarkably, this clade is composed exclusively by new species, five in total (Fig. 5). Herein we describe them as *P. anaiae* sp. nov., *P. glendae* sp. nov., *P. kunam* sp. nov., *P. resistencia* sp. nov., and *P. venegasi* sp. nov. All species share a putative synapomorphy, the presence of large sacral dark round areas with thin clear borders; confirmation of this character as synapomorphy for the group would require trace its evolution on a phylogeny. Unfortunately, the position of this clade within *Pristimantis* (Fig. 5) has weak support. The new species are separated from each other by pairwise genetic distances (uncorrected *p* for gene 16S) >3% (Table 3). This clade is sister to a large clade composed of the subgenus *Huicundomantis*, the *P. orestes* species group and several species that were not assigned to a species group by Padial, Grant & Frost (2014) (*e.g*., *P. librarius, P. lirellus, P. ockendeni, P. orestes, P. pardalis, P. parvillus, P. platydactylus, P. quaquaversus, P. rhodoplichus, P. simonsii, P. zophus*).

**Table 3:** Pairwise genetic distances between species of the *Pristimantis anaiae* species group.

	*P. anaiae*	*P. glendae*	*P. kunam*	*P. resistencia*	*P. venegasi*
*P. anaiae* (2)		**0.0133**	**0.0149**	**0.0092**	**0.0123**
*P. glendae* (4)	0.0895		**0.0144**	**0.0138**	**0.0131**
*P. kunam* (1)	0.1040	0.1013		**0.0144**	**0.0145**
*P. resistencia* (3)	0.0406	0.0947	0.0919		**0.0110**
*P. venegasi* (2)	0.0755	0.0921	0.1021	0.0625	

**Note:**

Mean uncorrected *p* distances (gene 16S) between species of the *P. anaiae* species group. **Distances are shown under the diagonal, standard errors, in bold, above the diagonal. Number of samples is given in parentheses after the name of each species. Standard error estimates were obtained by bootstrap in MEGA 7.0.

**Figure 6:**
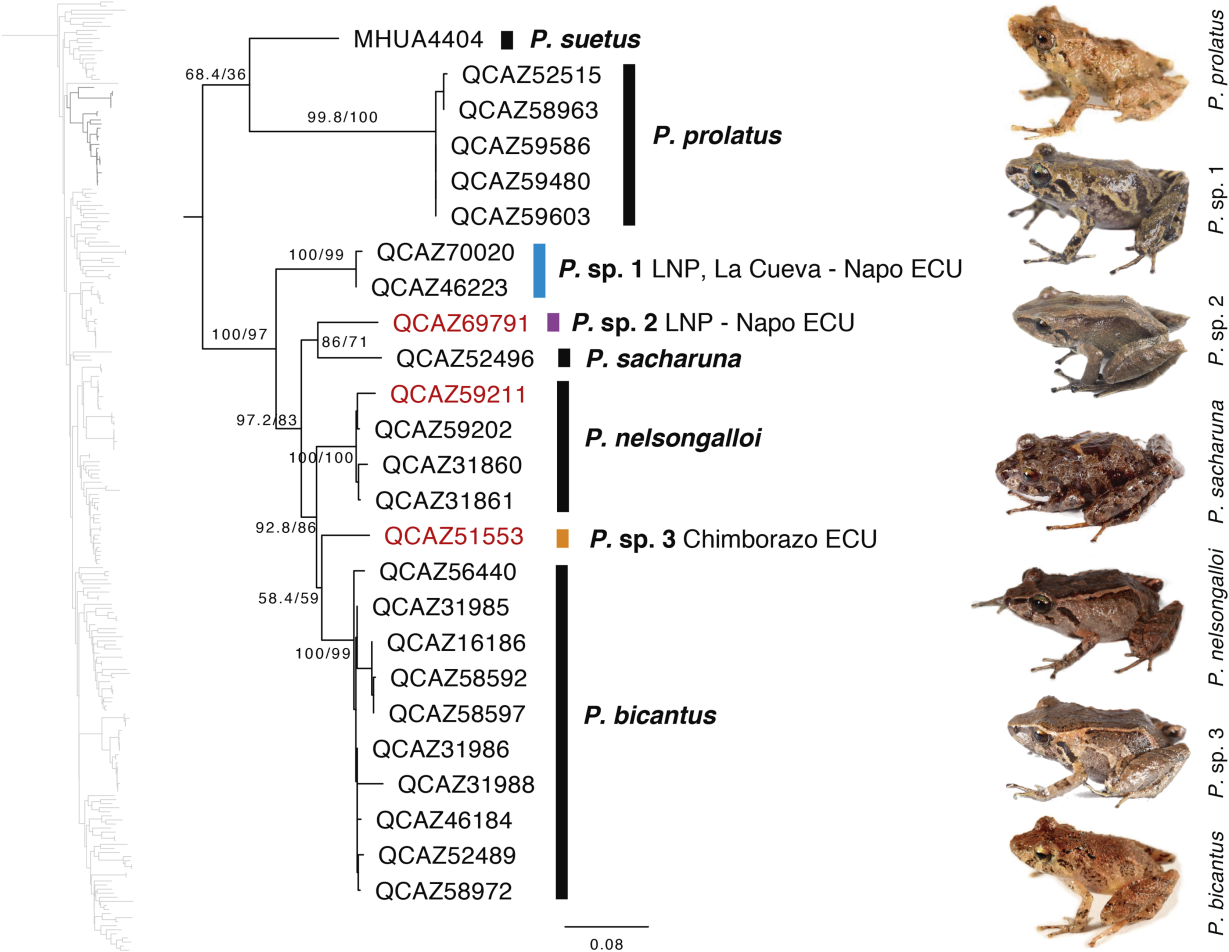
Phylogenetic relationships of *Pristimantis sacharuna*, *Pristimantis nelsongalloi*, and *Pristimantis bicantus*. Maximum likelihood tree obtained for genes 16S, 12S, ND1 and RAG1. Support values are on the corresponding branches: aLRT values before the slash and bootstrap after the slash. The phylogeny was derived from an analysis of 4,155 bp for 235 samples of mitochondrial (gene fragments 12S, 16S and ND1) and nuclear (gene fragments RAG1) DNA sequences. For each specimen, the museum number is shown. Specimens shown in red are those that have a different identification than the one given in the Genbank. Outgroup is not shown. Abbreviations: ECU, Ecuador; LNP, Llanganates National Park.

Our results show, for the first time, the phylogenetic relationships of *P. bicantus*, *P. nelsongalloi*, and *P. sacharuna* (Fig. 6). The three species are closely related and are part of a well-supported clade composed of three additional species, all of them are undescribed.

### Geometric morphometry

**Figure 7:**
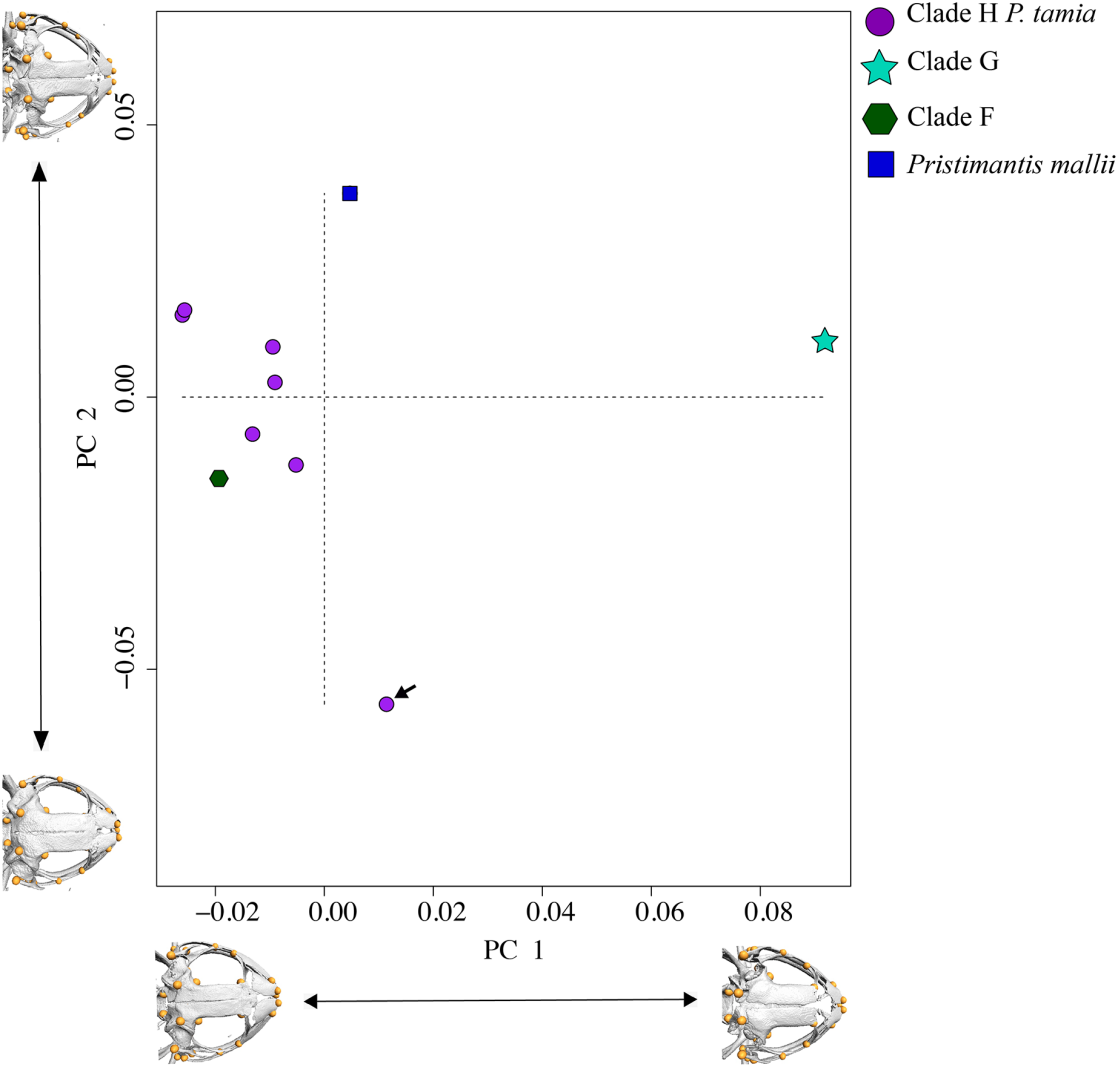
PCA showing skull differences between *P. tamia* and its closest relatives. PCA accounted for 53.8% of the total variation. PC1 explains 35.1% of the variance and PC2, 18.7%. The highest percentage of variation occurs, in the first and second components at the level of the ocular cavity (landmark 29). The arrow shows the male of *Pristimantis tamia* analyzed.

In morphometric space, *Pristimantis tamia* sp. nov. is closer to *P. mallii* and *P*. Clade F (Fig. 7). The only male of *Pristimantis tamia* sp. nov. included in the analysis (QCAZ 59713) separates from the adult females. The analysis also revealed similarity between the skulls of female *P. tamia* sp. nov. and Clade F (within the morphometric space, skulls belonging to female *P. tamia* are grouped with the skull of *P*. Clade F). The only individual of clade G (QCAZ 58855) had the lowest degree of ossification (skull shown on the right end of PC1) and is also most divergent in morphometry (Fig. 7). In this analysis, PC1 and PC2 accounted for 53.8% of the total variation. PC1 explains 35.1% of the variance and PC2, 18.7%. The landmarks that provide the greatest variation in the first and second components are related to the ocular cavity, the otoccipital crests and the mandibular joint (Fig. 7 and Table S6).

### Systematic accounts

*PRISTIMANTIS ANAIAE* SPECIES GROUP

*Definition*: The *Pristimantis anaiae* species group is strongly supported in our phylogeny. Members of this group share the following morphological traits: (i) small frogs with SVLs from 13.08 to 20.63 mm in males and 24.59 to 34.90 mm in females; (ii) slender bodies; (iii) dorsum shagreen to tuberculate; (iv) discoidal fold present or not; (v) dorsolateral folds absent; (vi) supratympanic fold present; (vii) tympanic annulus present, tympanic membrane absent or present; (viii) snout short and rounded or broadly rounded in dorsal view and rounded in lateral view; (ix) cranial crests absent; (x) upper eyelid and heels bearing tubercles (tubercles in heels absent only in *P. glendae*); (xi) vocal slits and nuptial pads absent; (xii) basal webbing between toes absent or present; (xiii) Finger I shorter than Finger II, discs of digits expanded to broadly expanded, truncate; (xiv) Toe V much longer than Toe III, Toe Condition C (Toe III reaches the distal border of the distal subarticular tubercle of Toe V and the proximal border of the penultimate tubercle of Toe IV; Toe V reaches the middle of the distal tubercle of toe IV); (xv) all fingers and toes bearing lateral fringes, supernumerary tubercles, and elongated to rounded hyperdistal tubercles; (xvi) sacral round to elongated dark areas with thin clear borders present on each side (Fig. 5); (xvii) iris with black reticulation.

*Content*: The *Pristimantis anaiae* species group comprises five species described below: *P. anaiae* sp. nov., *P. glendae* sp. nov., *P. kunam* sp. nov., *P. resistencia* sp. nov., and *P. venegasi* sp. nov.

*Distribution* (Fig. 1): Eastern Andean slopes of central Ecuador, in two provinces: Morona Santiago and Pastaza. Species inhabit Eastern Montane Forest (Ron, Merino-Viteri & Ortiz, 2021), between 1,350–2,451 m.a.s.l. They have been found on low vegetation from the ground up to a height of 300 cm in cloud forests.

*Remarks*: According to our phylogeny, the *P. anaiae* species group is strongly supported. However, its relationships to other clades of *Pristimantis* are weakly supported.

***Pristimantis anaiae* sp. nov.**

urn:lsid:zoobank.org:act:020CF5BA-B737-4F45-B9B9-209A9F1AE223

**Figure 8:**
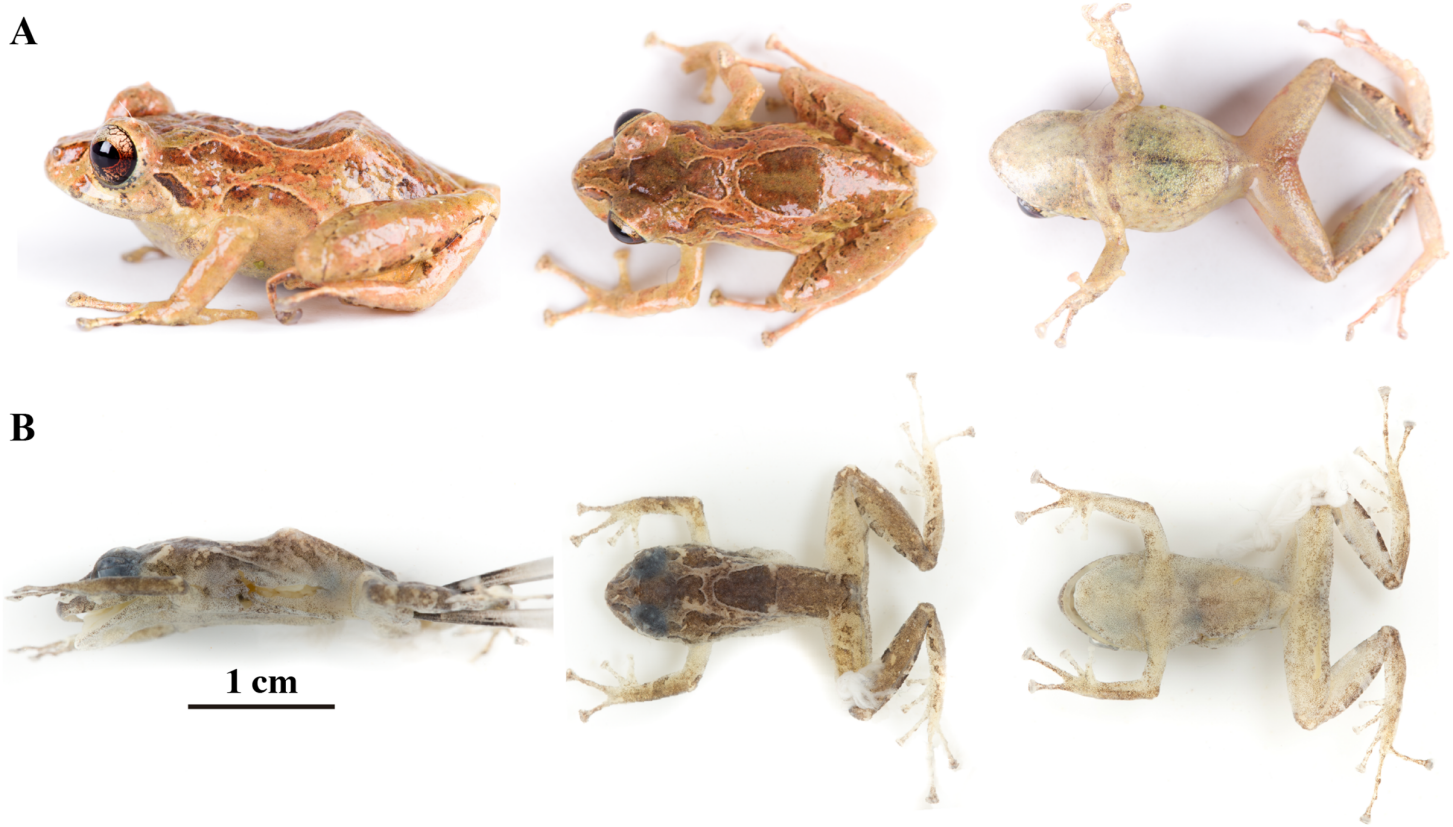
*Pristimantis anaiae* sp. nov. Holotype, QCAZ 59693, adult male, SVL = 18.07 mm. (A) Photographs of alive individual in lateral, dorsal and ventral view. (B) Photographs of preserved individual on lateral, dorsal and ventral view. Scale is given for dorsal view photograph of preserved individual only.

**Figure 9:**
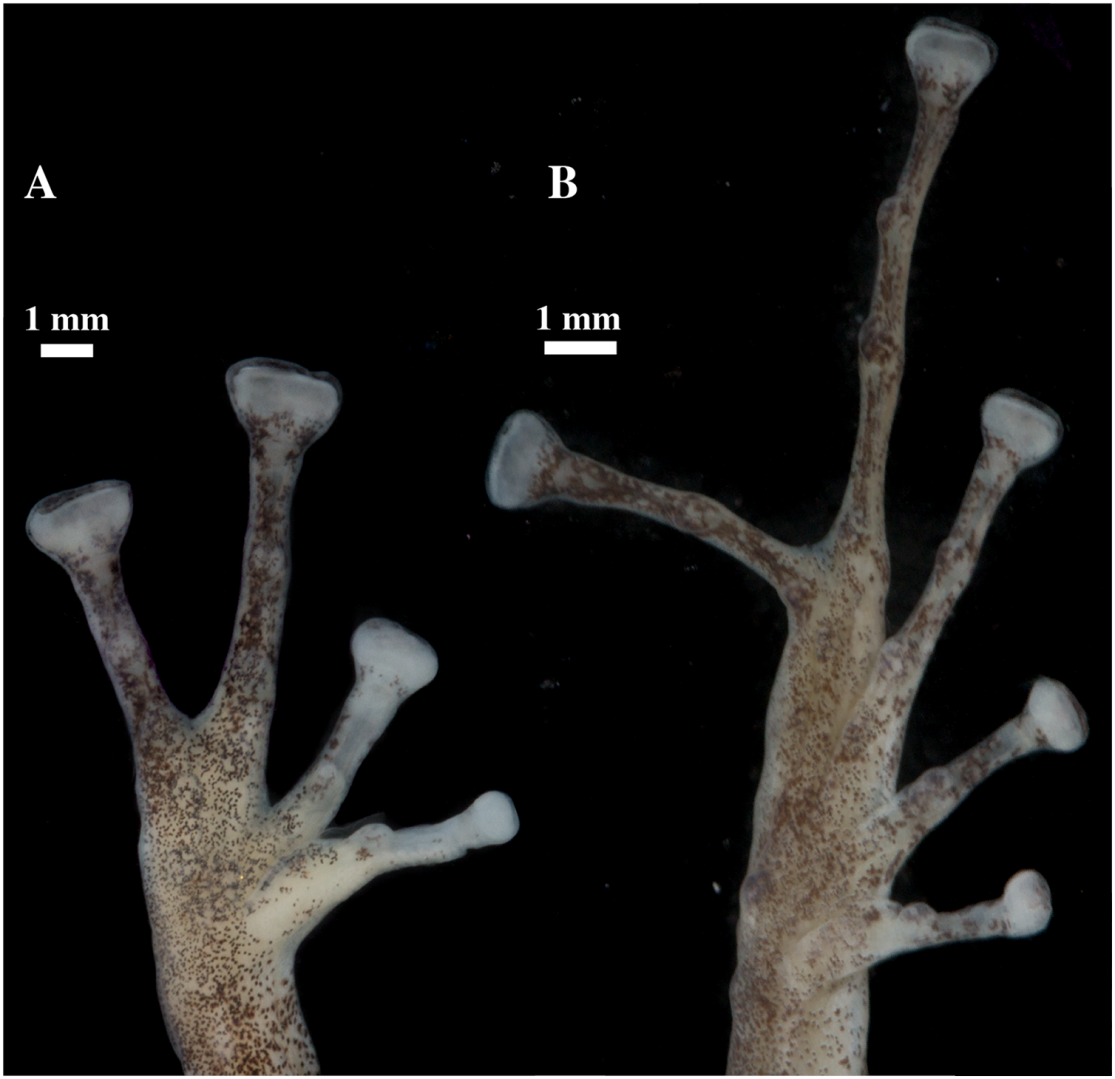
(A) Palmar and (B) plantar surfaces of *Pristimantis anaiae* sp. nov. Photographs of right hand and foot of the holotype QCAZ 59693.

**Figure 10:**
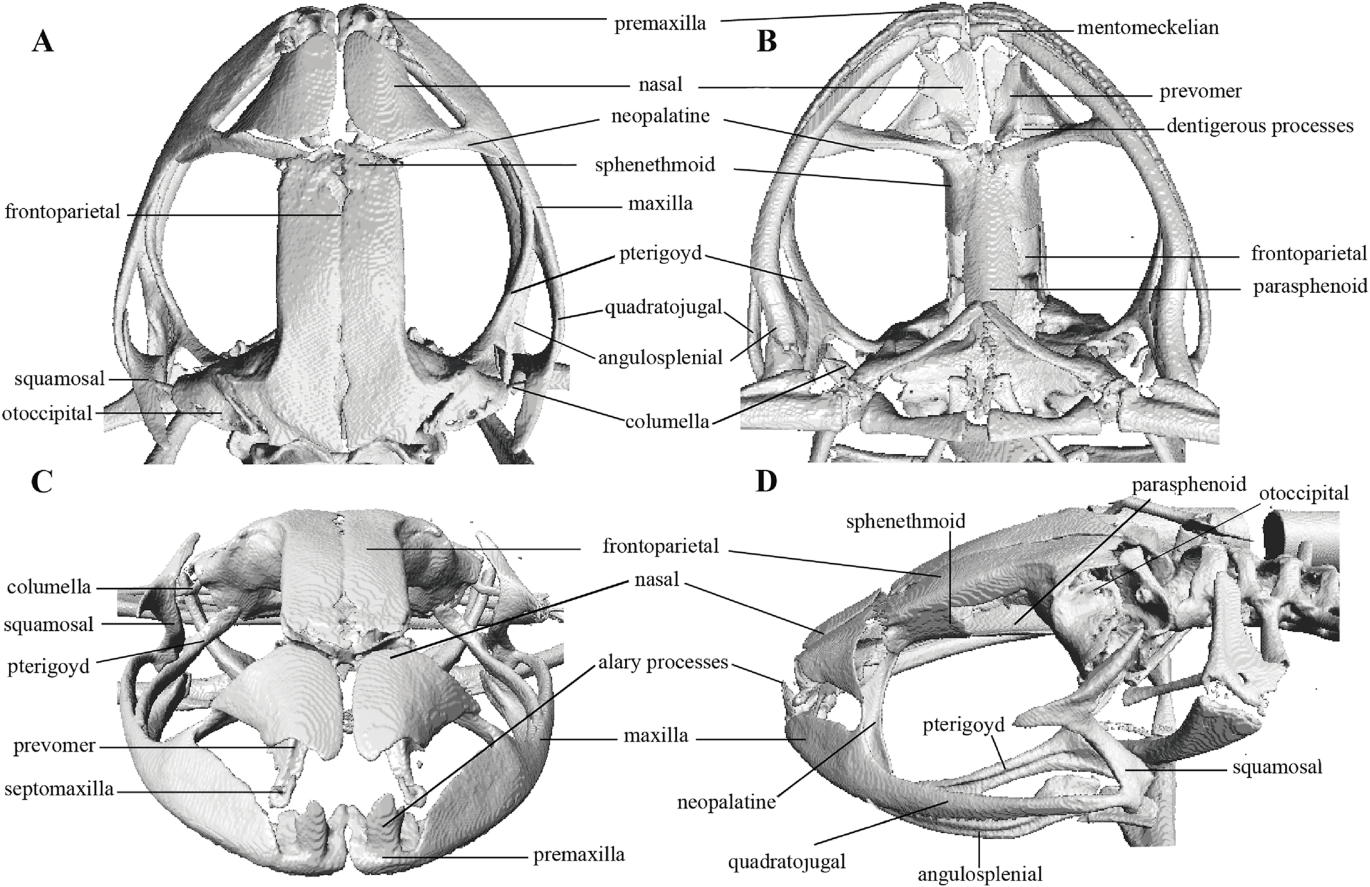
Head skeleton of *Pristimantis anaiae* sp. nov. Holotype QCAZ 59693. The skull is shown in: (A) dorsal view; (B) ventral view; (C) frontal view; (D) lateral view.

**Figure 11:**
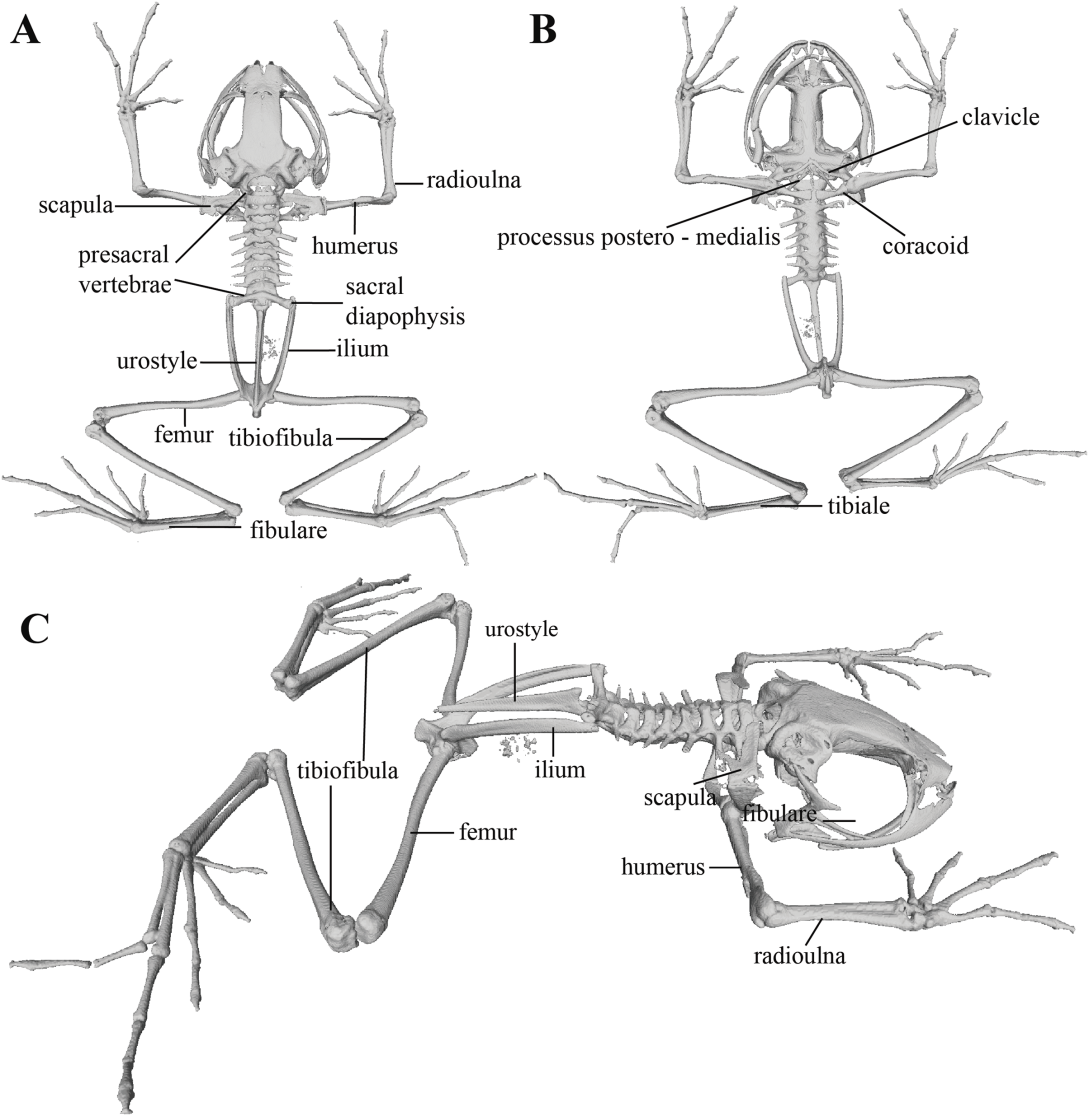
Whole skeleton of *Pristimantis anaiae* sp. nov. Holotype QCAZ 59693. The full skeleton is shown in: (A) dorsal view. (B) ventral view. (C) dorsolateral view.

**Figure 12:**
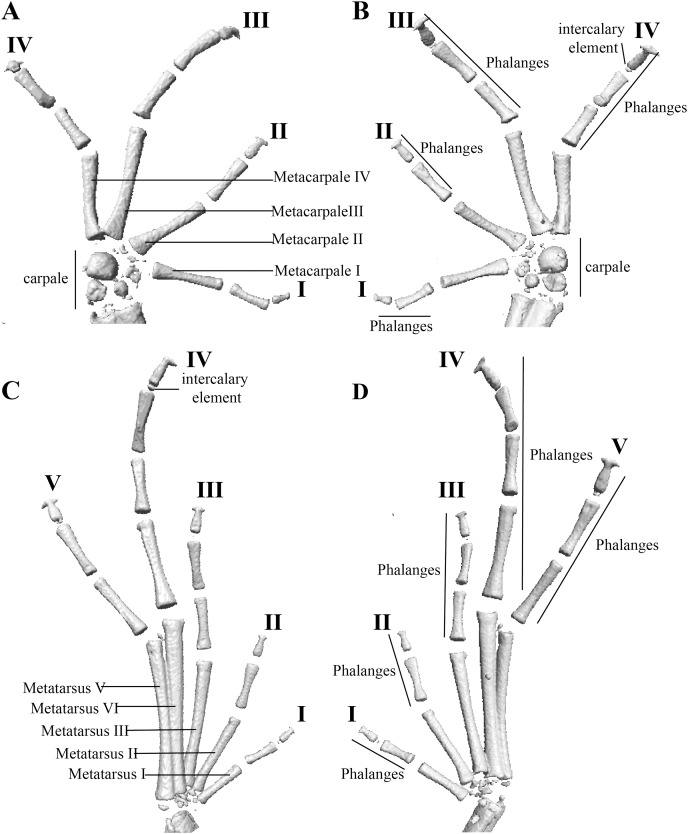
Hand and foot osteology of *Pristimantis anaiae* sp. nov. Paratype QCAZ 59640. The left hand is shown in (A) dorsal and (B) ventral (palmar) views. The left foot is shown in (C) dorsal and (D) ventral (plantar) views.

**Holotype (Figs. 8–12):** QCAZ 59693 (field no. SC-PUCE 49892), adult male from Ecuador, Pastaza Province, Canton Mera, Llanganates National Park, Zarentza Community, path to the Yurugyaku river (1.3397° S; 78.0594° W), 1,367 m, collected by Daniel Rivadeneira, Francy Mora, Juan Carlos Sánchez, David Velalcázar, Darwin Núñez, and Javier Pinto on February 17, 2015.

**Figure 13:**
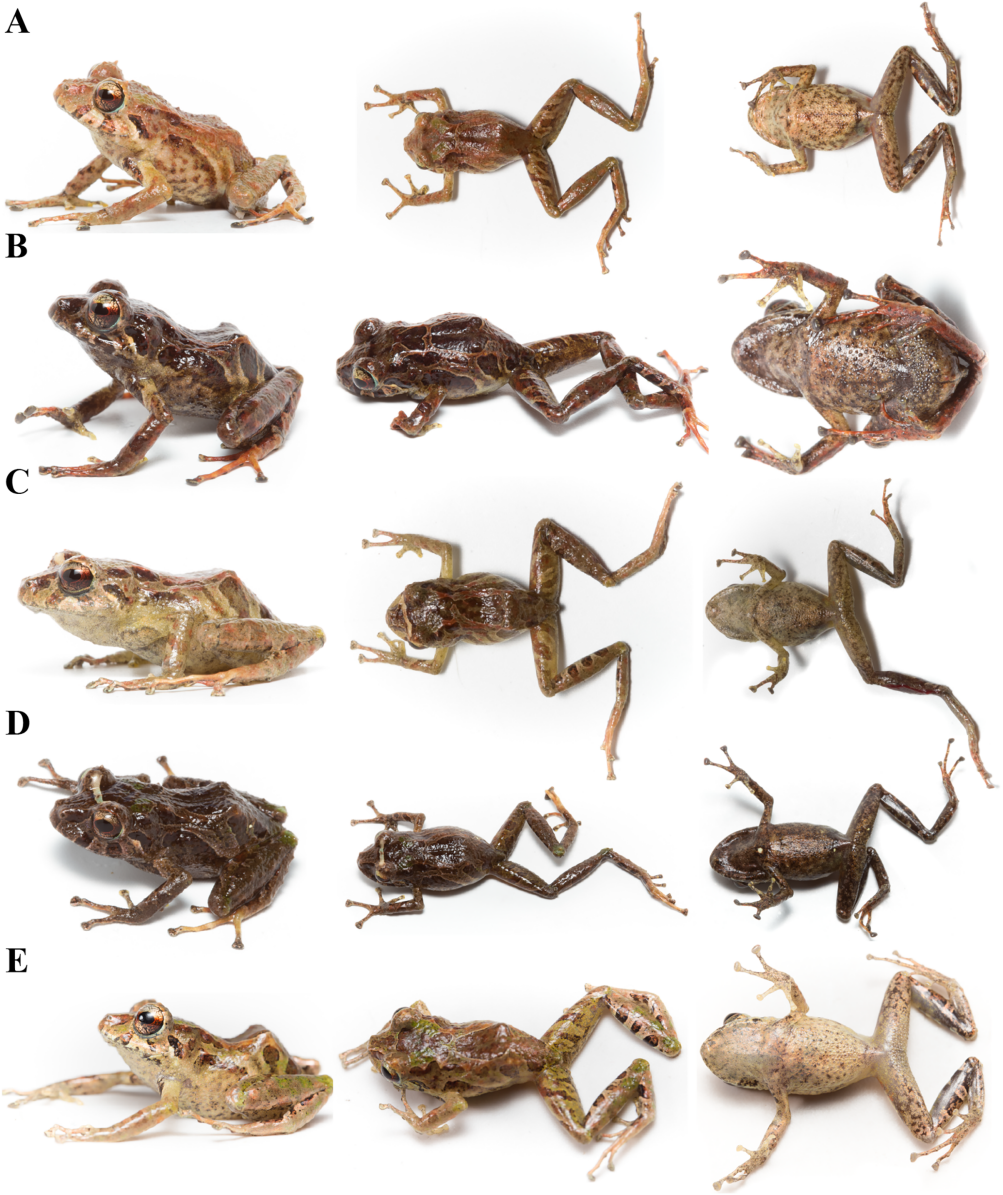
Color variation in life individuals of *Pristimantis anaiae* sp. nov. (A) QCAZ 59640, paratype, adult male, SVL = 20.63 mm. (B) QCAZ 59562, paratype, adult male, SVL = 17.21 mm. (C) QCAZ 59627, paratype, adult male, SVL = 18.73 mm. (D) QCAZ 59566, paratype, adult male, SVL = 17.02 mm. (E) QCAZ 59720, paratype, adult male, SVL = 13.08 mm. Lateral view on the left, dorsal view in the center and ventral view on the right.

**Figure 14:**
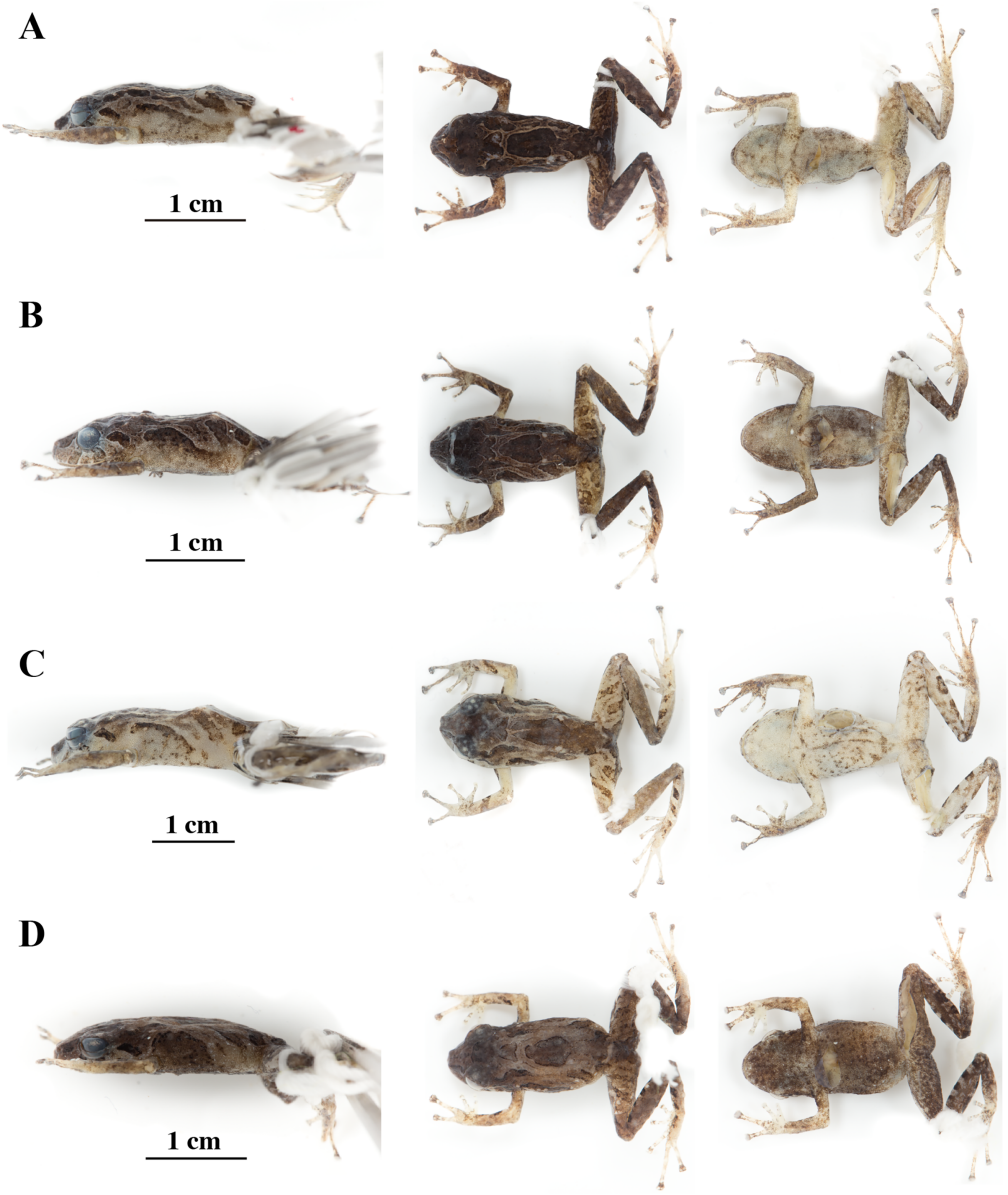
Color variation in preserved individuals of *Pristimantis anaiae* sp. nov. (A) QCAZ 59562, paratype, adult male, SVL = 17.21 mm. (B) QCAZ 59566, paratype, adult male, SVL = 17.02 mm. (C) QCAZ 59640, paratype, adult male, SVL = 20.63 mm. (D) QCAZ 59720, paratype, adult male, SVL = 13.08 mm. Lateral view on the left, dorsal view in the center and ventral view on the right. Scales are given for dorsal view photographs of preserved individuals only.

**Paratypes (*n* = 5; Figs. 13, 14):** All from Ecuador; Pastaza Province, Canton Mera, Llanganates National Park, Zarentza Community. School surroundings, QCAZ 59562, QCAZ 59566 adult males, 1.3564° S, 78.0581° W, 1,367 m. Tributaries of the river Nuchimingue, QCAZ 59627 adult male, 1.3626° S; 78.0582° W, 1,350 m, QCAZ 59720 adult male 1.3626° S 78.0578° S, 1,391 m. Trail to the Yurugyaku river, QCAZ 59640 adult male 1.3472° S; 78.0813° W, 1,380 m. Same collectors as for the holotype, on February from 14 to 27 of 2015.

**Common name:** English: Anaí Rain Frog. Spanish: Cutín de Anaí.

**Diagnosis (Figs. 8–14):** We assign the new species to the genus *Pristimantis* based on the phylogeny (Fig. 5). A species of *Pristimantis* characterized by: (1) skin of dorsum shagreen sometimes bearing large tubercles, skin on throat smooth, skin on belly areolate bearing scattered tubercles; (2) discoidal fold present; (3) dorsolateral folds absent; (4) tympanic membrane absent, tympanic annulus small (on average, 2.5% of SVL); (5) inconspicuous supratympanic fold might be present; (6) postrictal tubercle present; (7) snout short and rounded in dorsal and lateral view; (8) upper eyelid with one conical tubercle and few lower tubercles; (9) cranial crests absent; (10) vocal slits and nuptial pads absent; (11) Finger I shorter than Finger II, discs of digits expanded to broadly expanded, truncate; (12) fingers with narrow lateral fringes, all fingers with elongated and thin hyperdistal tubercles; (13) ulnar tubercle prominent and rounded; (14) heel bearing a small conical tubercle; (15) toes with broad lateral fringes, basal webbing present, all toes with elongated hyperdistal tubercles; (16) Toe V much longer than Toe III, Toe condition C (Toe III surpasses the distal border of the distal subarticular tubercle of Toe V and reaches the proximal border of the penultimate tubercle of Toe IV; Toe V reaches the proximal border of the distal tubercle of toe IV); (17) dorsum greenish-brown with an H-shaped greenish-orange mark, a large sacral dark round area with thin clear borders is present on each side. Flanks pale orange to greenish brown bearing a short brown longitudinal stripe or a dark brown circular blotch on its posterior portion. Ventral surfaces cream to dark brown bearing black or dark brown tubercles; iris bronze with a wide copper medial band and black reticulations; light blue sclera; (18) SVL in females unknown; SVL in adult males 17.46 mm on average (range 13.08–20.63; *n* = 6) (Tables 4, S2).

**Table 4:** SVL values (mm) of adult females and males of the new species described.

Species	Sex	SVL average	SVL Min	SVL Max
*P. anaiae*	Male (9)	17.53 (2.1)	13.08	20.63
*P. glendae*	Male (5)	18.72 (1.2)	16.60	19.93
*P. kunam*	Male (1)	14.66	–	–
*P. resistencia*	Female (1)	24.59		
*P. resistencia*	Male (3)	18.90	17.56	19.75
*P. venegasi*	Female (1)	34.90	–	–
*P. venegasi*	Male (1)	24.49	–	–
*P. tamia*	Female (32)	26.63 (2.2)	19.16	29.88
*P. tamia*	Male (66)	18.30 (2.4)	13.69	24.82

**Note:**

Snout-vent length (SVL) in mm and standard deviation (in parentheses) for males and females. Number of individuals is given in parentheses after the sex. For each group, minimum and maximum values are also shown.

**Comparison with other species:** *Pristimantis anaiae* resembles *P. roni* Yánez-Muñoz et al., 2014, *P. yanezi* Navarrete, Venegas & Ron, 2016, and *P. llanganati* Navarrete, Venegas & Ron, 2016 by having upper eyelids with a prominent conical tubercle and wide finger discs. It differs from *P. roni*, *P. yanezi*, and *P. llanganati* by its dorsal coloration pattern, the absence of tympanic membrane and the presence of large sacral dark round areas with thin clear borders (flanks with diagonal bars and presence of tympanic membrane in *P. roni*; dorsum dark brown to yellowish brown with scattered light brown or orange spots, presence of tympanic membrane and flanks olive brown with fringes and diffuse dark diagonal lines in *P. yanezi*; and dorsum olive green with X-shaped or rhomboidal dark brown mark on scapular region, tympanic membrane and flanks dirty white or white with dark brown diagonal stripes in *P. llanganati*). Besides, *P. anaiae* differs from *P. roni* by the absence of cranial crests and vocal slits (both present in *P. roni*) and the presence of basal toe webbing (absent in *P. roni*), and from *P. yanezi* by the presence of iris with black reticulation, lateral fringes, and basal toe webbing (all absent in *P. yanezi*). *Pristimantis anaiae* also resembles *P. katoptroides* (Flores, 1988) by having upper eyelids tuberculated, widely expanded finger discs, and a large sacral dark round area with thin clear borders; it differs from *P. katoptroides* by the presence of cream to dark brown groins (greenish blue groins in *P. katoptroides*), cream venter with conspicuous dark brown to black tubercles (white venter in *P. katoptroides*), and the presence of basal toe webbing (absent in *P. katoptroides*).

*Pristimantis anaiae* sp. nov. also resembles *P. verecundus* (Lynch & Burrowes, 1990) and *P. mutabilis* Guayasamin et al., 2015, from the western foothills of the Andes, by the presence of large sacral dark round areas with thin clear borders. It differs from *P. verecundus* by having cream to brown groins, rounded snout (red groins and subacuminate snout in *P. verecundus*), and by the absence of vocal slits (vocal slits present in *P. verecundus*); and from *P. mutabilis* by the absence of tympanic membrane and vocal slits (both present in *P. mutabilis*).

**Description of the holotype (Figs. 8–12):** Live and preserved coloration is shown in Figs. 8 and 9. Adult male (QCAZ 59693). Measurements (in mm): SVL 18.07; tibia length 9.77; foot length 9.12; head length 6.30; head width 6.65; eye diameter 2.92; tympanum diameter 0.41; interorbital distance 1.83; upper eyelid width 2.21; internarial distance 1.97; eye-nostril distance 1.91.

Head wider than long, wider than body, snout rounded in dorsal view and in profile; canthus rostralis straight in lateral view; loreal region slightly concave; cranial crests absent, upper eyelid bearing one big conspicuous conical tubercle surrounded by few indistinct smaller rounded tubercles; tympanic annulus distinct beneath the skin, more conspicuous on its rostral portion; tympanic membrane absent; one enlarged rounded postrictal tubercle. Supratympanic fold ill-defined. Dentigerous processes of vomers present, oblique, broadly separated, posteromedial to choanae; each vomer bearing several inconspicuous small teeth; vocal slits and nuptial pads absent.

Skin on dorsum shagreen, skin on flanks smooth with some scattered tubercles (low and rounded); dorsolateral folds absent; skin on throat smooth, chest and belly areolate with some black, low, tubercles; discoidal fold present; skin in upper cloacal region areolate. Ulnar tubercle present, conspicuous big and rounded; palmar tubercles low, outer palmar tubercle divided in one lateral rounded portion and one oval medial portion twice the length of the lateral portion, slightly shorter than oval thenar tubercle; subarticular tubercles well-defined, round in ventral and lateral view, all fingers with elongated and thin hyperdistal tubercles; supernumerary tubercles at base of fingers present, distinct; narrow lateral fringes on fingers; Finger I shorter than Finger II; discs on Fingers I and Finger II expanded, discs on Fingers III and IV broadly expanded and truncate; pads on all fingers well-defined and surrounded by circumferential grooves (Fig. 9).

Hindlimbs slender; upper surfaces of hindlimbs irregularly areolate; posterior surfaces of thighs areolate, ventral surfaces of thighs smooth on their rostral portion and areolate posteriorly; heel bearing one low, small conical tubercle; outer surface of tarsus bearing inconspicuous small tubercles; tarsal fold present; inner metatarsal tubercle elongate (0.93 mm), elliptical, rounded in lateral view, three times longer than rounded, well-defined outer metatarsal tubercle (0.34 mm); plantar surface with some supernumerary tubercles; subarticular tubercles well-defined, all toes with elongated hyperdistal tubercles; toes with broad lateral fringes; basal webbing between toes present; discs nearly as large as those on fingers, broadly expanded in all toes specially on Toe IV and V; all discs have pads surrounded by well-defined circumferential grooves; relative lengths of toes I < II < III < V < IV; Toe V much longer than Toe III, Toe condition C (Toe III surpasses the distal border of the distal subarticular tubercle of Toe V and reaches the proximal border of the penultimate tubercle of Toe IV; Toe V reaches the proximal border of the distal tubercle of toe IV) (Fig. 9).

**Variation (Figs. 13, 14):** In this section, traits refer to living individuals unless otherwise stated; we only mention character states not observed in the holotype. Individuals may have an inconspicuous supratympanic fold (*e.g*., QCAZ 59627 and QCAZ 59640). Ulnar tubercle can be prominent (*e.g*., QCAZ 59566). Tympanic annulus can be weakly seen beneath skin (*e.g*., QCAZ 59640 and 59720). Large dorsal tubercles giving a spiny appearance may be present (*e.g*., QCAZ 59640). Discs on Fingers and Toes might be not broadly expanded (*e.g*., QCAZ 59720). Belly tubercles can be more prominent, darker, and bigger (*e.g*., QCAZ 59562).

**Osteology:** The osteological description is based on micro-CT images of the holotype (adult male QCAZ 59693).

Skull (Fig. 10): The skull is wider than long; widest part is between quadratojugal and rostral branch of squamosal. Longest axis of skull, from anterior face of premaxilla to posterior face of exoccipital, is 96.7% of the widest axis. Rostrum is short with a distance from anterior edge of the frontoparietals to anterior face of premaxilla of approximately 28% of the longest axis of skull. Skull is about 77.9% of maximum skull width at level of anterior edge of orbits and 89. 9% at level of midorbit. Posterior edge of orbits is aligned to skull widest part.

Skull contains elements mostly well ossified. Frontoparietals are well-developed, markedly longer than wide, slightly narrower anteriorly and not merged medially; a conspicuous non-ossified fontanelle is present posteriorly. The posterior portion of skull is fully enclosed by complete fusion of frontoparietals with otoccipitals; the latter bear unossified patches on each side. Otoccipitals articulate ventrally with the parasphenoid alae and are formed by well-fused prootics and exoccipitals. Where frontoparietals articulate with otoccipitals, they form prominent crests. Each crest is V-shaped with 90 degrees angle directed medially; its posterior branch is 79.2% the length of anterior branch.

Anteriorly, frontoparietals articulate with a ventrally unossified sphenethmoid. Its posterior margin does not reach midpoint of orbit and is in contact with parasphenoid. Cultriform process of parasphenoid is tapered anteriorly; its base is about 9.8% of maximum skull width and it reaches its greatest width (10.9% of maximum skull width) at the level of upper caudal angles of orbits. Parasphenoid alae are long and equal 40.3% length of cultriform process and articulate with dorsal (medial) ramus of pterygoid. Neopalatines are very thin and articulate with sphenethmoid dorsally and medial face of maxillary bone ventrally forming the anteroventral corner of orbit. Septomaxillae are small, horseshoe-shaped and articulate with prevomer dorsally. Prevomers are large and broadly separated from each other, the separation is greater posteriorly. Dentigerous processes are inconspicuous, more visible ventrally on left prevomer and slightly anterior to the palatines. Columella (or stapes) is large and well ossified. Nasals are thin, posteriorly expanded, anteriorly separated from each other, and articulated posteriorly with frontoparietals and neopalatines.

Maxillary arch bears many small and poorly defined teeth on maxillae and premaxillae.

Premaxillae are narrowly separated medially with their alary processes long, anteriorly oriented and completely separated from nasals. Each premaxilla articulates laterally with the maxilla. The latter becomes narrower posteriorly ending in an acuminate posterior tip that articulates with quadratojugals. The triradiate pterygoid bears a long, curved rostral ramus oriented anterolaterally toward maxilla. Caudal ramus of pterygoid is slightly longer and much wider than medial ramus; the former reaches quadratojugal at its medial face, while the latter reaches lateral edge of otoccipital and parasphenoid ala.

Quadratojugal is slender and articulates with ventral ramus of the T-shaped squamosal dorsally. Otic ramus (posterior ramus) of squamosal oriented posteromedially, flattened dorsoventrally and much longer than zygomatic ramus (anterior ramus); the latter is flattened laterally and 60.7% the length of the otic ramus. Mandible is narrow and edentate. Mentomeckelians are small, slightly broadened medially and laterally and separated medially from each other. Angulosplenial is long, arcuate and expanded at its posterior end; it articulates broadly by its anterolateral face with anteromedial face of dentaries. The only ossified portions of hyoid apparatus are two posteromedial processes, which are moderately expanded anteriorly and slightly expanded posteriorly. Both posteromedial processes present an anteroventral inclination and are moderately separated from each other at their anterior ends, the separation distance between them increases posteriorly.

Postcranium (Fig. 11). The specimen has eight non-imbricate presacral vertebrae. First presacral vertebra (cervical vertebra) is wider than posterior vertebrae and has no diapophyses. The cervical vertebra has a Type I cotylar arrangement. The cervical cotyles receive the occipital condyles of the cranium which are widely separated from each other. Foramen magnum is 25.5% the length of maximum skull width.

Presacral vertebrae II–VIII bear well-developed diapophyses. The transversal processes of presacral III are the longest and widest with their distal portion broadly expanded. In length, processes of presacral III are followed by presacrals IV and II, respectively. Transversal processes are widest in presacral III, followed by II (slightly expanded distally), and IV (no expansions). Transversal processes of presacral V–VIII are the smallest and similar in size (length and width). The transversal processes of presacrals II and III have a ventral orientation in relation to the transverse processes of presacrals IV to VIII; and transverse process of presacral II have a slightly anterodorsal orientation. Vertebra are characterized for having a holochordal centrum.

Sacrum bears one moderately expanded diapophysis. Transverse processes are oriented dorsolaterally with an angle of dorsal opening of ~157 degrees and articulates distally with anterior tips of ilia. Sacrum is articulated caudally with urostyle by a bicondylar articulation. Urostyle is long, thin, and slightly shorter than the presacral portion of the vertebral column; it bears a well-developed longitudinal ridge which is broadly expanded anteriorly (at the point of its articulation with sacrum) and gradually decreasing in height posteriorly. Urostyle lacks transverse processes. Sacrum-ilia articulation is not visible in the micro CT-scan. Ilia articulate posteromedially with each other and posteriorly with ischia.

Clavicles are long and slim, oriented anteromedially, with its rostral edge anteriorly concave and medial tips articulated between them. Coracoids are stout, with curved anterior edge (the curvature is more pronounced distally), and straight posterior edge; their medial tips are articulated. Glenoidal and sternal ends of coracoid are about equally expanded. Scapula is long with anterior edge slightly oriented anteromedially. Epicoracoids are visible. Sternum has no well-ossified elements. Omosternum is absent.

Manus and pes (Fig. 12). All phalanges are well ossified with a phalangeal formula for fingers and toes: 2-2-3-3 and 2-2-3-4-3, respectively. Finger length is I < II < IV < III, and that of toes is I < II < III < V < IV. Terminal phalanges of all toes and fingers are narrower distally with T-shaped tip, wider in fingers III and IV and in toes IV and V. It is difficult to distinguish the different elements of carpus and tarsus due to the low resolution of three-dimensional models.

**Distribution, natural history, and conservation status ( Fig. 1):** *Pristimantis anaiae* is known from the surroundings of Zarentza Community (elevation range is 1,350–1,419 m), Llanganates National Park, Canton Mera, Pastaza Province. Ecosystem type is Eastern Montane Forest (1,300–3,600 m.a.s.l.), as defined by Ron, Merino-Viteri & Ortiz (2021), which has 80% of remaining natural vegetation due to human activities. All specimens were collected at night over vegetation, perching on leaves or branches, 100 to 250 cm above ground, in primary and secondary forests.

Because of the lack of information on population size and geographic distribution, we recommend assigning *P. anaiae* to the Data Deficient IUCN Red List Category (based on IUCN, 2019 guidelines). The Llanganates region is largely unexplored (Navarrete, Venegas & Ron, 2016) and the occurrence of *P. anaiae* at other sites seems likely. Its presence in secondary forest suggest adaptability to anthropogenic habitat change. The land use and vegetation cover map (obtained from Ministerio del Ambiente del Ecuador, 2018) shows an area deforested by agricultural uses and human settlements corresponding to 10% of the total area within 5 km of the known localities. The collection localities are at a distance of 10.6 km from the main road E45 and 13.9 km from the town of Mera.

**Etymology:** The specific epithet is a patronym for Anaí Elizabeth Ortega Páez, the younger sister of the leading author. This species is named after her in gratitude for her love, support, and generosity. The species epithet is formed from the name “Anaí,” taken as a noun in the genitive case, with the Latin suffix “ae” (ICZN 31.1.2).

***Pristimantis glendae* sp. nov.**

urn:lsid:zoobank.org:act:D4D4D052-6015-4B02-8C9F-E336020922AA

**Figure 15:**
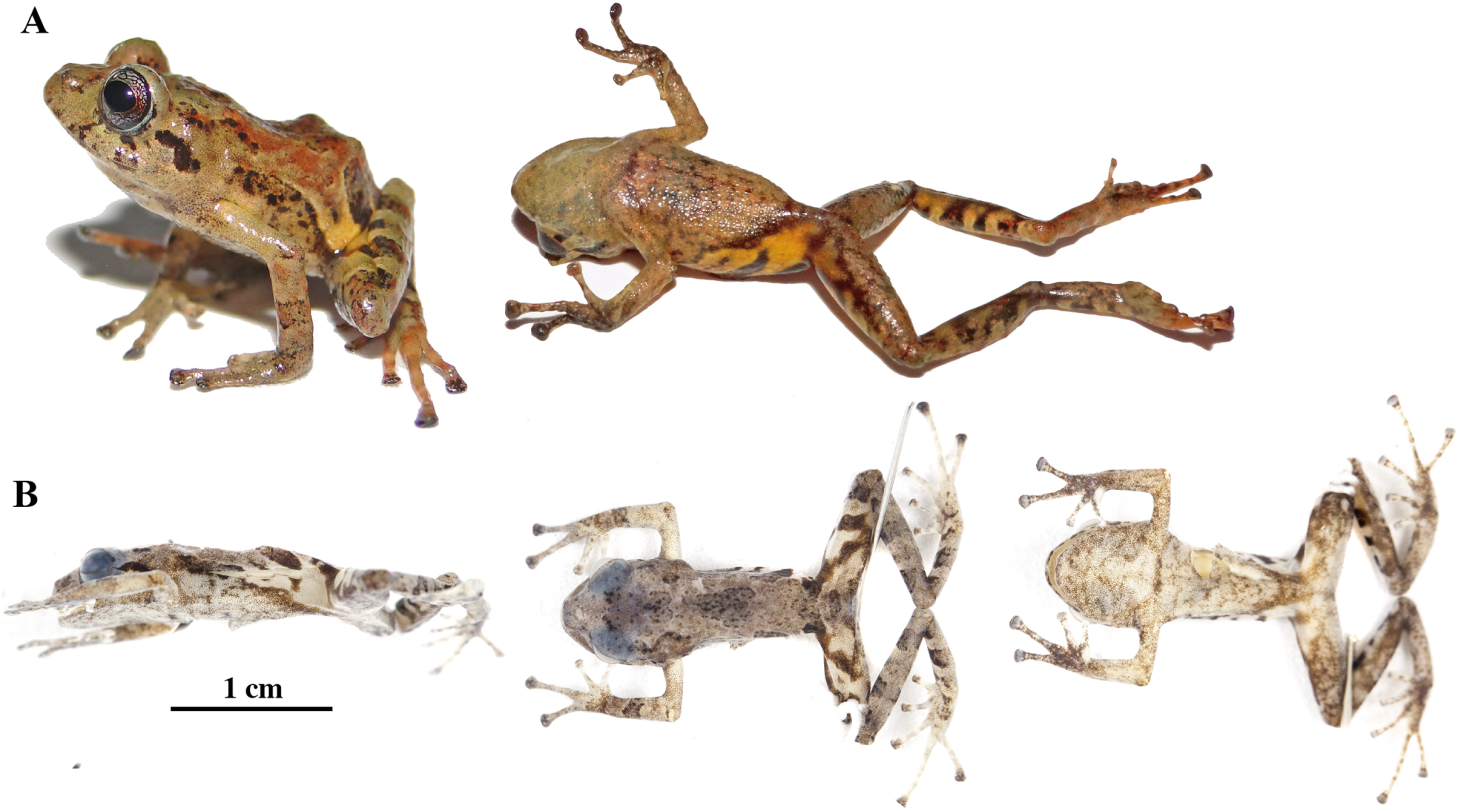
*Pristimantis glendae* sp. nov. Holotype, QCAZ 56437, adult male, SVL = 16.60 mm. (A) Photographs of alive individual in lateral and ventral view. (B) Photographs of preserved individual in lateral, dorsal and ventral view.

**Figure 16:**
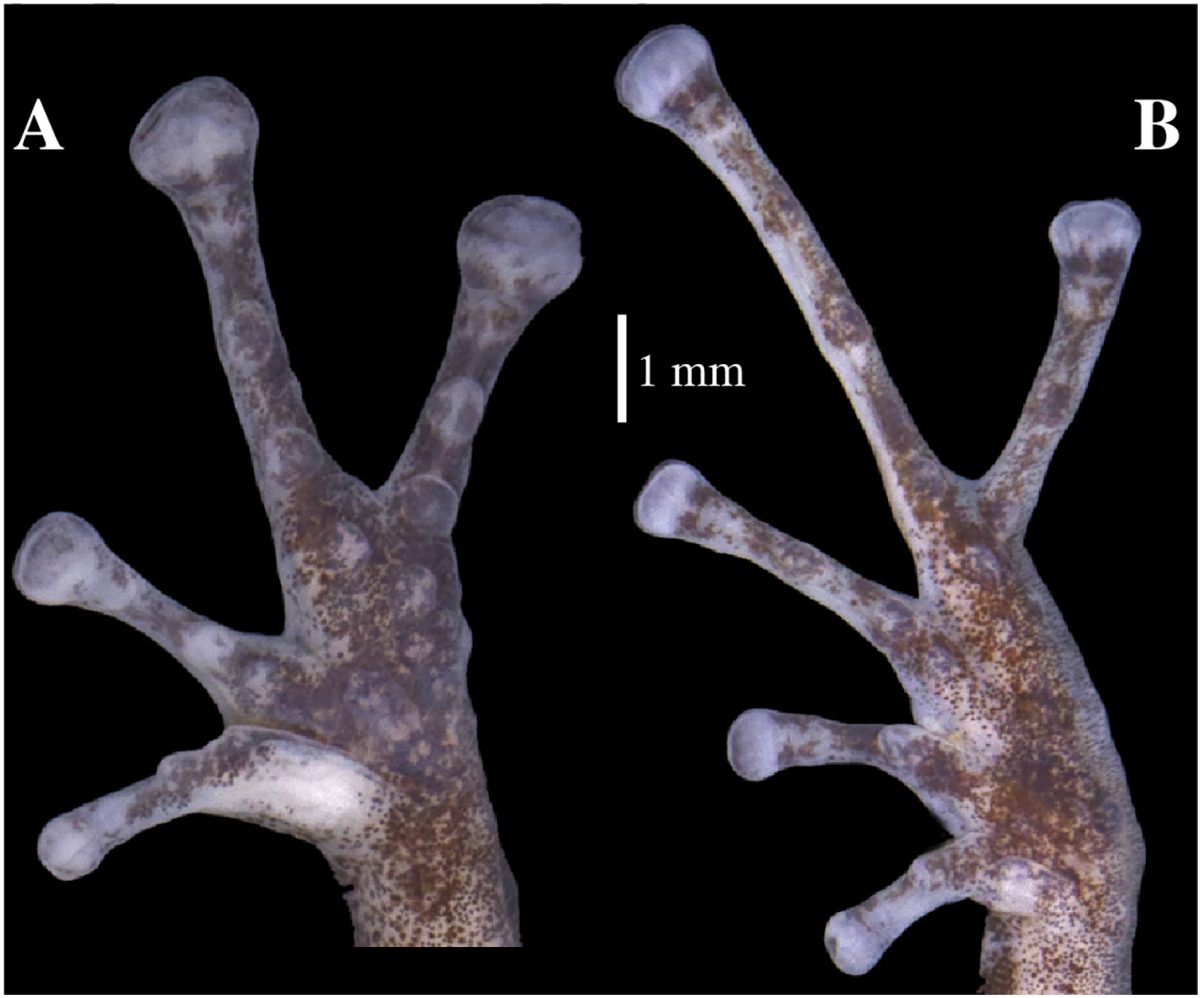
(A) Palmar and (B) plantar surfaces of *Pristimantis glendae* sp. nov. Photographs of left hand and foot of the holotype QCAZ 56437.

**Holotype (Figs. 15, 16):** QCAZ 56437 (field number JBM 525), an adult male from Ecuador, Morona Santiago Province, Canton Morona, Parish Sinaí, Sangay National Park, Paso Estrecho, between Río Volcán and Río Sangay, near a temporary camp, eastern base of the Sangay volcano, (2.0569° S; 78.2772° W), 2,191 m, collected by J. Brito on 25 August 2013.

**Figure 17:**
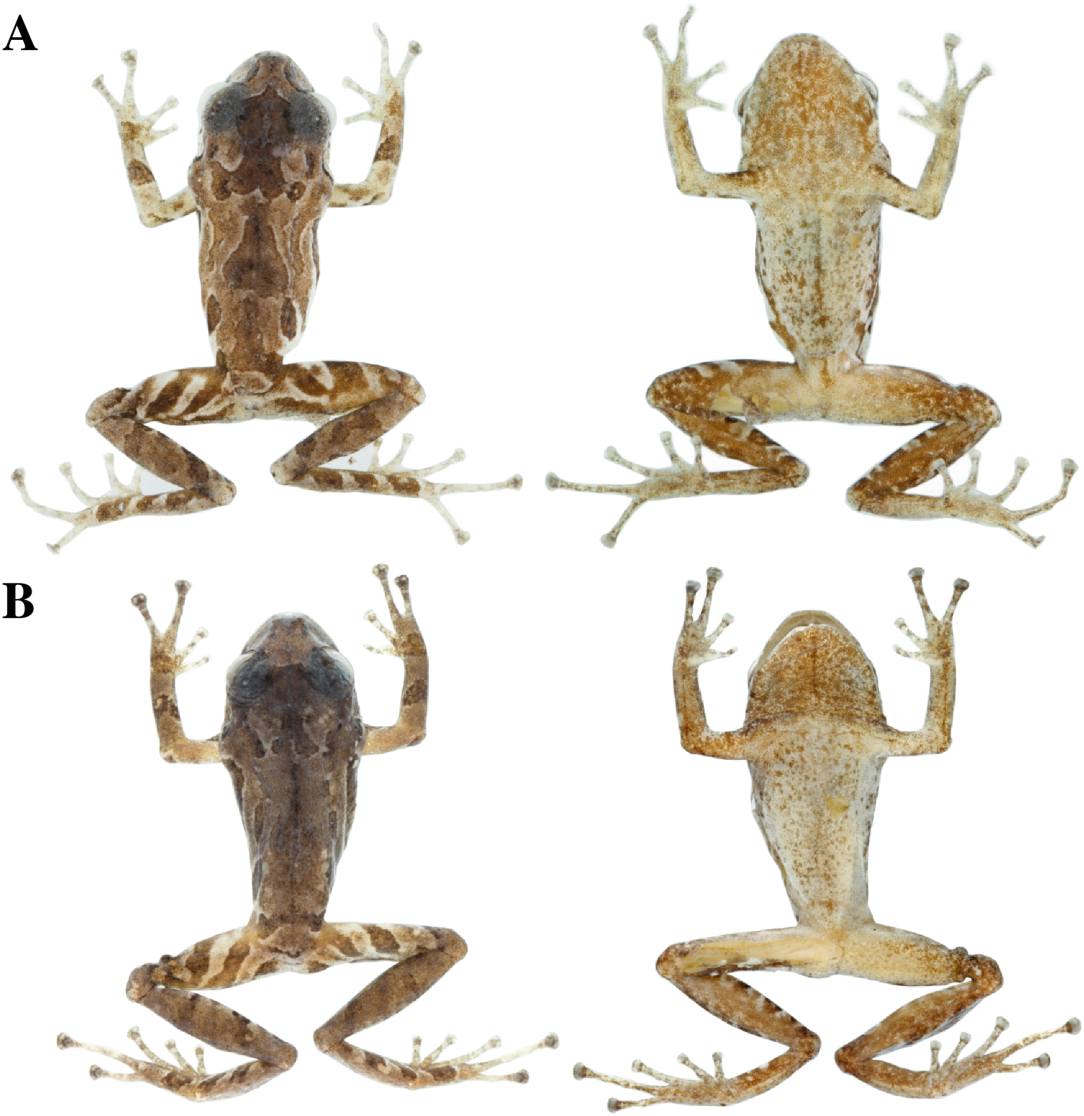
Color variation in preserved individuals of *Pristimantis glendae* sp. nov. (A) QCAZ 45784, paratype, adult male, SVL = 19.09 mm. (B) QCAZ 45953, paratype, adult male, SVL = 18.29 mm. Lateral view on the left, dorsal view in the center and ventral view on the right.

**Paratypes (*n* = 4; Fig. 17):** All from Ecuador, Pastaza Province, Canton Mera, Llanganates National Park, Community Ankaku, Buffer zone of Llanganates National Park, Challuwa Yacu River, QCAZ 45784, QCAZ 45793, QCAZ 45832, QCAZ 45953 adult males, 1.2762° S, 78.0725° W, 2,266 m. Collected by Elicio Tapia and Silvia Aldás on October 21, 2009.

**Common name:** English: Glenda Rain Frog. Spanish: Cutín de Glenda.

**Diagnosis (Figs. 15–17):** We assign the new species to the genus *Pristimantis* based on the phylogeny (Fig. 5). A species of *Pristimantis* characterized by: (1) skin of dorsum shagreen, skin on venter areolate sometimes bearing low and rounded tubercles; (2) discoidal fold absent; (3) dorsolateral folds absent; (4) tympanic membrane present, tympanic annulus present (on average 32.6% of eye diameter); (5) one or two small and rounded postrictal tubercles; (6) supratympanic folds present; (7) snout short and rounded in dorsal and lateral view; (8) upper eyelid with few low tubercles; (9) cranial crests absent; (10) vocal slits and nuptial pads absent; (11) Finger I shorter than Finger II, discs of digits expanded and truncate; (12) fingers with narrow lateral fringes, hyperdistal subarticular tubercles of fingers rounded in ventral and lateral views; (13) ulnar tubercle small and rounded; (14) heel without tubercles; (15) toes with broad lateral fringes, basal webbing between toes present, all toes with elongated hyperdistal tubercles; (16) Toe V much longer than Toe III, Toe condition C (Toe III surpasses the distal border of the distal subarticular tubercle of Toe V and reaches the proximal border of the penultimate tubercle of Toe IV; Toe V reaches the proximal border of the distal tubercle of toe IV); (17) dorsum orange, yellow or yellowish brown with scapular blotches warm sepia with dark bars with thin light cream-colored edges on sacral areas; belly whitish cream full of small black flecks or mottled with a medial black line; flanks pale orange bearing a short warm sepia longitudinal stripe; groins and anterior surfaces of thighs orange-yellow or golden; iris greenish white to white with dark reticulations; (18) SVL in females unknown; SVL in adults males 16.60–19.93 mm (Tables 4, S2).

**Comparison with other species:** *Pristimantis glendae* sp. nov., differs from other *Pristimantis* of the eastern foothills of the Andes and lower Amazon of Ecuador by lacking discoidal fold, and by having distinctive orange-yellow or golden coloration in the groin, slightly greenish white to white iris with dark reticulations, and light blue sclera in life. The only species within the geographical range with which *Pristimantis glendae* sp. nov. could be confused is *P. churuwiai* Brito, Batallas & Yánez-Muñoz, 2017 by the presence of low and small ulnar tubercles, broad and truncated finger discs and outer border of the tarsus with small, rounded tubercles. However, *Pristimantis glendae* sp. nov. is distinguished by the absence of dorsolateral folds (present in *P. churuwiai*), absent internarinal subconical tubercle (present in *P. churuwiai*), presence of basal webbing in toes (absent in in *P. churuwiai*), distinctive orange-yellow or golden spots on groin surfaces (yellowish cream in *P. churuwiai*).

Other species with which *P. glendae* sp. nov. could be confused are *P. nigrogriseus* (Lynch & Duellman, 1980) from the eastern slope of the Andes by the absence of dorsolateral folds, by the areolate belly and broadly expanded finger discs, and *P. miktos* Ortega-Andrade & Venegas, 2014 from the Amazon lowlands by the absence of vocal slits and presence of enlarged and rounded palmar supernumerary tubercles. *Pristimantis glendae* sp. nov., has orange-yellow or golden in the groin, and greenish white to white iris with dark reticulations in life (groin and surface of thighs black with bright yellow spots, and red iris without reticulations in *P. nigrogriseus*). *Pristimantis glendae* sp. nov., does not present dermal ridges in the scapular region (W or V-shaped mark present in *P. miktos*), upper eyelid with a small, rounded tubercles (absent in *P. miktos*), presence of lateral fringes in fingers and toes (absence of lateral fringes in *P. miktos*).

**Description of the holotype (Figs. 15, 16):** Live and preserved coloration is shown in Fig. 15. Adult male (QCAZ 56437). Measurements (in mm): SVL 16.60; tibia length 8.60; foot length 8.61; head length 6.55; head width 6.10; eye diameter 2.30; tympanum diameter 0.75; interorbital distance 1.76; upper eyelid width 1.64; internarial distance 1.35; eye-nostril distance 1.60.

Head longer than wide, wider than body, snout rounded in dorsal view and in profile; canthus rostralis concave in lateral view; loreal region slightly concave; cranial crests absent, upper eyelid with few low tubercles; tympanic annulus present; tympanic membrane present; one or two small rounded postrictal tubercles. Supratympanic folds present, ill-defined. Dentigerous processes of vomers present, oblique, broadly separated, posteromedial to choanae; each vomer bearing several inconspicuous small teeth; vocal slits and nuptial pads absent.

Skin on dorsum shagreen; discoidal and dorsolateral folds absent; belly areolate with some low and rounded tubercles; skin in upper cloacal region areolate. Ulnar tubercle present, small, low and rounded; small palmar surface; heart-shaped palmar tubercle, slightly elevated, thenar tubercle medium and oval; supernumerary tubercles enlarged, slightly raised, and scattered; subarticular tubercles well defined, round in ventral and lateral view, hyperdistal subarticular tubercles of fingers rounded, hyperdistal subarticular tubercle of digit III is eloganted; all rounded in lateral view; supernumerary tubercles at base of fingers present, enlarged, rounded; narrow lateral fringes on fingers; Finger I shorter than Finger II; discs on Fingers I and Finger II expanded, discs on Fingers III and IV broadly expanded and truncate; pads on all fingers well defined and surrounded by circumferential grooves (Fig. 16).

Hindlimbs slender; upper surfaces of hindlimbs irregularly areolate; posterior surfaces of thighs areolate, ventral surfaces of thighs smooth; heel without tubercles; outer surface of tarsus bearing inconspicuous small tubercles; tarsal fold present; inner metatarsal tubercle elongate (0.73 mm), elliptical, rounded in lateral view, two times longer than rounded, well-defined outer metatarsal tubercle (0.39 mm); plantar surface with some ill-defined supernumerary tubercles; subarticular tubercles well defined, prominent, rounded in ventral and lateral views; all toes with elongated hyperdistal tubercles rounded in lateral view; toes with broad lateral fringes; basal webbing between toes present; discs nearly as large as those on fingers, expanded in toes II and II, broadly expanded in toes III, IV, and V; all discs have pads surrounded by well-defined circumferential grooves; relative lengths of toes I < II < III < V < IV; Toe V much longer than Toe III (Toe III reaches the proximal border of the penultimate subarticular tubercle of Toe IV and surpasses the distal border of the distal tubercle of Toe V; Toe V reaches the proximal border of the distal subarticular tubercle of toe IV) (Fig. 16).

**Variation (Fig. 17):** In this section, traits refer to preserved individuals; we only mention character states not observed in the holotype. Low and rounded tubercles may be absent in belly (*e.g*. QCAZ 45793, QCAZ 45953). Ulnar tubercles not visible in QCAZ 45793; hyperdistal tubercles of fingers might be prominent (*e.g*. QCAZ 45793); supernumerary plantar tubercles may be well-defined (*e.g*. QCAZ 45784). Supratympanic fold well-defined in QCAZ 45832.

**Distribution, natural history, and conservation status (Fig. 1)**: *Pristimantis glendae* is known from the surroundings of the Volcan River (between the Volcan River and the Sangay River), in the eastern foothills of Sangay volcano, Sangay National Park and from the surroundings of Ankaku Community, Llanganates National Park (elevation range is 2,191–2,266 m). Ecosystem type is Eastern Montane Forest (1,300–3,600 m asl), as defined by Ron, Merino-Viteri & Ortiz (2021). *Pristimantis glendae* is known from cloud forests characterized by trees that reach 25 m in height with abundant moss, orchids, ferns, and bromeliads. The specimens were collected in flat ground primary forest with abundant bamboo (*Chusquea* sp.) and plants of the family Araceae. All specimens were collected at night between 19:30 and 21:00 perching about 1.50 m above the ground. *Pristimantis glendae* inhabits in sympatry with *P. kunam* sp. nov. and *P. bicantus*. Data Deficient seems an appropriate category for *P. glendae* because almost nothing is known about this frog.

**Etymology:** This species is named in honor of Glenda Marisol Pozo Zamora (Ecuador, b. 25 Jul 1988), an outstanding ornithologist, in recognition of her collections and explorations that have allowed us to learn about the diversity of birds in several remote areas of Ecuador. The species epithet is formed by the name “Glenda” taken as a noun in genitive case, with the Latin suffix “e” (ICZN 31.1.2).

***Pristimantis kunam* sp. nov.**

urn:lsid:zoobank.org:act:7DD2C62D-E728-42CD-BDD5-F526EDD8D4FD

**Figure 18:**
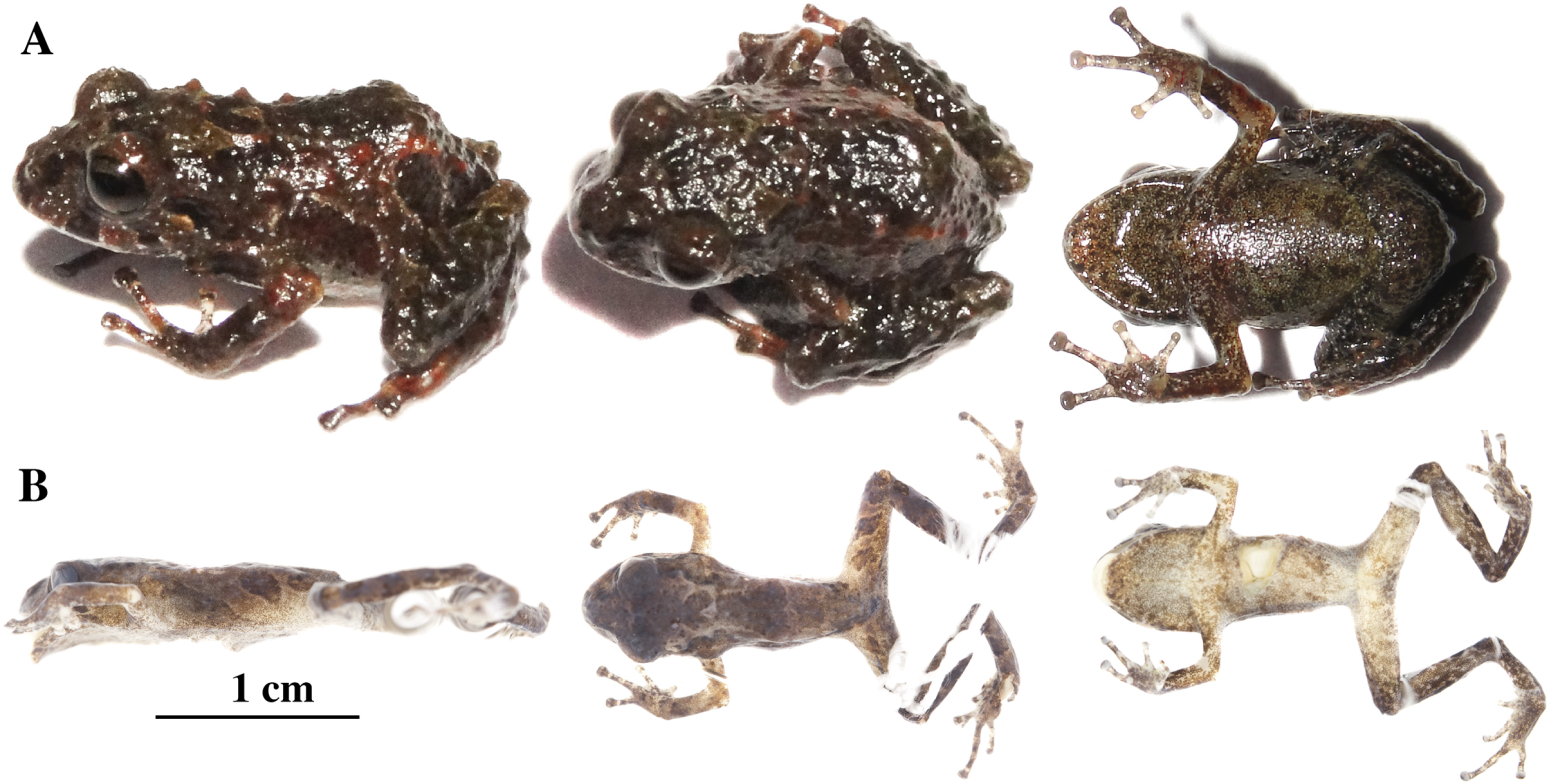
*Pristimantis kunam* sp. nov. Holotype, QCAZ 56438, adult male, SVL = 14.66 mm. (A) Photographs of alive individual in lateral, dorsal and ventral view. (B) Photographs of preserved individual in lateral, dorsal and ventral view.

**Figure 19:**
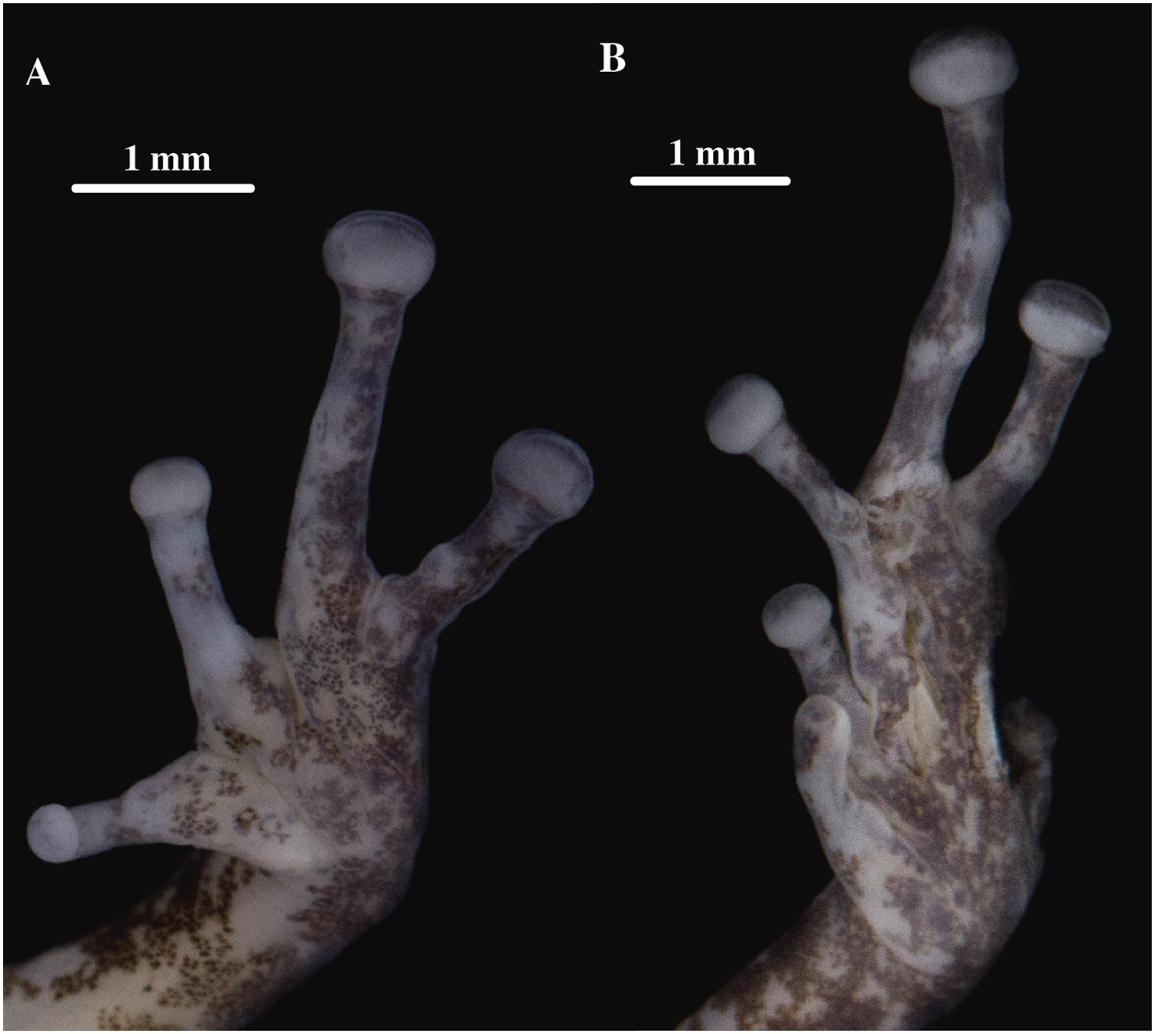
(A) Palmar and (B) plantar surfaces of *Pristimantis kunam* sp. nov. Photographs of left hand and foot of the holotype QCAZ 56438.

**Holotype (Figs. 18, 19):** QCAZ 56438 (field no. JBM526), adult male from Ecuador, Morona Santiago Province, Canton Morona, Parish Sinaí, Sangay National Park, Paso Estrecho (2.0569° S; 78.2772° W), 2,191 m, collected by Jorge Brito on August 25, 2013.

**Common name:** English: Kunam Rain Frog. Spanish: Cutín de Kunam.

**Diagnosis (Figs. 18, 19):** We assign the new species to the genus *Pristimantis* based on the phylogeny (Fig. 5). A species of *Pristimantis* characterized by: (1) skin of the dorsum tuberculate with some large and conical tubercles forming a row on dorsolateral lines, skin on venter areolate with scattered tubercles; discoidal fold absent; dorsolateral folds absent; (2) tympanic membrane absent, tympanic annulus present; postrictal tubercle present; supratympanic folds present; (3) snout short and rounded in dorsal and lateral view; (4) upper eyelid with one conical tubercle and few lower tubercles; cranial crests absent; (5) vocal slits and nuptial pads absent; (6) Finger I shorter than Finger II, discs of digits expanded, slightly truncate; (7) fingers with narrow lateral fringes, all fingers with hyperdistal tubercles elongated in ventral and rounded in lateral view; (8) three large, conical ulnar tubercles; (9) heel bearing several tubercles; (10) toes with narrow lateral fringes, basal webbing absent, all toes with hyperdistal tubercles elongated in ventral view and rounded in lateral view; (11) Toe V much longer than Toe III, Toe condition C (Toe III surpasses the distal border of the distal subarticular tubercle of Toe V and reaches the proximal border of the penultimate tubercle of Toe IV; Toe V reaches the middle of the distal tubercle of toe IV); (12) dorsum dark brown with a row of large orange conical tubercles extending along the dorsolateral line; large sacral dark round areas with thin clear borders; ventral surfaces dark greenish with dark flecks; dark bronze iris with fine black reticulation; (13) SVL in females unknown; SVL in adult male 14.66 mm (*n* = 1) (Tables 4, S2).

**Comparison with other species:** *Pristimantis kunam* sp. nov. resembles *P. anaiae* sp. nov. by the presence of large dark round areas with thin clear borders on sacral regions and tubercles on the upper eyelids. It differs from *P. anaiae* sp. nov. by having a row of large conical tubercles extending along the dorsolateral line to the level of the groin (absent in *P. anaiae*), for having narrower discs, and by the absence of basal webbing (present in *P. anaiae*). *Pristimantis kunam* sp. nov. differs from similar spiny species such as *P. roni, P. yanezi, P. llanganati*, and *P. katoptroides* by the absence of tympanic membrane and the presence of the dorsolateral row of large orange conical tubercles and large sacral dark round areas with thin clear borders (none of these species has the characteristic color pattern of *P. kunam* and all of them have tympanic membrane). *Pristimantis kunam* sp. nov., also resembles *P. verecundus* and *P. mutabilis* by the presence of large sacral dark round areas with thin clear borders. It differs from them by the absence of tympanic membrane and vocal slits (both present in *P. mutabilis*) and by the presence of a dorsolateral row of orange tubercles and dark brown groins (dorsolateral row of tubercles absent and red groins in *P. verecundus*).

**Description of the holotype (Figs. 18, 19):** Live and preserved coloration is shown in Fig. 17. Adult male (QCAZ 56438). Measurements (in mm): SVL 14.66; tibia length 7.32; foot length 6.15; head length 4.92; head width 5.05; eye diameter 2.15; tympanum diameter 0.46; interorbital distance 1.47; upper eyelid width 1.82; internarial distance 1.35; eye-nostril distance 1.28; testes 10.23% of SVL.

Head wider than long, slightly wider than body, snout rounded in dorsal view and in profile; canthus rostralis straight in lateral view; loreal region slightly concave; cranial crests absent, upper eyelid bearing one big conspicuous conical tubercle surrounded by few indistinct smaller tubercles; tympanic annulus distinct beneath the skin, more conspicuous on its rostral and ventral portions; tympanic membrane absent; several rounded, low, postrictal tubercles. Supratympanic fold present, inconspicuous and dark brown. Dentigerous processes of vomers present, oblique, separated, posteromedial to choanae; each vomer bearing several inconspicuous small teeth; vocal slits and nuptial pads absent.

Skin on dorsum tuberculate with some large and conical tubercles forming a row on dorsolateral lines, skin on flanks tuberculated with some tubercles larger than others, dorsolateral and discoidal folds absent; skin on throat, chest and belly areolate with some scattered white, low and rounded tubercles; ventral surfaces of thighs areolate; skin in upper cloacal region areolate. Three ulnar tubercles present, conspicuous big and conical; palmar tubercles low, outer palmar tubercle difficult to characterize due to the preservation of the individual; however, it is round, small, almost flat, and shorter than oval thenar tubercle; subarticular tubercles well-defined, pronounced round in ventral and lateral view; hyperdistal tubercles elongated in ventral view and rounded in lateral view; no supernumerary tubercles; lateral fringes on fingers narrow; Finger I shorter than Finger II; discs on Fingers I and Finger II slightly expanded and rounded, discs on Fingers III and IV expanded and slightly truncate; pads on all fingers well defined and surrounded by circumferential grooves (Fig. 19).

Hindlimbs slender; upper surfaces of hindlimbs tuberculated bearing conical tubercles; posterior and ventral surfaces of thighs areolate; heel bearing several low tubercles; outer surface of tarsus bearing large conical tubercles; tarsal fold present but inconspicuous; well-defined inner metatarsal tubercle small, elliptical in ventral view and rounded in lateral view, outer metatarsal tubercle difficult to characterize due to the preservation of the individual; plantar surface with several small and rounded supernumerary tubercles; subarticular tubercles well-defined, slightly prominent and rounded; hyperdistal tubercles elongated in ventral view and rounded in lateral view; toes with narrow lateral fringes; basal webbing between toes absent; discs nearly as large as those on fingers, expanded in all toes specially on Toes III, IV and V; all discs have pads surrounded by well-defined circumferential grooves; relative lengths of toes I < II < III < V < IV; Toe V much longer than Toe III (Toe III surpasses the distal border of the distal subarticular tubercle of Toe V and reaches the proximal border of the penultimate tubercle of Toe IV; Toe V reaches the middle of the distal tubercle of toe IV) (Fig. 19).

**Distribution, natural history, and conservation status (Fig. 1)**: *Pristimantis kunam* sp. nov. is known from a single locality on the eastern foothills of Sangay volcano. Ecosystem type is Eastern Montane Forest (1,300–3,600 m.a.s.l.), as defined by Ron, Merino-Viteri & Ortiz (2021). The single known individual was collected at night, on primary forest, perching on a fern leaf 130 cm above the ground.

Because of the lack of information on population size and geographic distribution, we recommend assigning *P. kunam* sp. nov. to the Data Deficient IUCN Red List Category (based on IUCN, 2019 guidelines). The eastern Sangay region is largely unexplored, and the occurrence of *P. kunam* sp. nov. at other sites seems likely. Its presence, so far identified only in primary forest, suggests low adaptability to anthropogenic habitat change. It is important to mention that Sangay volcano has been erupting frequently since May 17, 2019. Eruptions are explosive and with pyroclastic flows towards the southeastern flanks, with ash columns exceeding 10 km in height. The strong eruptions may have affected the type locality of the species, the current status of its population is unknown. Because it is a locality of difficult access it shows low deforestation, 0.12% within a 5 km radius (based on a deforestation map from Ministerio del Ambiente del Ecuador, 2018).

**Etymology:** The specific epithet is a patronym for Kunam Eloy Nusirquia Sandu, field technician of the Museum of Zoology, Pontificia Universidad Católica del Ecuador. He was part of the expedition when the species was found; at the time he was a park ranger of Sangay National Park. Kunam is a Native American that belongs to a Shuar community in southeastern Ecuador. This species is dedicated to him in recognition of his outstanding capacity to find amphibians and reptiles in the field, which resulted in the discovery of numerous new species, as part of the Arca de Noé initiative.

***Pristimantis resistencia* sp. nov.**

urn:lsid:zoobank.org:act:9A710AB8-CE6C-4FC7-A0AC-7A516ACABC92

**Figure 20:**
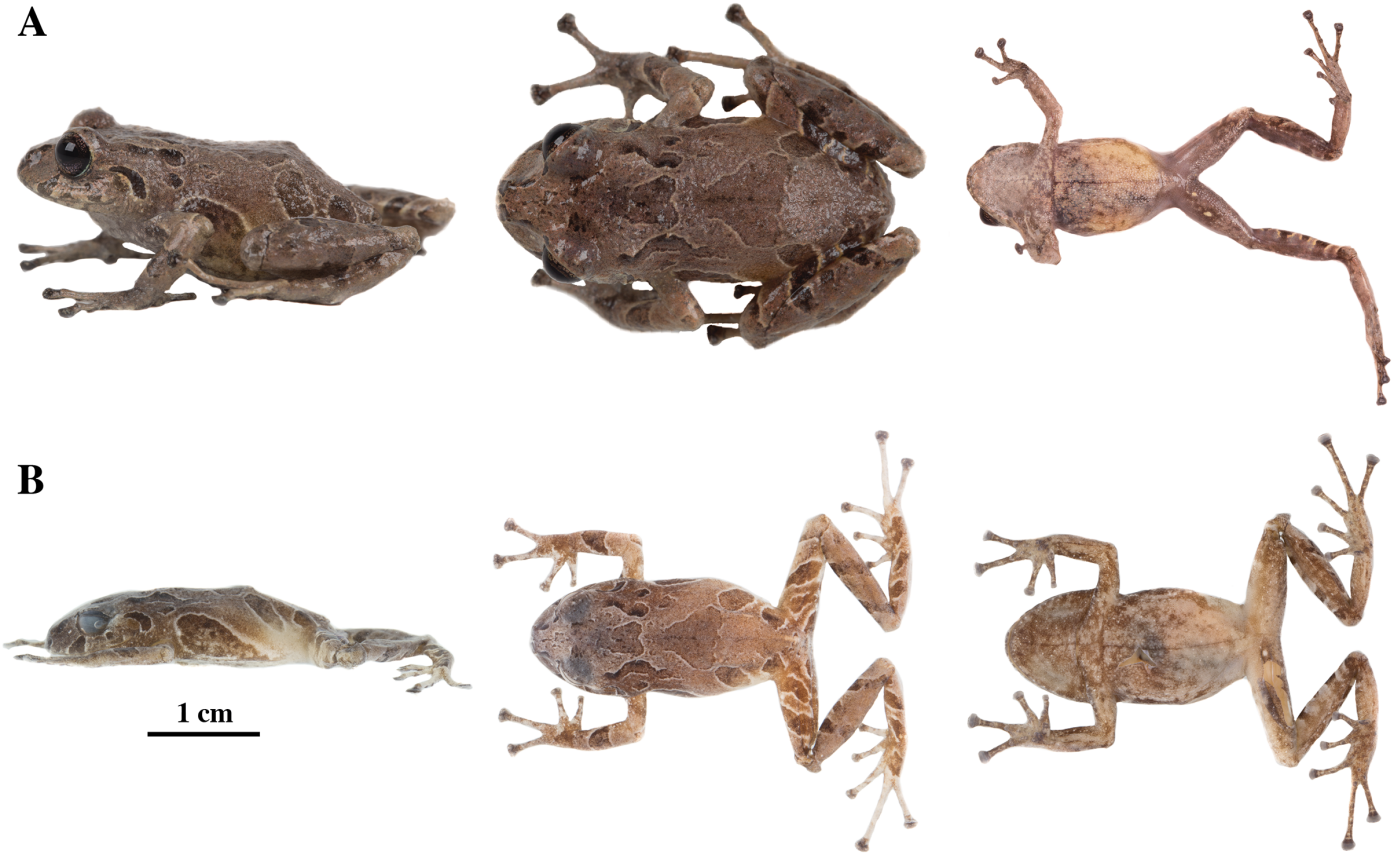
*Pristimantis resistencia* sp. nov. Holotype, QCAZ 66519, adult female, SVL = 24.59 mm. (A) Photographs of alive individual in lateral, dorsal and ventral view. (B) Photographs of preserved individual in lateral, dorsal and ventral view. Scales are given for dorsal view photographs of preserved individuals only.

**Figure 21:**
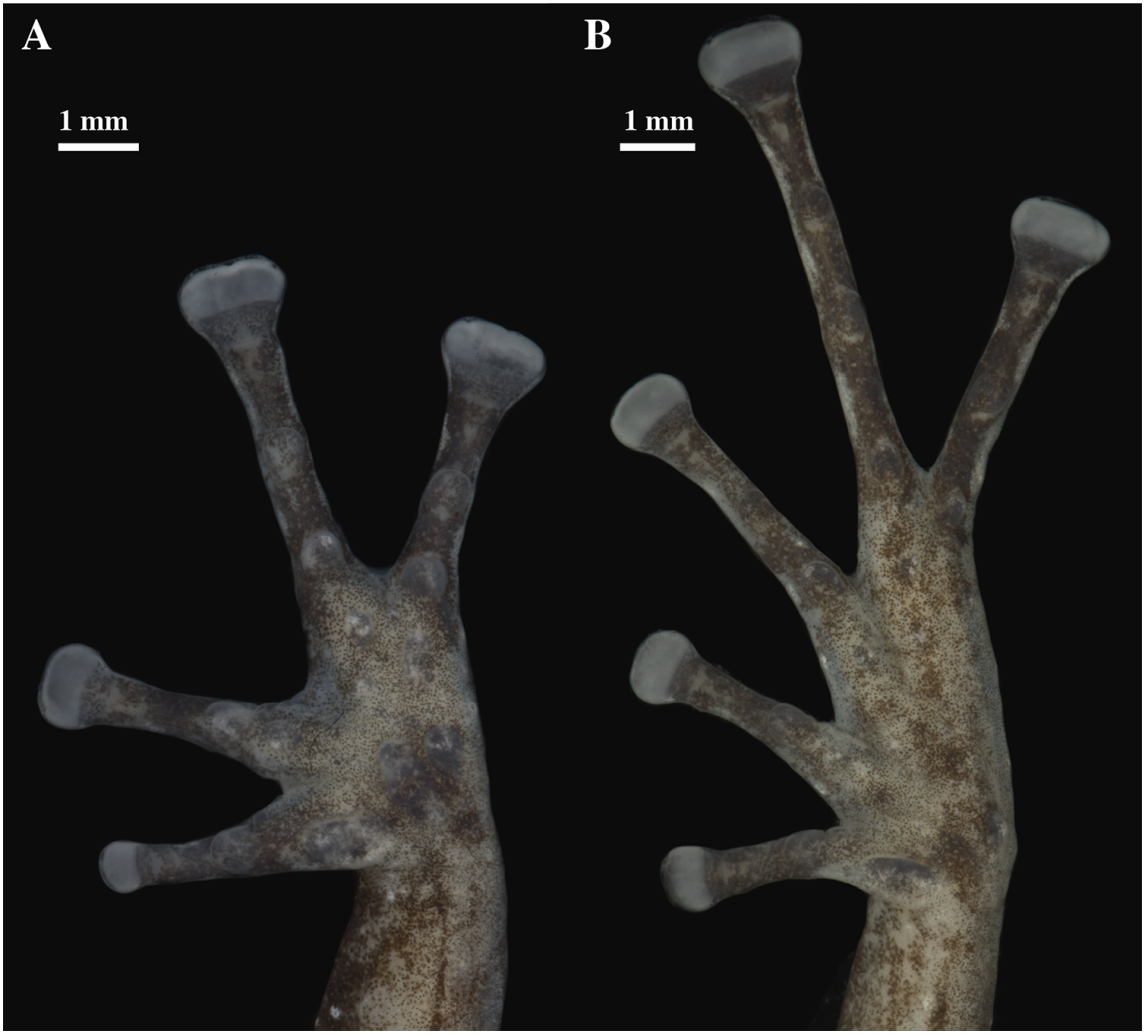
(A) Palmar and (B) plantar surfaces of *Pristimantis resistencia* sp. nov. Photographs of left hand and foot of the holotype QCAZ 66519.

**Holotype (Figs. 20, 21):** QCAZ 66519 (field no. SC-PUCE 52789), adult female from Ecuador, Pastaza Province, canton Mera, Parish Mera, San Rafael, Ankaku Community Reserve, buffer zone of Llanganates National Park (1.2801° S; 78.0822° W), 2,451 m, collected by Diego Almeida, Santiago Guamán, Darwin Núñez, María José Navarrete, Verónica Andrade, Ángel Alvarado and Fernando Alvarado on January 28, 2017.

**Figure 22:**
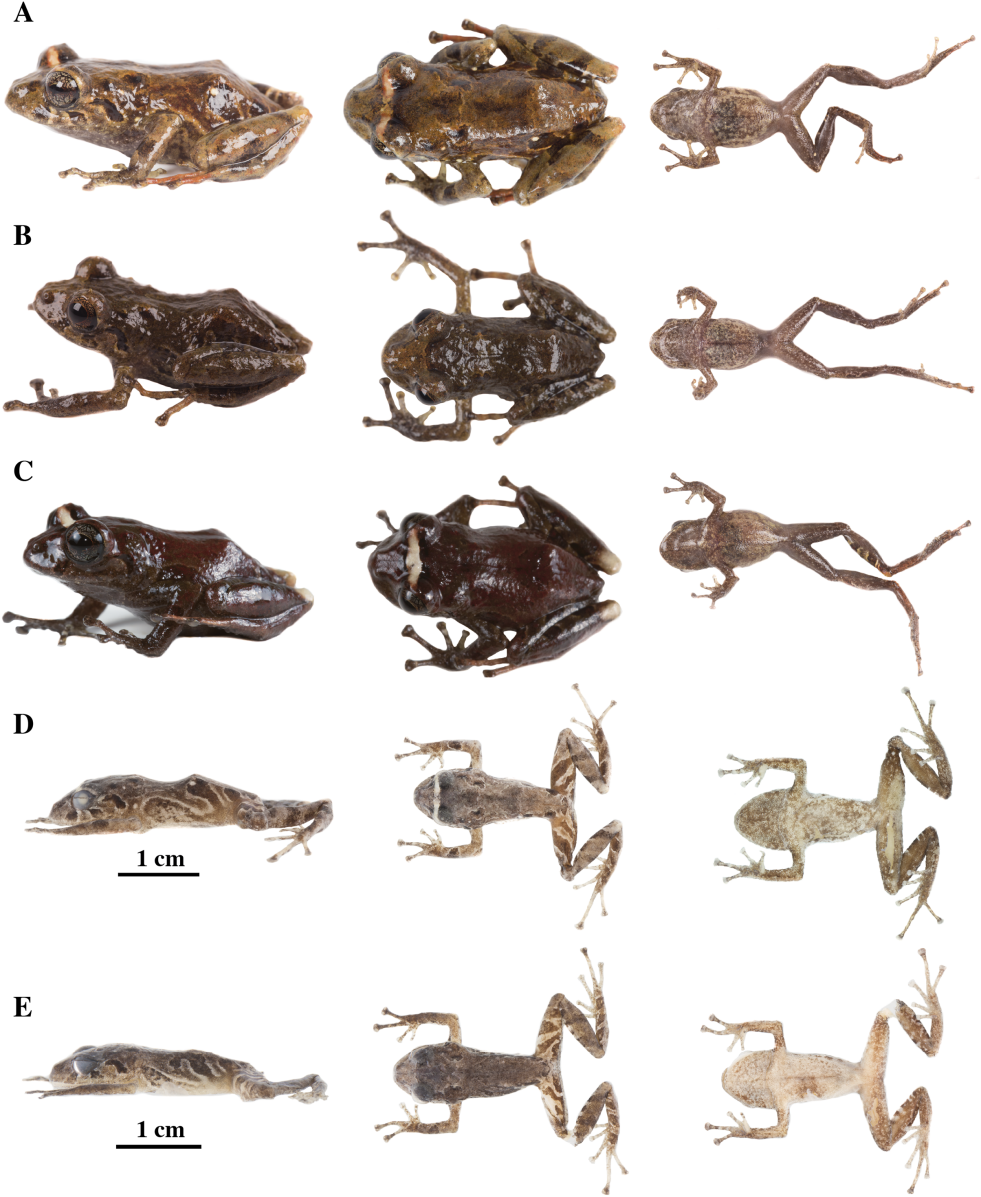
Color variation in alive and preserved individuals of *Pristimantis resistencia* sp. nov. (A, D) QCAZ 66467, paratype, adult male, SVL = 19.75 mm. (B, E) QCAZ 66523, paratype, adult male, SVL = 19.40 mm. (C) QCAZ 66372, paratype, adult male, SVL = 17.56 mm. Lateral view on the left, dorsal view in the center and ventral view on the right. Scales are given for dorsal view photographs of preserved individuals only.

**Paratypes (*n* = 3; Fig. 22):** Adult males from Ecuador, Pastaza Province, canton Mera, Parish Mera, San Rafael, Ankaku Community Reserve, buffer zone of Llanganates National Park. QCAZ 66372, 1.2761° S; 78.0729° W, 2,281 m; QCAZ 66467, 1.2768° S; 78.0764° W, 2,322 m; and QCAZ 66523 1.2793° S; 78.0782° W, 2,327 m, collected by Diego Almeida, Santiago Guamán, Darwin Núñez, María José Navarrete, Verónica Andrade, Ángel Alvarado and Fernando Alvarado on January 26–28, 2017.

**Common name:** English: Resistance Rain Frog. Spanish: Cutín de la Resistencia.

**Diagnosis (Figs. 20–22):** We assign the new species to the genus *Pristimantis* based on the phylogeny (Fig. 5). A species of *Pristimantis* characterized by: (1) skin of dorsum shagreen, skin on flanks shagreen to areolate; skin of venter areolate; (2) discoidal fold present but might be not visible in some individuals; (3) dorsolateral folds absent; (4) tympanic membrane and tympanic annulus present; (5) supratympanic fold present; (6) several rounded and low postrictal tubercles; (7) snout short and rounded in dorsal and lateral view; (8) upper eyelid with one subconical tubercle surrounded by few indistinct smaller and rounded tubercles; (9) cranial crests absent; (10) vocal slits and nuptial pads absent; (11) Finger I shorter than Finger II, discs of digits expanded, truncate; (12) fingers with broad lateral fringes, supernumerary tubercles present, all fingers bearing elongated hyperdistal tubercles; (13) two ulnar tubercles present; (14) heel bearing several low tubercles; (15) toes with broad lateral fringes, basal webbing present, supernumerary tubercles present, all toes bearing hyperdistal tubercles; (16) Toe V much longer than Toe III, Toe condition C (Toe III surpasses the distal border of the distal tubercle of Toe V and reaches the middle of the penultimate tubercle of Toe IV; Toe V surpasses the distal border of the distal tubercle of Toe IV); (17) dorsum light brown, dark reddish brown or dark greenish brown with two dorsal triangles, inguinal and sacral markings of dark brown color and pale edges, ventral surfaces brownish cream with dark brown patches, copper iris with thick black reticulation; (18) SVL in the only known female 24.59 (*n* = 1); SVL in adult males 18.90 mm (*n* = 3) (Tables 4, S2).

**Comparison with other species:** *Pristimantis resistencia* sp. nov. resembles its closest relatives *P. anaiae* sp. nov., *P. glendae* sp. nov., and *P. kunam* sp. nov. by having sacral markings of dark brown color and pale edges, presence of tympanic annulus and tuberculated upper eyelids. It differs from *P. kunam* sp. nov. by having less tuberculation in the dorsum (large conical tubercles on the dorsum of *P. kunam* sp. nov.), presence of tympanic membrane (absent in *P. kunam* sp. nov.), fingers with broad lateral fringes (narrow in *P. kunam* sp. nov.) and by the presence of basal webbing on toes (absent in *P. kunam*). *Pristimantis resistencia* differs from *P. glendae* sp. nov. by the presence of broad lateral fringes in fingers (narrow in *P. glendae* sp. nov.), by its brown groins (orange-yellow or golden groins in *P. glendae* sp. nov.) and by its copper iris with thick black reticulations (greenish white to white iris with dark reticulations in *P. glendae* sp. nov.). *Pristimantis resistencia* sp. nov. differs from *P. anaiae* sp. nov. by the absence of ventral tuberculation and the copper iris with thick black reticulation (ventral surfaces bearing black or dark brown tubercles, iris bronze with a wide copper medial band and black reticulations in *P. anaiae* sp. nov.). *Pristimantis resistencia* differs from similar spiny species such as *P. roni, P. yanezi, P. llanganati*, and *P. katoptroides* by the presence of basal webbing between toes and its characteristic dark areas with pale borders on dorsum and flanks (none of these species has the characteristic color pattern of *P. resistencia* sp. nov. and none of them has basal toe webbing). *Pristimantis resistencia* sp. nov. also resembles *P. verecundus* and *P. mutabilis* by the presence of large sacral dark round areas with thin clear borders *Pristimantis mutabilis* differs by having vocal slits (absent in *P. resistencia* sp. nov.) *Pristimantis verecundus* can be easily distinguished by its red groins (groins are dark brown in *P. resistencia* sp. nov.)

**Description of the holotype (Figs. 20, 21):** Live and preserved coloration is shown in Fig. 19. Adult female (QCAZ 66519). Measurements (in mm): SVL 24.59; tibia length 12.29; foot length 11.95; head length 8.31; head width 10.02; eye diameter 3.05; tympanum diameter 1.22; interorbital distance 2.78; upper eyelid width 2.46; internarial distance 1.98; eye-nostril distance 2.32.

Head wider than long, slightly wider than body, snout rounded in dorsal view and in profile; canthus rostralis slightly convex in lateral view; loreal region concave; cranial crests absent, upper eyelid bearing one small conspicuous subconical tubercle surrounded by few indistinct smaller and rounded tubercles; tympanic annulus distinct beneath the skin, more conspicuous on its ventral portion; tympanic membrane present; several rounded, low, postrictal tubercles. Supratympanic fold present, small, dark brown. Dentigerous processes of vomers present, oblique, separated, positioned posterior to the level of choanae; each vomer bearing several inconspicuous small teeth; vocal slits and nuptial pads absent.

Skin on dorsum shagreen, flanks and ventral surfaces areolate, dorsolateral folds absent; discoidal fold present; dorsolateral vermiculation visible; ventral surfaces of thighs smooth; skin in cloacal region areolate; two ulnar tubercles present, low, round and small surrounded by other smaller and inconspicuous, outer palmar tubercle large, partially divided distally; inner palmar tubercle large and elongate; subarticular tubercles well-defined, pronounced round in ventral and lateral view; all fingers bearing elongated hyperdistal tubercles; supernumerary tubercles present, rounded in dorsal and ventral views; broad lateral fringes on fingers; Finger I shorter than Finger II; discs on Finger I slightly expanded and rounded, discs on Finger II expanded, Fingers III and IV broadly expanded; tip of fingers truncate; pads on all fingers well-defined and surrounded by circumferential grooves (Fig. 21).

Hindlimbs slender; upper surfaces of hindlimbs smooth, posterior surfaces of thighs areolate; heel bearing several low tubercles (less prominent in preservative); tarsus without tubercles or folds; small but well-defined inner metatarsal tubercle, elliptical in ventral view and rounded in lateral view, outer metatarsal tubercle small, prominent, rounded in ventral view and conical in lateral view; plantar surface with supernumerary tubercles rounded and prominent in ventral and lateral views; subarticular tubercles well-defined, rounded in lateral and ventral views; all toes bearing elongated hyperdistal tubercles; broad lateral fringes in toes; basal webbing between toes present; discs on toes nearly as large as those on fingers, expanded on toes I–III, broadly expanded on toes IV and V; all discs have large and well-defined pads surrounded by well-defined circumferential grooves; relative lengths of toes I < II < III < V < IV; Toe V much longer than Toe III (Toe III surpasses the distal border of the distal tubercle of Toe V and reaches the middle of the penultimate tubercle of Toe IV; Toe V surpasses the distal border of the distal tubercle of Toe IV) (Fig. 21).

**Variation (Fig. 22):** In this section, traits refer to living individuals unless otherwise stated; we only mention character states not observed in the holotype. Flanks skin might be shagreen (QCAZ 66372); dorsolateral vermiculation might be not visible (QCAZ 66372 and QCAZ 66467); two scapular tubercles on each side, subconical in lateral view and rounded in dorsal view can be present (QCAZ 66523); discoidal fold not visible in QCAZ 66523.

**Distribution, natural history, and conservation status (Fig. 1)**: *Pristimantis resistencia* sp. nov. is known from the surroundings of Ankaku Community in Llanganates National Park, (elevation range is 2,271–2,451 m). Ecosystem type is Eastern Montane Forest as defined by Ron, Merino-Viteri & Ortiz (2021). Specimens were collected at night over vegetation, perching on leaves or branches around 60 to 300 cm above the ground in cloud forest with abundant presence of epiphytes. Holotype (QCAZ 66519) and paratype (QCAZ 66523) were collected during a rainy night.

Because of the lack of information on population size and geographic distribution, we recommend assigning *P. resistencia* sp. nov. to the Data Deficient IUCN Red List Category (based on IUCN, 2019 guidelines); nonetheless, images obtained from Ministerio del Ambiente del Ecuador (2018) show deforested areas at 7.2 km from the collection localities (Fig. 1) which shows the worrying advance of the use of the land for anthropic purposes, mainly for livestock farming.

**Etymology:** This species is dedicated to all Latin American nature defenders who have been killed for denouncing environmental and human rights abuses. Since 2012, Latin America has been the most dangerous continent for activists and environmental defenders, with half of the crimes committed against indigenous community members (Sierra, 2019 and Santaeulalia, 2021). The murders are mainly associated with conflicts with mining, oil, logging, and large-scale monoculture companies (Sierra, 2019; de Miguel, 2021; Santaeulalia, 2021). The specific epithet *resistencia* is derived from the Spanish *resistencia* which means resistance and refers to the fight for environmental conservation.

***Pristimantis venegasi* sp. nov.**

urn:lsid:zoobank.org:act:6E60CD7B-B045-42E0-9C3D-4DFEE556ACE7

**Figure 23:**
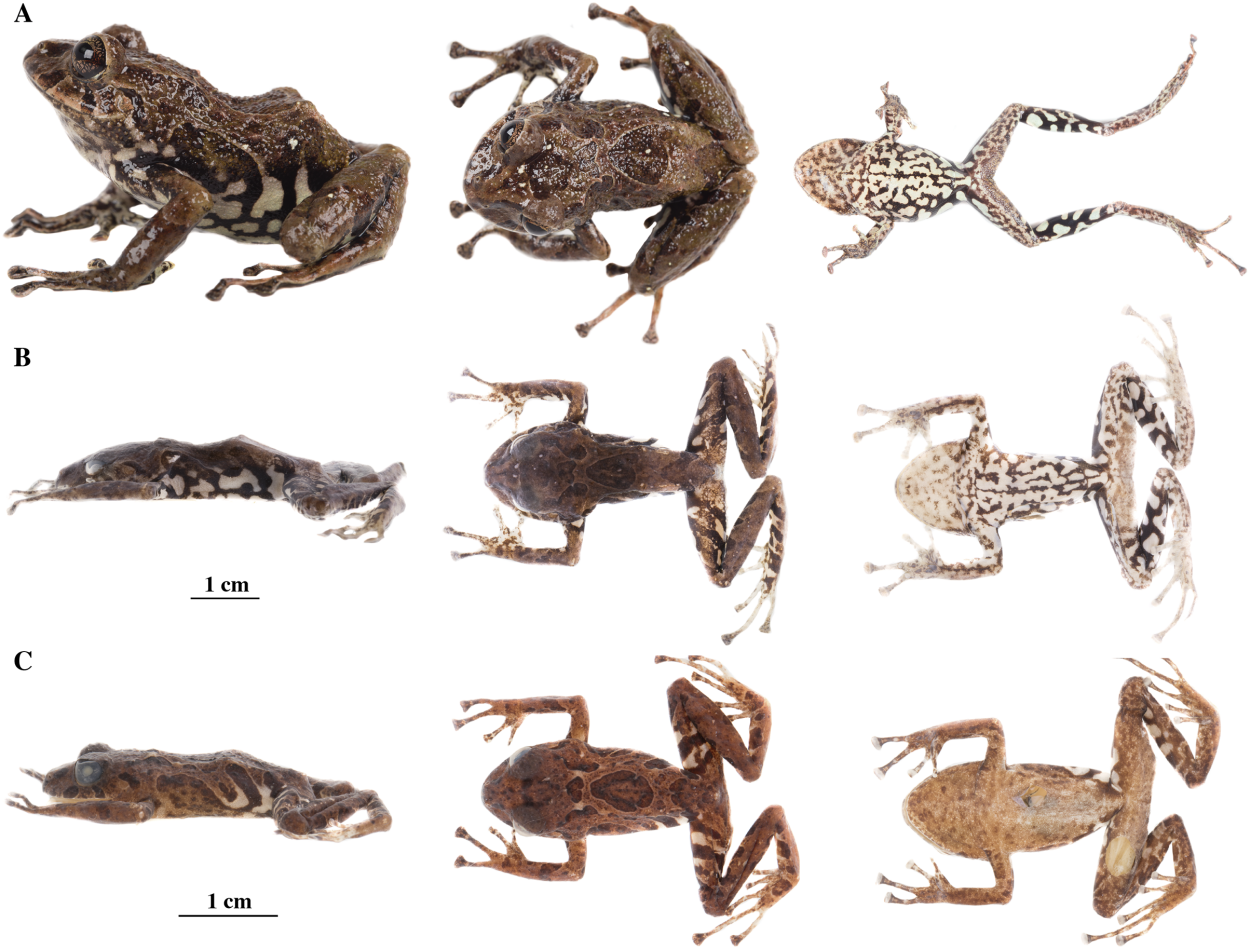
*Pristimantis venegasi* sp. nov. Holotype, QCAZ 66440, adult female, SVL = 34.90 mm. (A) Photographs holotype in life in lateral, dorsal and ventral views. (B) Photographs of preserved individual in lateral, dorsal and ventral views. (C) Photographs of preserved paratype, QCAZ 31130, adult male, SVL = 24.49 in lateral, dorsal and ventral views.

**Figure 24:**
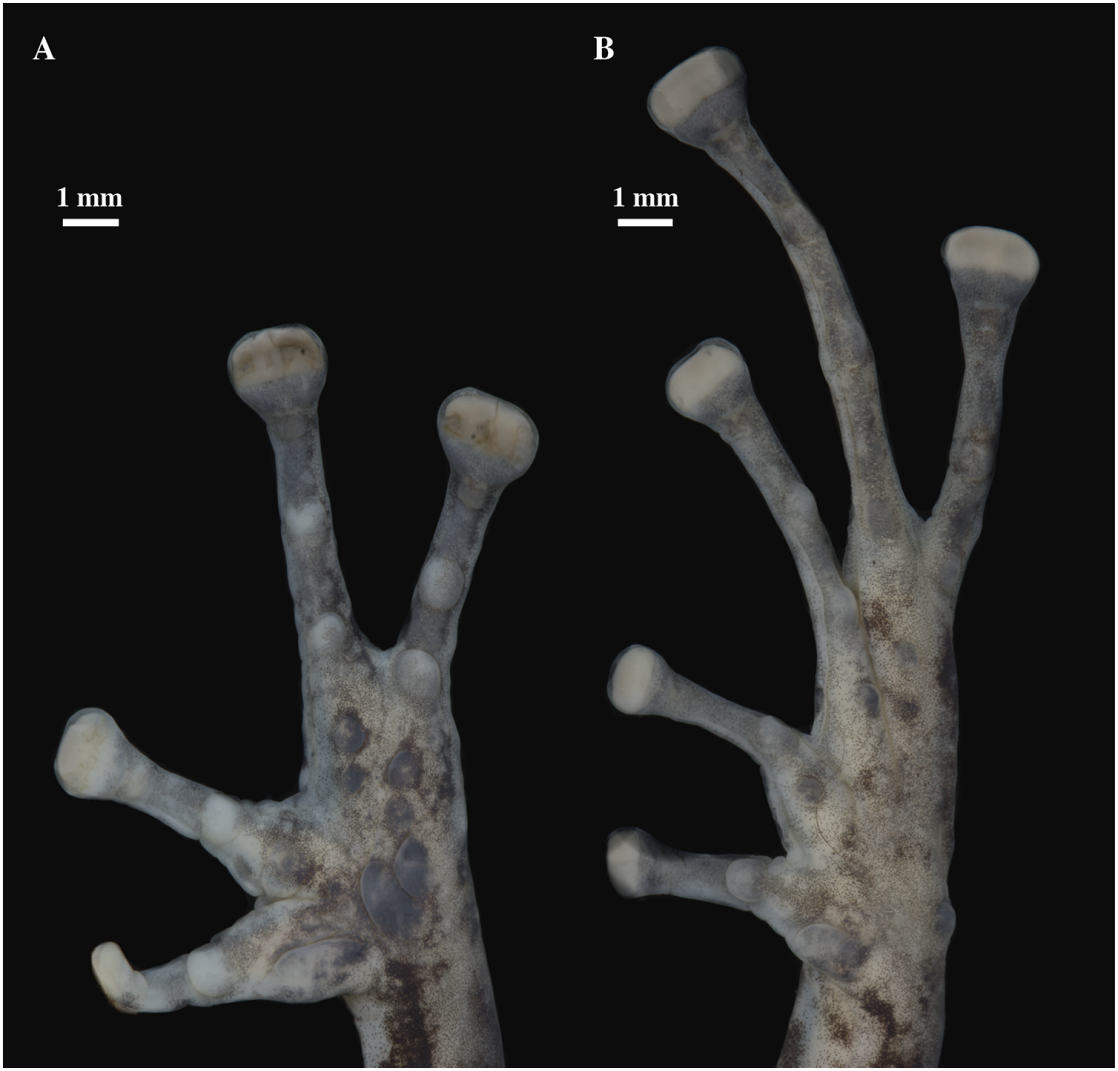
(A) Palmar and (B) plantar surfaces of *Pristimantis venegasi* sp. nov. Photographs of left hand and foot of the holotype QCAZ 66440.

**Holotype (Figs. 23, 24):** QCAZ 66440 (field no. SC-PUCE 52702), adult female from Ecuador, Pastaza Province, canton Mera, Parish Mera, San Rafael, Ankaku Community Reserve, Buffer zone of Llanganates National Park (1.2738° S; 78.0644° W), 2,110 m, collected by Diego Almeida, Santiago Guamán, Darwin Núñez, María José Navarrete, Verónica Andrade, Ángel Alvarado and Fernando Alvarado on January 26, 2017.

**Paratype (*n* = 1; Fig. 23):** QCAZ 31130, adult male from Ecuador, Napo Province, Entrance to the buffer zone, Sumaco National Park (0.7259° S; 77.5660° W), 1,500 m, collected by Néstor Acosta, Ítalo Tapia and Mónica Páez on December 12, 2005.

**Common name:** English: Venegas Rain Frog. Spanish: Cutín de Venegas.

**Diagnosis (Figs. 23, 24):** We assign the new species to the genus *Pristimantis* based on the phylogeny (Fig. 5). A species of *Pristimantis* characterized by: (1) skin of the dorsum tuberculate with scattered large conical or rounded tubercles, skin on venter areolate; (2) discoidal fold present; (3) dorsolateral folds absent; (4) tympanic membrane present, tympanic annulus present; (5) supratympanic fold present; (6) several postrictal tubercles present; (7) snout broadly rounded to rounded in dorsal view and rounded in profile; (8) upper eyelid with one conical tubercle and few lower tubercles; (9) cranial crests absent; (10) vocal slits and nuptial pads absent; (11) Finger I shorter than Finger II, discs on Finger I slightly expanded to expanded and rounded, discs on Fingers II–IV broadly expanded, truncate; (12) fingers with broad lateral fringes (may become inconspicuous due to the effect of preservation), all fingers with elongated and thin hyperdistal tubercles; (13) one ulnar tubercle present, small and rounded; (14) heel bearing several low tubercles, tarsus without tubercles; (15) toes with broad lateral fringes, basal webbing present, all toes with elongated and thin hyperdistal tubercles; (16) Toe V much longer than Toe III, Toe condition C (Toe III surpasses the distal border of the distal subarticular tubercle of Toe V and reaches the distal border of the penultimate tubercle of Toe IV; Toe V reaches the distal border of the distal tubercle of toe IV); (17) dorsum dark brown with light brown-green blotches with cream edges; large sacral dark round areas with thin clear borders; groins, ventral surfaces, and anterior and posterior surfaces of thighs with black and white marbling; bronze-colored iris with a transverse copper stripe and thick black reticulation; (18) SVL in adult female 34.90 (*n* = 1); SVL in adult male 24.49 mm (*n* = 1) (Tables 4, S2).

**Comparison with other species:** *Pristimantis venegasi* sp. nov. resembles *P. anaiae* sp. nov., *P. glendae* sp. nov., *P. kunam* sp. nov., *P. resistencia* sp. nov. and *P. katoptroides* by having dark marks with thin pale edges on the sides of the sacrum; it can be easily distinguished from them by the black and white marbling on ventral surfaces and groins (cream to dark brown bearing black or dark brown tubercles in *P. anaiae* sp. nov., whitish cream background with dark spots in *P. glendae* sp. nov., dark greenish with dark flecks in *P. kunam* sp. nov., venter solid white and greenish blue groins in *P. katoptroides*) and the presence of broad lateral fringes in fingers (narrow in *P. anaiae* sp. nov., *P. glendae* sp. nov., *P. kunam* sp. nov. and *P. katoptroides*). Besides, *P. venegasi* sp. nov. differs from *P. kunam* sp. nov. by having basal webbing (basal webbing absent in *P. kunam* sp. nov.) In addition, *P. venegasi* resembles *P. llanganati* and *P. roni* by having spiny appearance. It can be distinguished from them by the presence of basal webbing between toes (absent in *P. llanganati* and *P. roni*), the absence of vocal slits (present in *P. llanganati* and *P. roni*), the presence of large sacral dark round areas with thin clear borders (absent in *P. llanganati* and *P. roni*), and groins with black and white marbling (groins white or tan with distinct dark brown to black diagonal lines in *P. llanganati* and orange-green in females and reddish brown in males in *P. roni*). *Pristimantis venegasi* also resembles *P. mutabilis* and *P. verecundus* from the western foothills of the Andes by the presence of large sacral dark round areas with thin clear borders. It can be easily distinguished by having black and white marbling groins (red groins in *P. mutabilis* and *P. verecundus*), snout broadly rounded to rounded (snout subacuminate in *P. verecundus*), and absence of dorsolateral folds (dorsolateral folds present in *P. mutabilis* and *P. verecundus*).

**Description of the holotype (Figs. 23, 24):** Live and preserved coloration is shown in Fig. 22. Adult female (QCAZ 66440). Measurements (in mm): SVL 34.90; tibia length 18.59; foot length 17.63; head length 11.51; head width 13.93; eye diameter 4.15; tympanum diameter 1.36; interorbital distance 3.14; upper eyelid width 2.94; internarial distance 2.86; eye-nostril distance 3.74.

Head wider than long, slightly wider than body, snout broadly rounded in dorsal view and rounded in profile; canthus rostralis straight in lateral view; loreal region concave; cranial crests absent, upper eyelid bearing one small conspicuous conical tubercle surrounded by few indistinct smaller tubercles; tympanic annulus present, more conspicuous on its ventral portion; tympanic membrane present; several rounded, low, postrictal tubercles. Supratympanic fold present, large and dark brown. Dentigerous processes of vomers present, oblique, separated, positioned posterior to the level of choanae; each vomer bearing several inconspicuous small teeth; vocal slits and nuptial pads absent.

Skin on dorsum tuberculate with scattered large and conical tubercles, skin on flanks areolate with scattered tubercles dorsally (tubercles become inconspicuous in fixed specimen), dorsolateral folds absent; discoidal fold present; skin on throat, chest, and belly areolate; ventral surfaces of thighs smooth; skin in cloacal region areolate. One ulnar tubercle present, small and rounded; palmar tubercles low but conspicuous, outer palmar tubercle big, partially divided distally; inner palmar tubercle big and elongate; subarticular tubercles well-defined, pronounced round in ventral and lateral view, all fingers with elongated and thin hyperdistal tubercles; supernumerary tubercles present, prominent, rounded in dorsal and ventral views; lateral fringes on fingers broad; Finger I shorter than Finger II; discs on Finger I expanded and rounded, discs on Fingers II–IV broadly expanded; discs truncate; pads on all fingers well-defined and surrounded by circumferential grooves (Fig. 24).

Hindlimbs slender; upper surfaces of hindlimbs with small, rounded, and not prominent tubercles; posterior surfaces of thighs areolate; heel bearing several low tubercles; tarsus without tubercles; tarsal fold absent; well-defined but small inner metatarsal tubercle, elliptical in ventral view and rounded in lateral view, outer metatarsal tubercle small, prominent, rounded in ventral view and subconical in lateral view; plantar surface with supernumerary tubercles rounded and prominent in ventral and lateral view; subarticular tubercles well-defined, rounded in lateral and ventral views, all toes with elongated and thin hyperdistal tubercles; broad lateral fringes in toes; basal webbing between toes; discs nearly as large as those on fingers, expanded on toes I–III, broadly expanded on toes IV and V; all discs have large and well-defined pads surrounded by well-defined circumferential grooves; relative lengths of toes I < II < III < V < IV; Toe V much longer than Toe III (Toe III surpasses the distal border of the distal tubercle of Toe V and reaches the distal border of the penultimate tubercle of Toe IV; Toe V reaches the distal border of the distal tubercle of toe IV) (Fig. 24).

**Variation ( Fig. 24):** In this section, traits refer to living individuals unless otherwise stated; we only mention character states not observed in the holotype. Male (QCAZ 31130) is smaller (SVL = 24.49) than the single known female. QCAZ 31130 also has a tuberculated dorsum with more prominent tubercles and rounded snout in dorsal view; broad lateral fringes on fingers (ill-defined as an effect of preservation); disc on Finger I less expanded than in holotype.

**Distribution, natural history, and conservation status ( Fig. 1)**: *Pristimantis venegasi* is known from the surroundings of Ankaku Community, Llanganates National Park (elevation range is 1,500–2,110 m). Ecosystem type is Eastern Montane Forest as defined by Ron, Merino-Viteri & Ortiz (2021). Specimens were collected at night along trails, perching on leaves or branches ~170 cm from ground, in cloud forest with abundant epiphytes. The locality within Ankaku Community (LNP), where specimen QCAZ 66440 was found, shows no disturbed areas within a 5 km radius, but is 6 km from the nearest deforested area, a distance that undoubtedly shows the worrying advance of the agricultural frontier. On the other hand, the locality in Sumaco where the paratype QCAZ 31130 was collected in 2005 is currently deforested. According to data on vegetation cover and land use from the Ministerio del Ambiente del Ecuador (2018), these lands are currently agricultural lands. The population status of *Pristimantis venegasi* is unknown. The area deforested for agriculture and livestock production corresponds to 26.1% of the total area of distribution of *P. venegasi* within a buffer of 5 km (Ministerio del Ambiente del Ecuador, 2018).

Because of the lack of information on population size and geographic distribution, we recommend assigning *P. venegasi* to the Data Deficient IUCN Red List Category (based on IUCN, 2019 guidelines).

**Etymology:** The specific epithet is a patronym for Pablo Venegas, Peruvian herpetologist, in recognition of his contributions to the study of the diversity of Neotropical amphibians and reptiles. The species epithet is formed from the last name “Venegas” as a noun in the genitive case, with the Latin suffix “i” (ICZN 31.1.2).

**Redefinition of the subgenus *Huicundomantis***

Our phylogeny (Fig. 4) shows that *P. mallii*, *P. miktos*, and *P. tamia* sp. nov. form a clade sister to the subgenus *Huicundomantis* (*sensu* Páez & Ron, 2019). We propose to expand the definition of the subgenus *Huicundomantis* to include *P. mallii*, *P. miktos*, and *P. tamia* sp. nov. We redefine the subgenus *Huicundomantis* below. Under its new definition, *Huicundomantis* has strong support (bootstrap = 88; Fig. 4).

Subgenus *Huicundomantis* Páez & Ron, 2019

Type species: *Pristimantis phoxocephalus* (Lynch, 1979).

Definition: This clade is strongly supported by genetic evidence (Fig. 4). Morphological synapomorphies are unknown. Members of this clade are characterized by: (i) dorsolateral folds absent (except for *P. atratus*); (ii) cranial crests absent (except for *P. atratus, P. percultus*, and *P. spinosus*); (iii) tympanic annulus present (except in *P. philipi*); (iv) tympanic membrane present or absent; (v) dentigerous processes of vomer present; (vi) small to prominent tubercles on heel; (vii) fingers and toes with lateral fringes; (viii) basal webbing between toes present or absent; (ix) Toe V longer to much longer than Toe III; (x) in life, groins and concealed surfaces of thighs with distinctive coloration patterns including flash colors and light or bright-colored flecks or spots on a darker background (except in *P. miktos, P. lojanus*, and *P. tamia* sp. nov.; Szèkely et al., 2021); (xi) SVL females 22.6–46.8 mm; SVL males 11.6–34.5 mm (Ortega-Andrade & Venegas, 2014; Reyes-Puig et al., 2019; Szèkely et al., 2021).

Content: This clade comprises 28 described species (one of them described below) *P. andinogigas* Yánez-Muñoz et al., 2019; *P. atillo* Páez & Ron, 2019, *P. atratus* (Lynch, 1979), *P. balionotus* (Lynch, 1979), *P. chomskyi* Páez & Ron, 2019, *P. cryptomelas* (Lynch, 1979), *P. gagliardoi* Bustamante & Mendelson, 2008, *P. gloria* Páez & Ron, 2019, *P. hampatusami* Yánez-Muñoz, Sánchez-Nivicela & Reyes-Puig, 2016, *P. jimenezi* Páez & Ron, 2019, *P. lojanus* Szèkely et al., 2021, *P. lutzae* Páez & Ron, 2019, *P. mallii* Reyes-Puig et al., 2019, *P. miktos* Ortega-Andrade & Venegas, 2014, *P. multicolor* Páez & Ron, 2019, *P. muscosus* (Duellman & Pramuk, 1999), *P. nangaritza* Páez & Ron, 2019, *P. percultus* (Lynch, 1979), *P. philipi* (Lynch & Duellman, 1995), *P. phoxocephalus* (Lynch, 1979), *P. spinosus* (Lynch, 1979), *P. tamiae* sp. nov., P. *teslai* Páez & Ron, 2019, *P. tinguichaca* Brito et al., 2016, *P. torresi* Páez & Ron, 2019, *P. totoroi* Páez & Ron, 2019, *P. versicolor* (Lynch, 1979), and *P. verrucolatus* Páez & Ron, 2019. This clade encompasses three species groups: the *P. cryptomelas* species group Páez & Ron, 2019, the *P. phoxocephalus* species group Páez & Ron, 2019 and the newly defined *P. miktos* species group (see below).

Distribution: *Huicundomantis* occurs in Eastern and Western Andean slopes and Inter-Andean valleys of southern and central Ecuador, and Eastern Andean slopes of northern Peru. A single species, *Pristimantis miktos*, inhabits the Amazonian lowlands, between 195 and 300 m.a.s.l. (Ortega-Andrade & Venegas, 2014). *Huicundomantis* occurs in the following Natural Regions: Deciduous Costa Forest, Western Foothill Forest, Western Montane Forest, Paramo, Inter-Andean Shrub, Eastern Montane Forest, and Eastern Foothill Forest, and Amazonian Tropical Rain Forest, between 195 and 4,200 m.a.s.l.

Remarks: Ortega-Andrade & Venegas (2014) report that *P. miktos* lacks lateral fringes in fingers and toes. That would be the only instance of absence of fringes for the subgenus *Huicundomantis*. We examined the type material of *P. miktos* and found distinctive lateral fringes in fingers and toes. Therefore, the presence of lateral fringes is shared by all species of *Huicundomantis*.

***PRISTIMANTIS MIKTOS* SPECIES GROUP**

**Definition:** The *P. miktos* species group is strongly supported in our phylogeny. Members of this group share the following morphological traits: (i) small to medium-sized frogs with SVL from 10.2 to 21.3 mm in males and from 22.6 to 34 mm in females; (ii) slender to moderate robust bodies; (iii) dorsum smooth to shagreen; (iv) scapular and discoidal folds present; (v) dorsolateral folds absent; (vi) tympanic annulus present; (vii) tympanic membrane present or not; (viii) snout round in lateral view and subacuminate to round in dorsal view; (ix) cranial crests absent; (x) upper eyelid and heels bearing tubercles; (xi) vocal slits present or not; (xii) basal webbing on toes absent; (xiii) Finger I shorter than Finger II; (xiv) Toe V much longer than Toe III, Toe condition C (Toe III reaches the distal border of the distal subarticular tubercle of Toe V and the proximal border of the penultimate tubercle of Toe IV; Toe V reaches the middle of the distal tubercle of toe IV); (xv) discs on fingers expanded; (xvi) all fingers and toes bearing lateral fringes and elongated hyperdistal tubercles.

**Content:** Currently, the group contains three described species: *Pristimantis mallii, P. miktos*, and *P. tamia*. Two putative undescribed species are Clade G and Clade F in Fig. 4.

**Distribution:** Members of *P. miktos* species group occur in the eastern Andean slopes of central and southern Ecuador and the Amazonian lowlands of Ecuador and northern Perú.

***Pristimantis tamia* sp. nov.**

urn:lsid:zoobank.org:act:458A8123-E7B4-4F10-BB0C-0CB97BFD67D1

**Figure 25:**
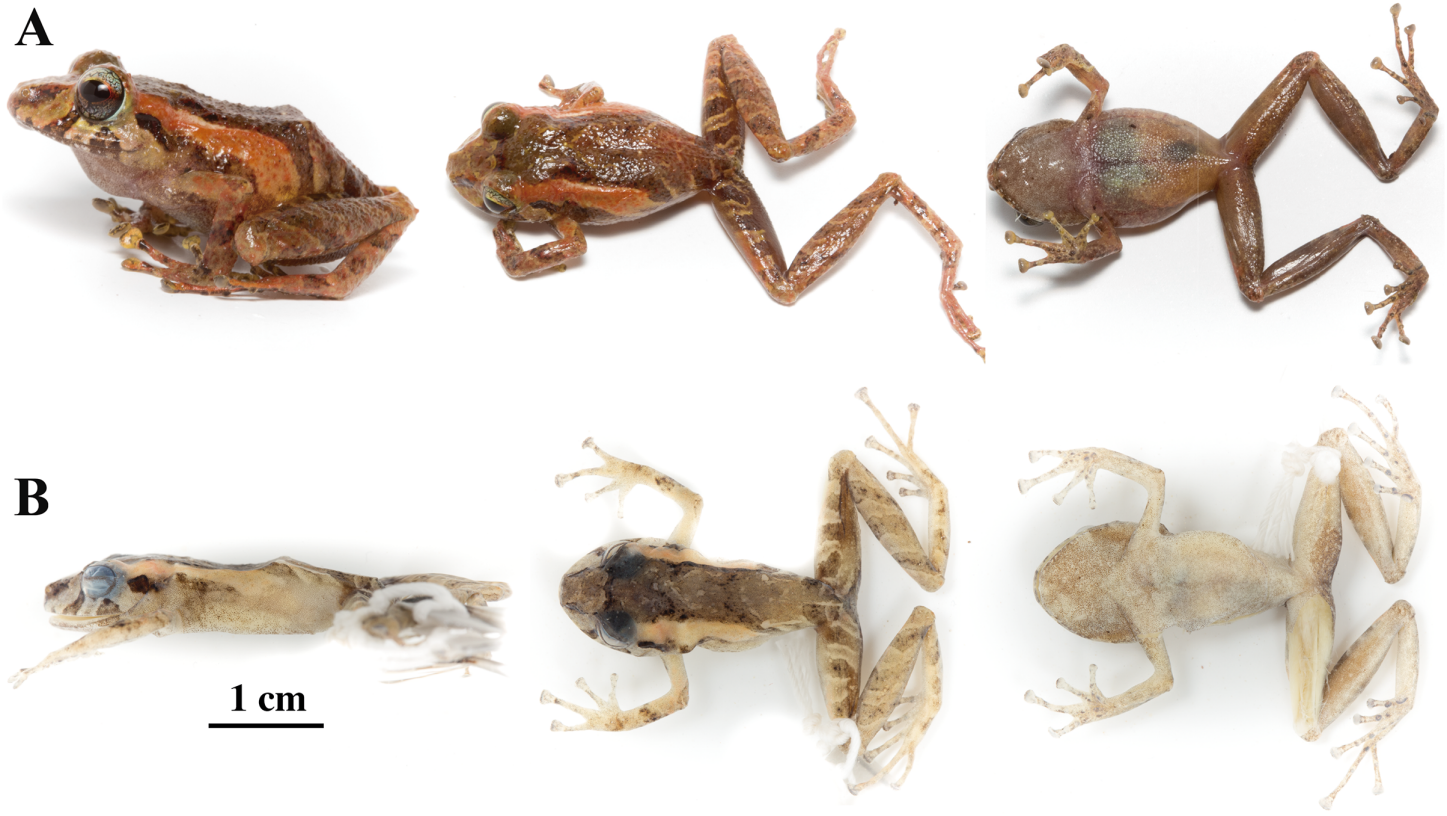
*Pristimantis tamia* sp. nov. Holotype, QCAZ 59573, adult female, SVL = 25.41 mm. (A) Photographs of alive individual in lateral, dorsal and ventral. (B) Photographs of preserved individual in lateral, dorsal and ventral view.

**Figure 26:**
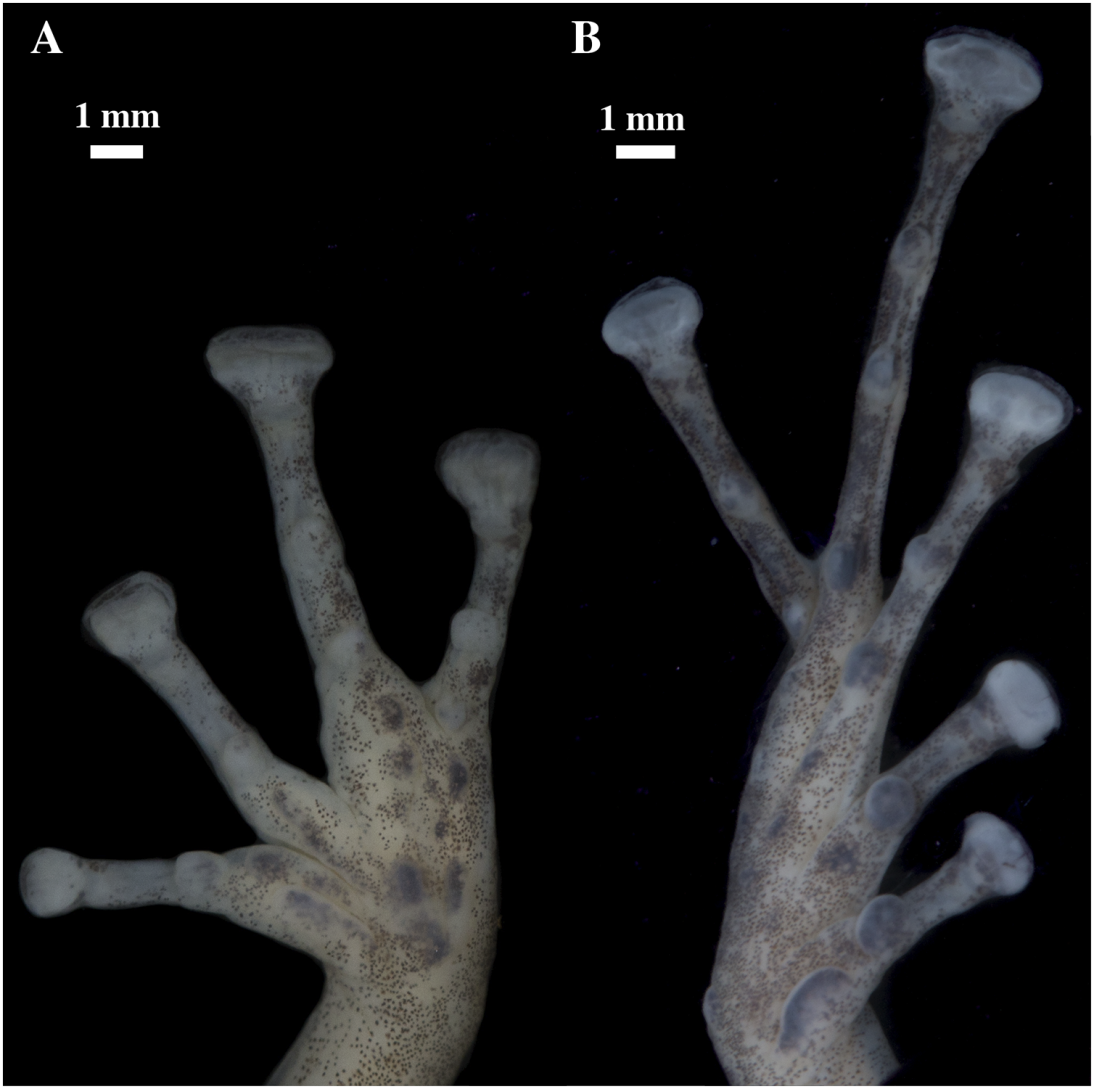
(A) Palmar and (B) plantar surfaces of *Pristimantis tamia* sp. nov. Photographs of left hand and right foot of the holotype QCAZ 59573.

**Figure 27:**
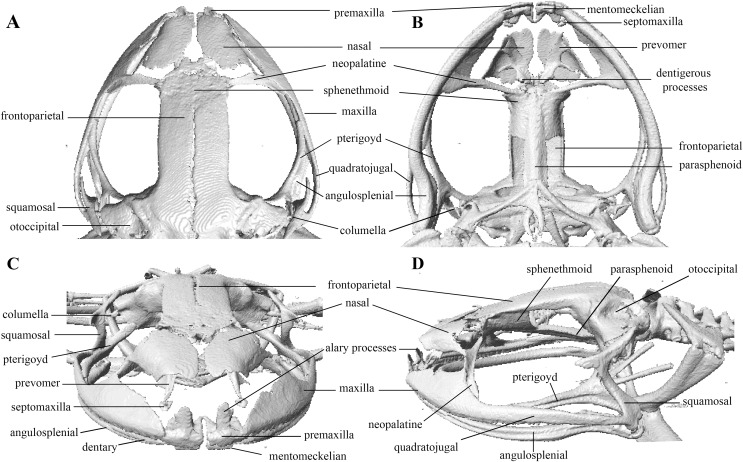
Head skeleton of *Pristimantis tamia sp*. nov. Holotype QCAZ 59573. The skull is shown in: (A) dorsal view; (B) ventral view; (C) frontal view; (D) lateral view.

**Figure 28:**
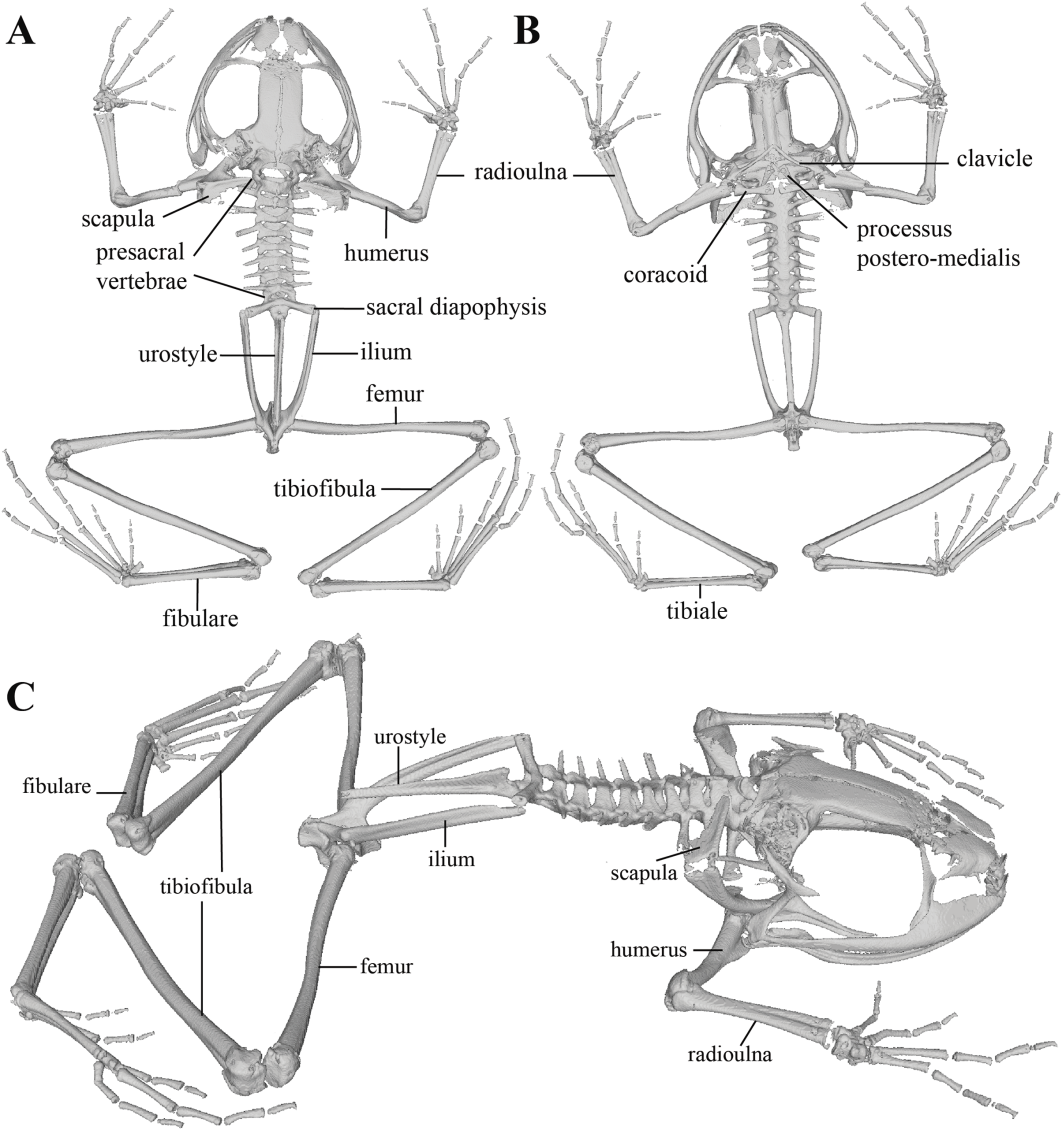
Whole skeleton of *Pristimantis tamia* sp. nov. Holotype QCAZ 59573. The skeleton is shown in (A) Dorsal view. (B) Ventral view. (C) Dorsolateral view.

**Figure 29:**
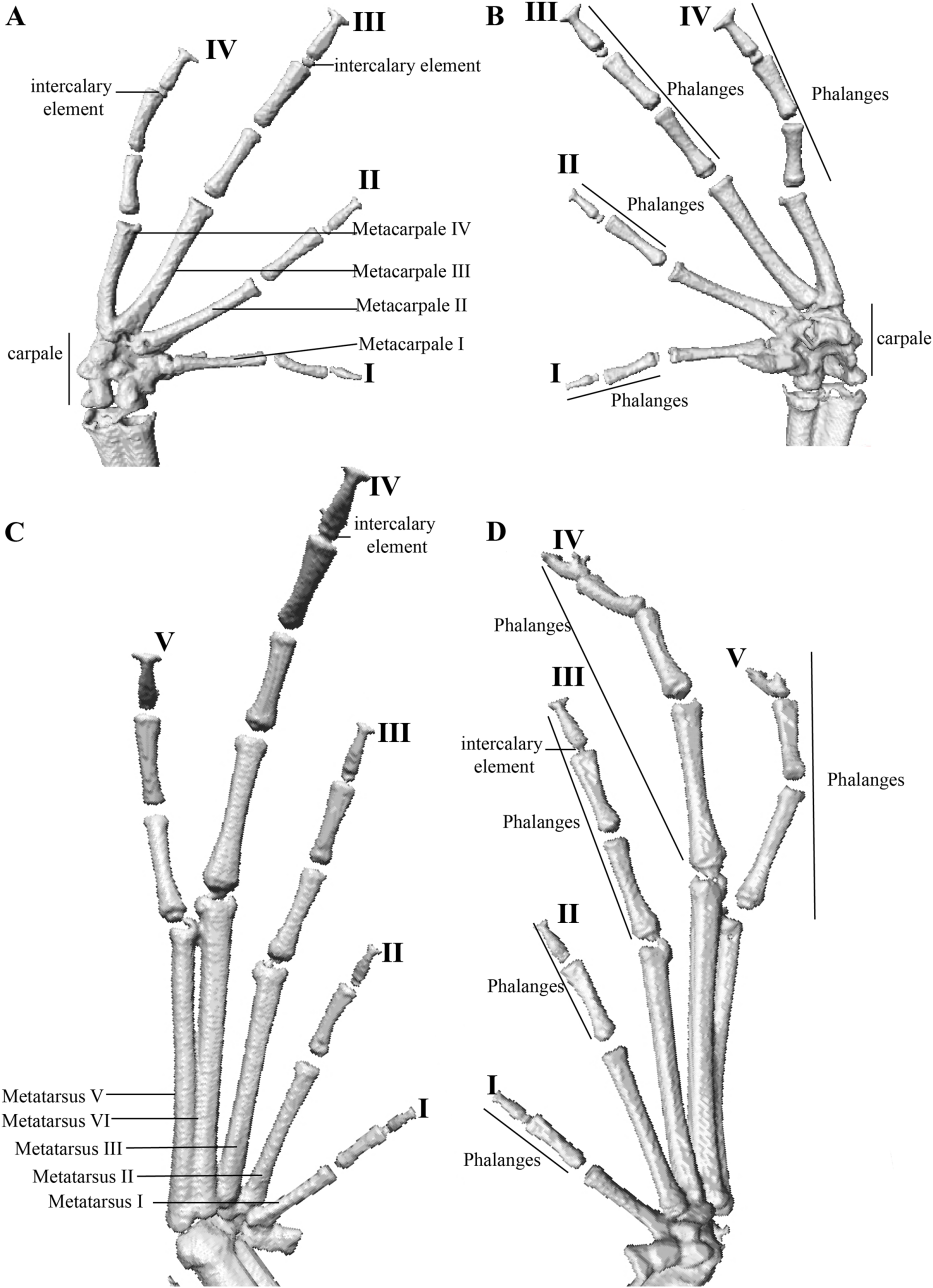
Hand and foot osteology of *Pristimantis tamia* sp. nov. Holotype QCAZ 59573. The left hand is shown in (A) dorsal and (B) ventral (palmar) views. The left foot is shown in (C) dorsal and (D) ventral (plantar) views.

**Holotype (Figs. 25–29)**: QCAZ 59573 (field no. SC-PUCE 49349) adult female from Ecuador; Pastaza Province, Canton Mera, Llanganates National Park, Community Zarentza, school surroundings (1.3594° S; 78.0557° W), 1,388 m. Collected by Daniel Rivadeneira, Francy Mora, Juan Carlos Sánchez, David Velalcázar, Darwin Núñez and Javier Pinto on February 15, 2015.

**Figure 30:**
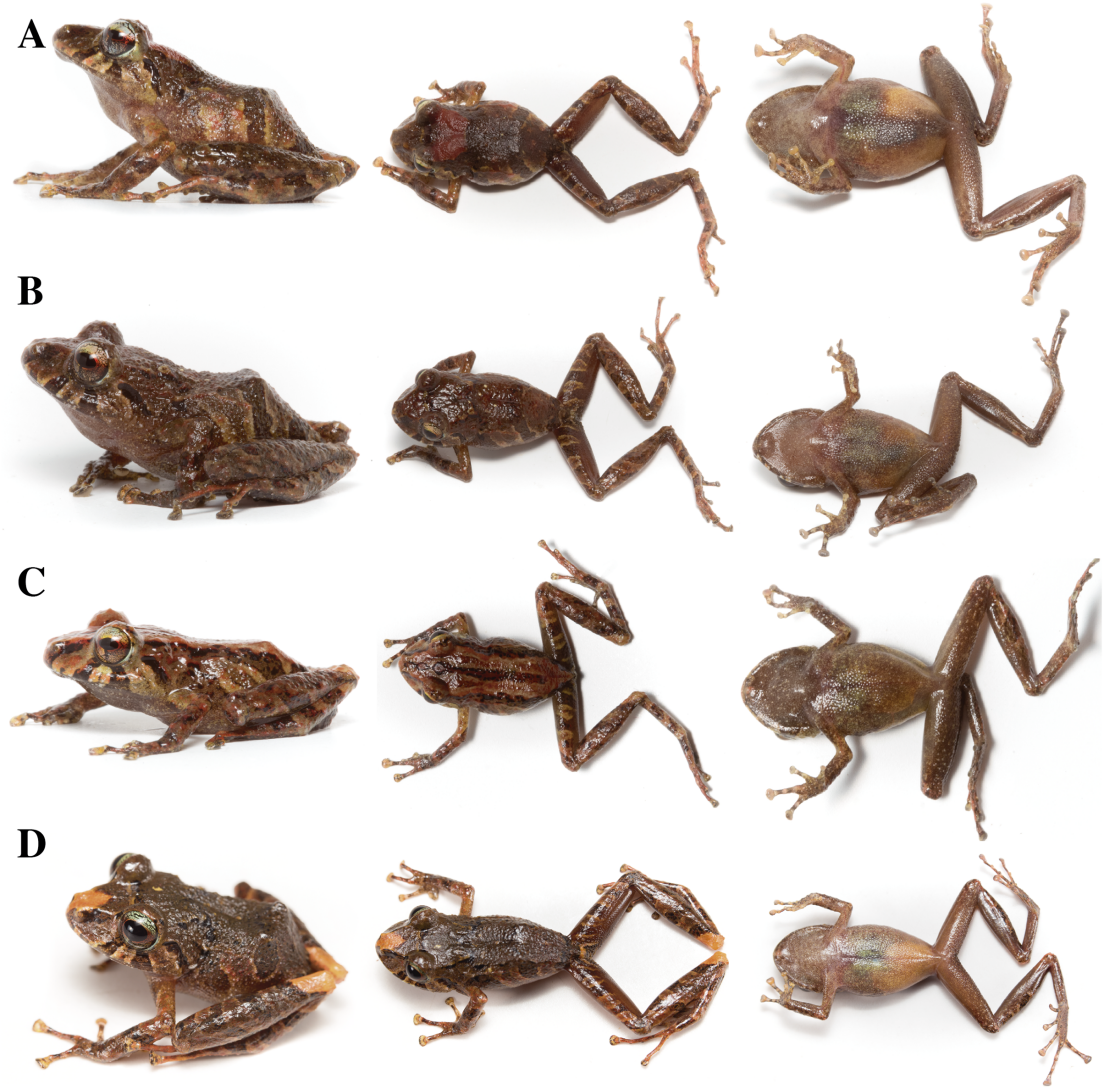
Color variation in life individuals of *Pristimantis tamia* sp. nov. (A) QCAZ 59564, paratype, adult female, SVL = 25.65 mm. (B) QCAZ 59582, paratype, adult female, SVL = 25.78 mm. (C) QCAZ 59620, paratype, adult male, SVL = 19.26 mm. (D) QCAZ 59636, paratype, adult female, SVL = 26.89 mm. Lateral view on the left, dorsal view in the center and ventral view on the right.

**Figure 31:**
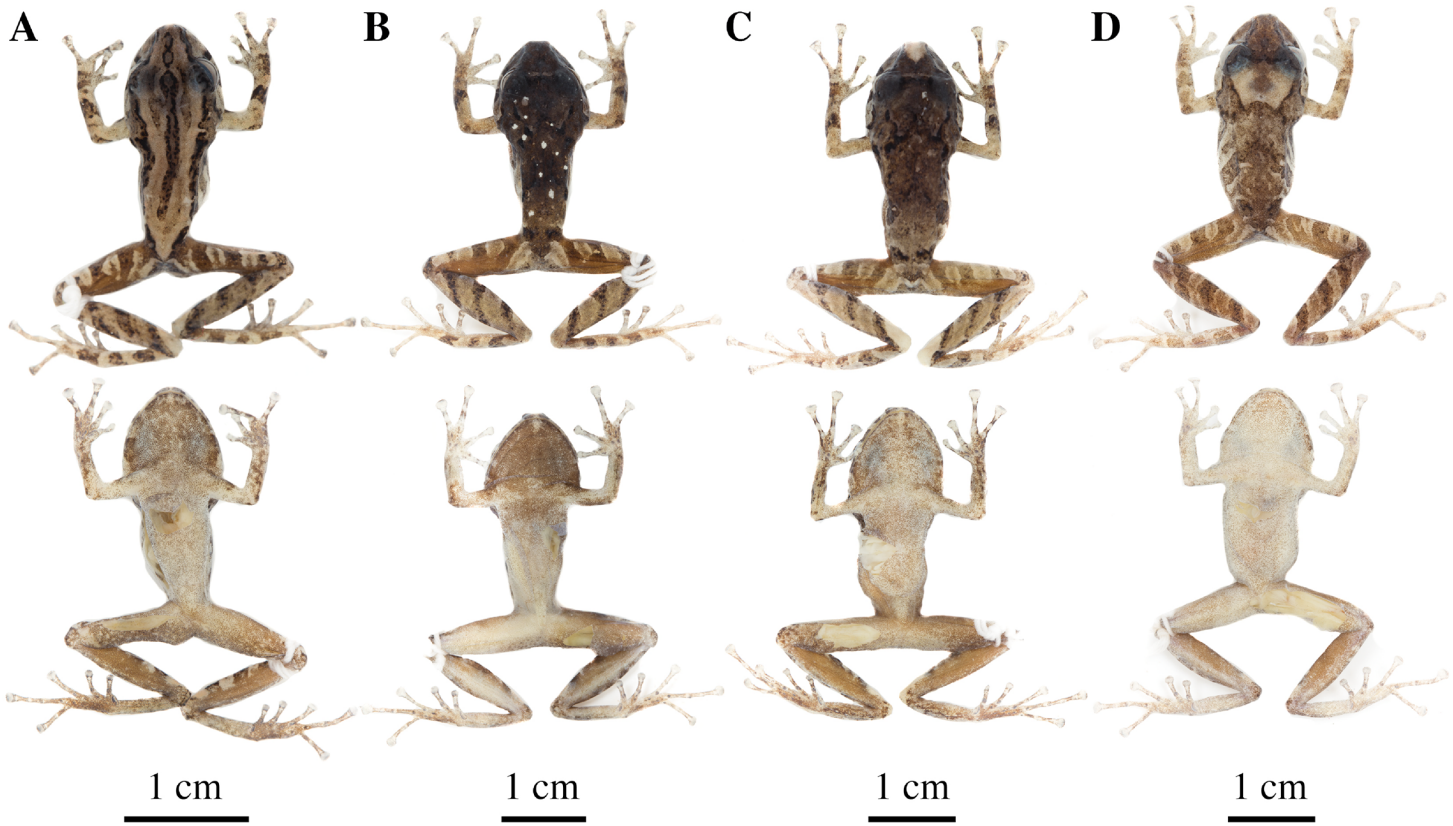
Color variation in preserved individuals of *Pristimantis tamia* sp. nov. (A) QCAZ 59620, paratype, adult male, SVL = 19.26 mm. (B) QCAZ 59630, paratype, adult female, SVL = 26.53 mm. (C) QCAZ 59636, paratype, adult female, SVL = 26.89 mm. (D) QCAZ 59643, paratype, adult female, SVL = 26.58 mm. Dorsal and ventral variation is shown for each specimen.

**Paratypes (*n* = 38; Figs. 30, 31):** All from Ecuador; Pastaza Province, Canton Mera, Llanganates National Park, Community Zarentza. Same locality as holotype, QCAZ 59565 adult male, 1.3594° S; 78.0578° W, 1,357 m, QCAZ 59564 adult female, 1.3564° S; 78.0581° W, 1,367 m, QCAZ 59581 adult female, 1.3571° S; 78.0537° W, 1,332 m. Path to the Yurugyaku river, QCAZ 59439 adult female, 1.3524° S; 78.0597° W, 1,419 m; QCAZ 59445 adult male, 1.3524° S; 78.0681° W, 1,419 m; QCAZ 59635–36 adult males; QCAZ 59642–44 adult females, QCAZ 59639 juvenile, 1.3472° S; 78.0813° W, 1,380 m; QCAZ 59651, QCAZ 59653, QCAZ 59660, QCAZ 59664, QCAZ 59668, QCAZ 59675 adult males; QCAZ 59650, QCAZ 59656, QCAZ 59666 adult females; QCAZ 59672 juvenile, 1.3524° S; 78.0681° W, 1,419 m; QCAZ 59680 adult female, 1.3525° S; 78.0756° W, 1,419 m; QCAZ 59696 adult male, 1.3397° S; 78.0595° W, 1,360 m. Trail to Waterfalls, QCAZ 59582, QCAZ 59584 adult females, 1.3543° S; 78.0621° W, 1,338 m. QCAZ 59585, QCAZ 59593 adult males, 1.3544° S; 78.0621° W, 1,388 m; QCAZ 59619–20 adult males, QCAZ 59629–30 adult females, 1.3570° S; 78.0581° W, 1,354 m. Surroundings of the house of Gustavo Ushpa, QCAZ 59702, QCAZ 59713 adult males; QCAZ 59704–05, QCAZ 59701, QCAZ 59710 adult females, 1.3431° S; 78.0574° W, 1,221 m. Tributaries of the river Nuchimingue, QCAZ 59719 adult female, 1.3626° S; 78.0578° W, 1,391 m. All collected by Daniel Rivadeneira, Francy Mora, Juan Carlos Sánchez, David Velalcázar, Darwin Núñez and Javier Pinto on February 14 to 27 of 2015.

**Common name:** English: Tamia Rain Frog. Spanish: Cutín de Tamia.

**Diagnosis (Figs. 25–31):** We assign the new species to the genus *Pristimantis* based on the phylogeny (Fig. 4). A species of *Pristimantis* characterized by the following combination of characters: (1) skin of dorsum weakly tuberculate, on flanks weakly tuberculate to densely tuberculate, skin on belly areolate to coarsely areolate; (2) discoidal fold present; (3) tympanic annulus present; (4) tympanic membrane absent; (5) several postrictal tubercles present; (6) snout rounded in dorsal and lateral view; (7) upper eyelid with two conical tubercles surrounded by few smaller tubercles; (8) cranial crests absent; (9) vocal slits and nuptial pads absent; (10) Finger I shorter than Finger II, discs on digit I rounded, expanded on digit II and broadly expanded and truncate on digits III and IV; (11) fingers with narrow lateral fringes, all fingers with elongated and thin hyperdistal tubercles; (12) several ulnar tubercles present, rounded; (13) heel bearing a conical tubercle surrounded by smaller, rounded tubercles (absent in some individuals); (14) toes with broad lateral fringes, basal webbing absent, all toes with elongated and thin hyperdistal tubercles; (15) Toe V much longer than Toe III, Toe condition C (Toe V reaches the distal border of the distal tubercle of Toe IV; Toe III reaches the distal border of penultimate tubercle of toe IV); (16) dorsum orange to dark brown with light brown irregular chevrons or yellow spots; flanks dark brown with orange or yellowish dorsolateral band. Head light brown to dark brown bearing, in some individuals, a dark brown interorbital bar. Ventral surfaces cream to brown with dark brown flecks; iris violet to light greenish blue with a thin copper medial fringe and thick black reticulations; (17) SVL in adult females: 25.96 mm on average (21.27–29.88; *n* = 21); SVL in adult males: 18.28 mm on average (16.04–19.81; *n* = 15) (Tables 4, S3).

**Comparison with other species:** *Pristimantis tamia* resembles *P. incomptus* (Lynch & Duellman, 1980), *P. mallii P. martiae* (Lynch, 1974), and *P. miktos* by the absence of cranial crests, presence of tubercles on upper eyelids and heels, and expanded finger discs. It differs from them by having a light greenish blue iris and lacking vocal slits. Furthermore, *Pristimantis miktos, P. mallii*, *and P. incomptus* have tympanic membrane (absent in *P. tamia*), while *P. martiae* lacks tympanic annulus (present in *P. tamia*). In dorsal view, *Pristimantis miktos* has an acuminate snout, which is broadly rounded in *P. mallii*, subacuminate in *P. incomptus* and *P. martiae* and rounded in *P. tamia*. They also differ in iris color which is light greenish blue with a horizontal red fringe in *Pristimantis tamia*, bronze to red with black reticulations in *P. miktos*, coppery golden with black reticulation and a reddish brown horizontal band in *P. mallii*, bronze to gray with an horizontal reddish brown line in *P. incomptus*, and bronze with a brown horizontal line in *P. martiae*. They can also be distinguished by their ventral coloration. While in *Pristimantis tamia* the throat and belly are creamy brown uniformly covered with minute dark brown flecks, in *P. miktos* they are cream with small, scattered dots or mottled brown; the belly, in *P. incomptus* is gray, cream or brown with or without dark flecks, and in *P. martiae* grayish brown with small white to pale orange flecks. *Pristimantis tamia* can be easily distinguished from *P. mallii* by having W-shaped scapular folds (*P. mallii* has X*-*shaped scapular folds).

**Description of the holotype (Figs. 25–29):** Live and preserved coloration is shown in Figs. 25 and 26. Adult female (QCAZ 59573). Measurements (in mm): SVL 25.41; tibia length 14.78; foot length 11.54; head length 8.04; head width 9.86; eye diameter 3.72; tympanum diameter 1.09; interorbital distance 2.86; upper eyelid width 2.83; internarial distance 2.23; eye-nostril distance 3.50.

Head wider than long, head wider than body; snout rounded in dorsal and lateral view; canthus rostralis distinct, curved in dorsal view; cranial crests absent; upper eyelid bearing two conical tubercles surrounded by many lower and rounded tubercles; tympanic annulus distinct; tympanic membrane absent; two rounded postrictal tubercles with some ill-defined rounded tubercles surrounding them. Dentigerous processes of vomers oblique, broadly separated, positioned posteromedial to choanae; each vomer bearing several inconspicuous teeth; vocal slits and nuptial pads absent.

Skin on dorsum and flanks weakly tuberculate with low, conical tubercles, more densely grouped on posterior half of dorsum; dorsolateral vermiculation visible; skin on throat smooth, on chest shagreen and areolate on belly; ventral surfaces of thighs areolate; discoidal fold present; skin in upper cloacal region areolate with many rounded and low tubercles below the cloacal sheath. Four ulnar tubercles present, low and rounded; nuptial pads and vocal slits absent; palmar tubercles low, outer palmar tubercle bifurcate a little larger than the ellipsoid thenar tubercle; subarticular tubercles well-defined, big, round in ventral view and in profile, all fingers with elongated and thin hyperdistal tubercles; supernumerary tubercles at base of fingers, distinct, big and round; narrow lateral fringes on fingers; Finger I shorter than Finger II; disc on Finger I rounded, disc on Finger II expanded, discs on Fingers III and IV broadly expanded and truncate; pads on all fingers well-defined and surrounded by conspicuous circumferential grooves (Fig. 26).

Hindlimbs slender; upper surfaces of hindlimbs smooth; posterior and ventral surfaces of thighs smooth on their distal portion and slightly areolate on their proximal portion; heel with one conspicuous big conical tubercle with some low and rounded tubercles surrounding it; outer surface of tarsus bearing inconspicuous small rounded tubercles; inner tarsal fold present; inner metatarsal tubercle large and prominent (1.29 mm in its longest diameter), elliptical and rounded in lateral view, three times longer than the elliptical, not well-defined outer metatarsal tubercle (0.49 mm in its longest diameter); plantar surface with few and slightly visible supernumerary tubercles; subarticular tubercles well defined, big, and rounded; all fingers with elongated and thin hyperdistal tubercles; toes with broad lateral fringes; basal webbing between toes absent; discs much larger than those on fingers, broadly expanded in all toes; all toes having pads surrounded by well-defined circumferential grooves; relative lengths of toes: I < II < III < V < IV; Toe V much longer than Toe III, Toe condition C (Toe III reaches the distal border of the distal tubercle of Toe IV; Toe V reaches the distal border of the distal tubercle of toe IV) (Fig. 26).

**Variation (Figs. 30, 31):** In this section, traits refer to living individuals unless otherwise stated; we only mention character states not observed in the holotype. Adult males (16.04–19.81 mm; *n* = 15) are smaller than females (21.27–29.88 mm; *n* = 21). Belly skin might be coarsely areolate (*e.g*., QCAZ 59704). Flanks skin might be tuberculate (*e.g*., QCAZ 59666) or densely tuberculated (*e.g*., QCAZ 59704); dorsal skin may be weakly tuberculated from the tip of snout to iliac bones and densely tuberculated from iliac bones to cloacal region (*e.g*., QCAZ 59704). Small and round tubercles may be present between feet and heels (*e.g*., QCAZ 59582 and QCAZ 59704) and between elbows and wrists (*e.g*., QCAZ 59704). Discs on toes I and II may be not expanded (*e.g*., QCAZ 59666).

**Osteology:** The osteological description is based on micro-CT images of the holotype (adult female QCAZ 59573).

Skull (Fig. 27): The skull is slightly wider than long; widest part is at joint between quadratojugal and rostral branch of squamosal. Longest axis of skull is 98.9% of the widest axis and runs from anterior face of premaxilla to posterior face of exoccipital. Rostrum is short with a distance from anterior edge of frontoparietals to anterior face of premaxilla of approximately 22.4% of longest axis of skull. Skull is about 76.5% of maximum skull width at level of anterior edge of orbits and 91.8% at level of midorbit. Posterior edge of orbits is aligned to skull widest axis.

The skull contains well ossified elements. Frontoparietals are well-developed, markedly longer than wide and merged medially, with their lateral edges parallel to each other along their entire length; they possess a few non-ossified areas more conspicuous posteriorly. Posterior portion of skull is fully enclosed by complete fusion of frontoparietals with otoccipitals; the latter bear unossified patches on each side. Otoccipitals are formed by well-fused prootics and exoccipitals. Where frontoparietals articulate with otoccipitals, they form prominent crests on each side. Each crest is V-shaped, with a ~100 degrees angle directed medially; its posterior branch is 90.3% the length of anterior branch. Otoccipitals are ventrally articulated with parasphenoid alae.

Anteriorly, frontoparietals articulate with a well-ossified sphenethmoid. Its posterior margin does not reach midpoint of orbit and is ventrally contact with parasphenoid. Cultriform process of parasphenoid is narrow and tapered anteriorly; widest at its base, about 8.9% of maximum skull width. Parasphenoid alae are long but shorter than cultriform process (46.7% length of cultriform process) and articulate with dorsal tip of medial ramus of pterygoid. Neopalatines are thin and articulate with sphenethmoid dorsally and with medial face of maxillary bone ventrally forming anterior edge of orbit. Septomaxillae are small, horseshoe-shaped with their tips narrowly separated medially and articulate with prevomer posteriorly. Large prevomers are broadly separated from each other medially; the separation is greater rostrally. Prevomers bear dentigerous processes on their posterior margin, slightly anterior to palatines. Columella (or stapes) is thin, broader medially, and well ossified, it articulates with pterygoids distally and prootics medially. Nasals are thin, caudally expanded, medially separated from each other; and articulate with neopalatines through a posterolateral process.

Maxillary arch bears many small teeth, visible ventrally, on maxillae and premaxillae. Premaxillae are narrowly separated medially with their alary processes long, anteriorly oriented and completely separated from each other and from nasals. Each premaxilla articulates laterally with maxilla. Maxillae are posteriorly tapered and in contact with quadratojugals. The triradiate pterygoid bears a long, curved rostral ramus oriented anterolaterally towards maxilla. Caudal ramus of pterygoid is slightly longer and much wider than medial ramus; the former reaches quadratojugal at its medial face, while the latter reaches lateral edge of otoccipital and parasphenoid ala.

Quadratojugal is slender and articulates with ventral ramus of T-shaped squamosal posterodorsally. Otic ramus (posterior ramus) of squamosal is oriented posteromedially, flattened dorsoventrally, thicker towards its base and much longer than the zygomatic ramus (anterior ramus); the latter is flattened laterally and 48.5% of the length of the otic ramus. Mandible is narrow and edentate. Mentomeckelians are small, slightly broadened medially and laterally, and moderately separated from each other; they contact distally with the dentaries which are posteriorly acuminate. Angulosplenial is long, arcuate and expanded at its posterior end; it articulates broadly by its anterolateral face with anteromedial face of dentaries. The only well-ossified portions of hyoid apparatus are two posteromedial processes, which are broadly expanded anteriorly and slightly expanded posteriorly. Both posteromedial processes present an anteroventral inclination and are narrowly separated from one another at their anterior ends, the separation distance between them increases posteriorly.

Postcranium (Fig. 28). The specimen has eight presacral vertebrae, none of them imbricate. First presacral vertebra (cervical vertebra) is wider than posterior vertebrae and has no diapophyses. The cervical vertebra has a Type I cotylar arrangement. Foramen magnum is 20.7% the length of maximum skull width.

Presacral vertebrae II–VIII bear well-developed diapophyses. The transversal processes of presacral III are the longest and widest, and broadly expanded distally. In length, processes of presacral III are followed by presacrals IV and II, respectively. In width, processes of presacral III are followed by those of presacral II, slightly expanded distally, and presacral IV, not expanded distally. Transverse processes of presacrals V–VIII are the smallest and similar in size (length and width). The transversal processes of presacrals II and III have a ventral disposition in relation to the transverse processes of presacrals IV to VIII. Processes of presacrals V–VII are posteriorly oriented and those of presacral VII, laterally. Vertebra are characterized by having a stegochordal centrum which embraces a solidly ossified centrum, depressed in transverse section and with no notochordal remnants.

Sacrum bears moderately expanded diapophyses. Transverse processes are oriented dorsolaterally with an angle of dorsal opening of ~157 degrees and in contact distally with anterior tips of ilia. Sacrum is articulated caudally with urostyle by a bicondylar articulation. Urostyle is long, thin, and as long as the presacral portion of the vertebral column; it bears a well-developed keel broadly expanded anteriorly and gradually decreasing in height posteriorly. Urostyle lacks transverse processes. Sacrum-ilia articulation is not visible in micro CT-scan. Ilia articulate posteromedially with each other and posteriorly with ischia.

In the pectoral girdle, clavicles are long and slim, oriented anteromedially; right clavicle has straight edges (rostral and caudal), whereas rostral edge of left clavicle is anteriorly concave. Both clavicles are articulated medially with each other and with epicoracoids. Coracoids are robust, with curved anterior edges (the curvature is more pronounced distally), and straight posterior edge; their medial edges are moderately separated. Glenoidal and sternal ends of coracoid are about equally expanded. Scapula is long with anterior edge slightly oriented anteromedially. Epicoracoids are incomplete. Sternum is not well-ossified. Omosternum is absent.

Manus and pes (Fig. 29). All phalanges are well ossified with a phalangeal formula for fingers and toes: 2-2-3-3 and 2-2-3-4-3, respectively. Order of finger length is I < II < IV < III, and that of toes is I < II < III < V < IV. Terminal phalanges of all toes and fingers are narrower distally with a T-shaped tip, wider in fingers III and IV and in toes IV and V. Proximal end of distal toe phalanges bear small bone projections, more conspicuous in toes IV and V. It is difficult to distinguish the different elements of carpus and tarsus due to low resolution of three-dimensional models.

**Distribution, natural history, and conservation status (Fig. 1):** *Pristimantis tamia* is known from the surroundings of Zarentza Community (elevation range is 1,221–1,419 m.a.s.l.), Llanganates National Park. Ecosystem types are Eastern Montane Forest and Eastern Foothill Forest, as defined by Ron, Merino-Viteri & Ortiz (2021). All specimens were collected at night over vegetation, perching on leaves or branches, 50 to 500 cm above ground, in primary forests, sometimes nearby creeks.

Because of the lack of information on population size and geographic range, we assign *P. tamia* to the Data Deficient IUCN Red List Category (based on IUCN, 2019 guidelines). We note, however, that *P. tamia* was one of the most common anurans (74 individuals) during collections carried out at the type locality between 14 and 27 February 2015. The land use and vegetation cover map (obtained from Ministerio del Ambiente del Ecuador, 2018) shows an area deforested for agriculture and human settlements corresponding to 11.55% of the total area within 5 km of the known localities. The collection localities are at a distance of 8.6 km from the E45 highway and 12.1 km from Mera, a populated canton in the province of Pastaza.

**Etymology:** The specific epithet is a noun in apposition. Tamia is a Quichua noun which means rain and refers to the high precipitation characteristic of the type locality in Llanganates National Park. The name also refers to Tamia Lucía Ortega Páez, the older sister of the leading author in gratitude for all her love and support.

## Discussion

### A new clade of non-cryptic species

Surprisingly, our analyses show the existence of a new group of *Pristimantis* composed only of new species, the *P. anaiae* species group. This group is sister to the subgenus *Huicundomantis* and the large clade of small-sized *Pristimantis* reported by Zumel, Buckley & Ron (2021) which includes the *P. orestes*, *P. trachyblepharis*, and *P. chalceus* species groups (Figs. 5, S1- available at: https://doi.org/10.5281/zenodo.6484936). Most *Pristimantis* described during the last decade are morphologically cryptic species and were discovered with the crucial aid of genetic evidence (*e.g*., Páez & Ron, 2019; Zumel, Buckley & Ron, 2021; de Oliveira et al., 2020; Patiño-Ocampo, Duarte-Marin & Rivera-Correa, 2022). In contrast, the species of the *P. anaiae* species group are readily diagnosable using morphological characters (Fig. 5). So, what could explain that such a divergent and distinctive clade has remained hidden for so long?

Undoubtedly, the lack of collections in regions of difficult access that are protected has been important. Some areas of Sangay and Llanganates National Parks remain forested and unexplored as result of the lack of roads, extreme climatic conditions, and complex topography (Ministerio del Ambiente del Ecuador, 2017). The finding of these new species highlights the importance of protected areas to preserve montane ecosystems (Fiallo-Pantziou, 2010; Ríos Alvear & Reyes-Puig, 2015; Páez & Ron, 2019).

Another significant factor in the current discovery of new species seems to be the deployment of a large group of Ecuadorian amphibian taxonomists working on a hot spot of amphibian diversity (*e.g*., Ortega-Andrade et al., 2015; Navarrete, Venegas & Ron, 2016; Ramírez-Jaramillo et al., 2018; Sánchez-Nivicela et al., 2018; Szèkely et al., 2018; Páez & Ron, 2019, Reyes-Puig et al., 2019; Valencia et al., 2019; Yánez-Muñoz et al., 2019; Carrión-Olmedo & Ron, 2021; Zumel, Buckley & Ron, 2021; Guayasamin et al., 2022). This large group of taxonomists, working on several Ecuadorian institutions, would explain why Ecuador is the region with the highest density of new amphibian species described in the world between 2016 and 2020 (Womack et al., 2022).

### Species delimitation and morphology

Our phylogeny revealed the existence of nine new species from Sangay National Park and Llanganates National Park, six of them described in this manuscript (*i.e*., *P. anaiae, P. glendae, P. kunam, P. resistencia, P. tamia* and *P. venegasi*) (Figs. 4–6). The described species are clustered in two well-differentiated and strongly supported clades. Species limits were defined using genetic and morphological evidence which strongly indicates that each described species represents an independent, and previously unknown, evolutionary lineage. Genetic distances, based on the 16S gene, between *P. tamia* sp. nov. and its closest relatives are above 9.4% (Table 2); distances between the new species of the *P. anaiae* species group are above 4%. These distances surpass typical values observed between closely related species in *Pristimantis* (Padial & De la Riva, 2009; Funk, Caminer & Ron, 2012; Ortega-Andrade et al., 2015; Páez & Ron, 2019) and other Neotropical frogs (*e.g*., Fouquet et al., 2007; Coloma et al., 2012; Funk, Caminer & Ron, 2012; Jungfer et al., 2013; Caminer & Ron, 2014).

The morphological traits most useful for the diagnosis of the species described here are iris and dorsal coloration, skin texture, and condition of the vocal slits (present or absent). Additional useful characters are the condition of the tympanic annulus and the tympanic membrane. In *P. glendae, P. resistencia*, *and P. venegasi* both structures can be easily seen, but in *P. anaiae, P. kunam*, and *P. tamia*, only the tympanic annulus is present (Figs. 8, 10, 25 and 27). The presence of tympanic annulus and columella, which can be easily seen in CT-scans of *P. anaiae* and *P. tamia* (Figs. 8, 10, 25 and 27), without a tympanic membrane is congruent with the normal development of acoustic structures in the tympanic middle ear, where the development advances from the innermost structure (the columella) to the outermost one (the tympanic membrane), and never in the opposite direction (Helff, 1928; Barry, 1956; Sedra & Michael, 1959; Hetherington, 1987; Smirnov, 1991; Fabrezi & Goldberg, 2009; Pereyra et al., 2016). In our species, the absence of the membrane may be a result of a disruption in particular developmental stages (Pereyra et al., 2016) and may imply functional adaptations as the development of alternative communication channels based on different sensory systems (*e.g*., chemical, olfactory, visual stimuli) (Belanger & Corkum, 2009; von May, Lehr & Rabosky, 2018). This structural loss has been observed repeatedly within some Strabomantidae genera, mainly from high elevations (Hedges, Duellman & Heinicke, 2008; Duellman & Lehr, 2009; Padial, Grant & Frost, 2014); however, how these species communicate is still unknown (von May, Lehr & Rabosky, 2018).

Interestingly, in the CT-scans of *Pristimantis anaiae* and *P. tamia*, we found structures that appear to be intercalary elements. These structures are additional skeletal components with different degrees of mineralization, located between the penultimate and distal phalanges of hands as well as feet (Manzano, Fabrezi & Vences, 2007). In our X-ray based CT-scans, intercalary elements are visible in fingers III and IV and toes I, III, IV and V of *P. anaiae* and in all fingers and toes of *P. tamia* (Figs. 12, 29). Non-ossified intercalary elements cannot be detected by our CT-scans. Therefore, we cannot rule out their presence in fingers III and IV and Toe II of *P. anaiae*. So far, the existence of these elements has not been reported in species of Terrarana (Hedges, Duellman & Heinicke, 2008; Padial, Grant & Frost, 2014); however, the lack of previous records could be an artifact of lack of surveys for intercalary elements.

The function of intercalary elements in *Pristimantis* is unknown; however, the presence of intercalary elements in other Neobatrachia groups (*e.g*., Centrolenidae, Hylidae, Hemiphractidae, Microhylidae) (Lynch, 1971; Wiens et al., 2005; Frost et al., 2006; Manzano, Fabrezi & Vences, 2007) is associated with well-developed digit pads and arboreal habits (*e.g*., Paukstis & Brown, 1987; Paukstis & Brown, 1991; Wiens et al., 2005). Intercalary elements provide two additional joints that enable a wider range of finger movements without the need to detach digits pads from the surface, a property that enhances the ability to climb (Hanna & Barnes, 1991). Intercalary elements are family-level characteristics, suggesting that they are of great evolutionary significance (Manzano, Fabrezi & Vences, 2007). Further studies are still needed to confirm that the observed structures are intercalary elements. Their implications on ecological, evolutionary, and behavioral aspects of *Pristimantis* also need to be studied. These elements could be associated with the arboreal habits of many species of *Pristimantis*; for now, this relationship is an hypotheses that needs to be tested. If correct, this finding could shed light on the massive diversification that this genus has undergone.

### Phylogenetic position of *Pristimantis bicantus*

Our results indicate that *P. bicantus* is not part of the *P. myersi* species group, where was placed by Guayasamin & Funk (2009). Instead, it is closely related to *P. nelsongalloi* and *P. sacharuna* which have not been assigned to a species group until now (Reyes-Puig et al., 2015; Valencia et al., 2019). In our study, *P. bicantus*, *P. nelsongalloi*, and *P. sacharuna* are sister to two undescribed species from Llanganates (*e.g*., QCAZ 69791 and 70020) (Fig. 6). This five species clade is sister to *P. prolatus* (Fig. 6). All these species inhabit montane forest in the eastern versant of the Andes of Ecuador.

## Conclusions

We provide morphological and genetic evidence that validate the description of six new species, *P. tamia* sp. nov., *P. anaiae* sp. nov., *P. glendae* sp. nov., *P. kunam* sp. nov., *P. resistencia* sp. nov., and *P. venegasi* sp. nov. We include a well-supported phylogeny and morphological descriptions that allow us to propose to new species groups as well as an expansion of the subgenus *Huicundomantis*.

The *P. anaiae* species group is remarkable because it forms a highly divergent clade and is formed exclusively by species new science.

## Supplemental Information

10.7717/peerj.13761/supp-1 Supplemental Information 1 Outgroup individuals included in the phylogenetic analyses.

10.7717/peerj.13761/supp-2 Supplemental Information 2 Snout-Vent length (SVL) and sex of the individuals of *the Pristimantis anaiae* species group..

10.7717/peerj.13761/supp-3 Supplemental Information 3 Snout-Vent length (SVL) and sex of the individuals of *Pristimantis tamia* sp. nov.

10.7717/peerj.13761/supp-4 Supplemental Information 4 Other examined specimens.

10.7717/peerj.13761/supp-5 Supplemental Information 5 Best partition scheme and evolution models for each phylogenetical analysis performed.

10.7717/peerj.13761/supp-6 Supplemental Information 6 Character loading and proportion of the variance explained by Principal Components for morphometric variables for *P. tamia* and its closest relatives. For each component, variables with the highest loadings are shown in bold. Size was removed during procrustes, along with rotation and translational placement. The landmarks that provide the greatest variation in the first and second components are related to the ocular cavity, the otoccipital crests and the mandibular joint.

10.7717/peerj.13761/supp-7 Supplemental Information 7 Phylogenetic relationships, based on nuclear genes only, showing *Pristimantis* new species relations. Maximum likelihood tree obtained for gene RAG1. Support values are on the corresponding branches: aLRT values above the slash and bootstrap below; missing values indicate values below 50 (aLRT and bootstrap). The phylogeny was derived from an analysis of 582 bp for 103 samples of nuclear (gene fragments RAG1) DNA sequences. For each specimen, museum number or, in unavailable, GenBank accession number is shown, as well as its locality. Outgroup is not shown. Abbreviations: CCS = confirmed candidate species, UCS = unconfirmed candidate species, ECU = Ecuador. In red, the inconsistencies found.

## Additional Information and Declarations

### DNA Deposition

The following information was supplied regarding the deposition of DNA sequences: The sequences are available at in GenBank (Table 1).

### Data Availability

The following information was supplied regarding data availability: The complete DNA sequence matrix is available at Zenodo: Ortega Jhael, Brito, Jorge, & Ron, Santiago. (2022). Six new species of Pristimantis (Anura: Strabomantidae) from Llanganates National Park and Sangay National Park [Data set]. Zeonodo. http://doi.org/10.5281/zenodo.7055289. The CT data are available at Zenodo: Ortega, Jhael, Brito, Jorge, & Santiago, Ron. (2022). Six new species of Pristimantis (Anura: Strabomantidae) from Llanganates National Park and Sangay National Park in Amazonian cloud forests of Ecuador. Zenodo. https://doi.org/10.5281/zenodo.6323699.

### New Species Registration

The following information was supplied regarding the registration of a newly described species: Publication LSID: urn:lsid:zoobank.org:pub:AFBD6AC9-D3E2-4112-AC3F-A2156268ECB3 Pristimantis anaiae: urn:lsid:zoobank.org:act:020CF5BA-B737-4F45-B9B9-209A9F1AE223 Pristimantis glendae: urn:lsid:zoobank.org:act:D4D4D052-6015-4B02-8C9F-E336020922AA Pristimantis kunam: urn:lsid:zoobank.org:act:7DD2C62D-E728-42CD-BDD5-F526EDD8D4FD Pristimantis resistencia: urn:lsid:zoobank.org:act:9A710AB8-CE6C-4FC7-A0AC-7A516ACABC92 Pristimantis venegasi: urn:lsid:zoobank.org:act:6E60CD7B-B045-42E0-9C3D-4DFEE556ACE7 Pristimantis tamia: urn:lsid:zoobank.org:act:458A8123-E7B4-4F10-BB0C-0CB97BFD67D1
