# Supplementary material for: Six new species of Pristimantis (Anura: Strabomantidae) from Llanganates National Park and Sangay National Park in Amazonian cloud forests of Ecuador
Source: PeerJ. 2022 Oct 17;10:e13761. doi: 10.7717/peerj.13761 (PMC9583859; doi:10.7717/peerj.13761)
Supplement: Supplemental Information 1 [file peerj-10-13761-s001.docx]

| **Species** | **Museum number** | **GenBank number** | **Locality** |
| --- | --- | --- | --- |
| *Lynchius oblitus* | QCAZ61035 | MZ241534 | Loja. Ecuador |
| *P.* sp. | QCAZ26586 | MT372721 | Carchi. Ecuador |
| *Pristimantis euphronides* | BWMC6918 | EF493527 | Grand Etang. Grenada |
| *Pristimantis labiosus* | QCAZ19771 | EF493694 | Pichincha. Ecuador |
| *Pristimantis latidiscus* | QCAZ17101 | EF493354 | Esmeraldas. Ecuador |
| *Pristimantis rubicundus* | QCAZ26551 | MT372710 | Morona Santiago. Ecuador |
| *Pristimantis shrevei* |  | EF493692 | Vermont. USA |
