# Supplementary material for: Six new species of Pristimantis (Anura: Strabomantidae) from Llanganates National Park and Sangay National Park in Amazonian cloud forests of Ecuador
Source: PeerJ. 2022 Oct 17;10:e13761. doi: 10.7717/peerj.13761 (PMC9583859; doi:10.7717/peerj.13761)
Supplement: Supplemental Information 2 [file peerj-10-13761-s002.docx]

| **Species** | **QCAZ** | **SEX** | **SVL (mm)** | **Sequenced** |
| --- | --- | --- | --- | --- |
| *P. anaiae* | 59566 | Male | 17.02 | Yes |
| *P. anaiae* | 59597 | Male | 18.99 | No |
| *P. anaiae* | 59627 | Male | 18.73 | Yes |
| *P. anaiae* | 59640 | Male | 20.63 | Yes |
| *P. anaiae* | 59655 | Male | 18.10 | No |
| *P. anaiae* | 59658 | Male | 17.37 | No |
| *P. anaiae* | 59693 | Male | 18.07 | Yes |
| *P. anaiae* | 59720 | Male | 13.08 | Yes |
| *P. glendae* | 45739 | Male | 19.93 | Yes |
| *P. glendae* | 45784 | Male | 19.09 | Yes |
| *P. glendae* | 45832 | Male | 19.70 | Yes |
| *P. glendae* | 45953 | Male | 18.29 | Yes |
| *P. glendae* | 56437 | Male | 16.60 | Yes |
| *P. kunam* | 56438 | Male | 14.66 | Yes |
| *P. resistencia* | 66372 | Male | 17.56 | No |
| *P. resistencia* | 66467 | Male | 19.75 | Yes |
| *P. resistencia* | 66519 | Female | 24.59 | Yes |
| *P. resistencia* | 66523 | Male | 19.40 | Yes |
| *P. venegasi* | 31130 | Male | 24.49 | Yes |
| *P. venegasi* | 66440 | Female | 34.90 | Yes |
