# Supplementary material for: Six new species of Pristimantis (Anura: Strabomantidae) from Llanganates National Park and Sangay National Park in Amazonian cloud forests of Ecuador
Source: PeerJ. 2022 Oct 17;10:e13761. doi: 10.7717/peerj.13761 (PMC9583859; doi:10.7717/peerj.13761)
Supplement: Supplemental Information 3 [file peerj-10-13761-s003.docx]

**Table S3. Snout-Vent length (SVL) and sex of the individuals of Pristimantis tamia sp. nov.**

| **QCAZ** | **Sex** | **SVL (mm)** | **Sequenced** |
| --- | --- | --- | --- |
| 59439 | Female | 26.78 | Yes |
| 59445 | Male | 17.45 | Yes |
| 59564 | Female | 25.65 | Yes |
| 59565 | Male | 18.43 | Yes |
| 59568 | Male | 17.68 | No |
| 59570 | Male | 15.42 | No |
| 59573 | Female | 25.41 | Yes |
| 59577 | Male | 17.13 | No |
| 59581 | Female | 26.75 | Yes |
| 59582 | Female | 25.78 | Yes |
| 59584 | Female | 26.59 | Yes |
| 59585 | Male | 18.09 | Yes |
| 59590 | Male | 23.30 | No |
| 59591 | Male | 19.49 | No |
| 59619 | Male | 19.81 | Yes |
| 59620 | Male | 19.26 | Yes |
| 59629 | Female | 22.23 | Yes |
| 59630 | Female | 26.53 | Yes |
| 59631 | Male | 19.76 | No |
| 59633 | Male | 15.87 | No |
| 59635 | Male | 17.85 | Yes |
| 59636 | Female | 26.89 | Yes |
| 59642 | Female | 23.50 | Yes |
| 59643 | Female | 26.58 | Yes |
| 59644 | Female | 27.81 | Yes |
| 59645 | Female | 27.31 | No |
| 59650 | Female | 22.19 | Yes |
| 59651 | Male | 19.08 | Yes |
| 59653 | Male | 16.12 | Yes |
| 59656 | Female | 26.95 | Yes |
| 59657 | Male | 24.82 | No |
| 59660 | Male | 19.32 | Yes |
| 59661 | Male | 20.54 | No |
| 59664 | Male | 18.52 | Yes |
| 59666 | Female | 25.59 | Yes |
| 59668 | Female | 21.27 | Yes |
| 59672 | Male | 17.95 | Yes |
| 59673 | Male | 17.31 | No |
| 59675 | Male | 16.04 | Yes |
| 59676 | Male | 17.77 | No |
| 59680 | Female | 28.31 | Yes |
| 59695 | Female | 26.06 | No |
| 59696 | Male | 16.97 | Yes |
| 59701 | Female | 29.88 | Yes |
| 59702 | Male | 19.13 | Yes |
| 59704 | Female | 26.77 | Yes |
| 59710 | Female | 25.42 | Yes |
| 59713 | Male | 18.85 | Yes |
| 59719 | Female | 28.37 | Yes |
