# Supplementary material for: Six new species of Pristimantis (Anura: Strabomantidae) from Llanganates National Park and Sangay National Park in Amazonian cloud forests of Ecuador
Source: PeerJ. 2022 Oct 17;10:e13761. doi: 10.7717/peerj.13761 (PMC9583859; doi:10.7717/peerj.13761)
Supplement: Supplemental Information 4 [file peerj-10-13761-s004.docx]

| **Species** | **QCAZ** | **Sex** | **SVL mm** | **Locality** |
| --- | --- | --- | --- | --- |
| *Pristimantis miktos* | 10131 | Male | 18.72 | Pastaza. Lorocachi |
| *Pristimantis miktos* | 54920 |  | 17.11 | Orellana. Loreto. PNY |
| *Pristimantis miktos* | 56040 |  | 13.31 | Pastaza. Lorocachi |
| *Pristimantis miktos* | 64000 | Male | 19.18 | Orellana. PNY |
| *Pristimantis miktos* | 64001 | Male | 19.27 | Orellana. PNY |
