# Supplementary material for: Six new species of Pristimantis (Anura: Strabomantidae) from Llanganates National Park and Sangay National Park in Amazonian cloud forests of Ecuador
Source: PeerJ. 2022 Oct 17;10:e13761. doi: 10.7717/peerj.13761 (PMC9583859; doi:10.7717/peerj.13761)
Supplement: Supplemental Information 5 [file peerj-10-13761-s005.docx]

| **Analysis** | **Partition** | **Model** |
| --- | --- | --- |
| Concatened genes | 16S | TIM2+F+I+G4 |
| Concatened genes | 12S | TIM2+F+I+G4 |
| Concatened genes | ND1 1^st^ position | TPM2+F+G4 |
| Concatened genes | ND1 2 ^nd^ position | HKY+F+R2), |
| Concatened genes | ND1 3 ^rd^ position | TN+F+I+G4 |
| Concatened genes | RAG1 1 ^st^ position | JC+R2 |
| Concatened genes | RAG1 2 ^nd^ position | TN+F+G4 |
| Concatened genes | RAG1 3 ^rd^ position | K2P+R2 |
| Concatened genes | NonCoding segment | TIM2e+G4 |
| Nuclear gene | RAG1 1 ^st^ position | JC+G4 |
| Nuclear gene | RAG1 2 ^nd^ position | TN+F+G4 |
| Nuclear gene | RAG1 3 ^rd^ position | K2P+G4 |
| Mitochondrial genes | 16S | TIM2+R6 |
| Mitochondrial genes | 12S | GTR-R5 |
| Mitochondrial genes | ND1 1^st^ position | TPM2+G4 |
| Mitochondrial genes | ND1 2 ^nd^ position | HKY+I+G4 |
| Mitochondrial genes | ND1 3 ^rd^ position | TN+F+I+G4 |
| Mitochondrial genes | NonCoding segment | TIM2e |
