# Supplementary material for: Six new species of Pristimantis (Anura: Strabomantidae) from Llanganates National Park and Sangay National Park in Amazonian cloud forests of Ecuador
Source: PeerJ. 2022 Oct 17;10:e13761. doi: 10.7717/peerj.13761 (PMC9583859; doi:10.7717/peerj.13761)
Supplement: Supplemental Information 6 — For each component, variables with the highest loadings are shown in bold. Size was removed during procrustes, along with rotation and translational placement. The landmarks that provide the greatest variation in the first and second components are related to the ocular cavity, the otoccipital crests and the mandibular joint. [file peerj-10-13761-s006.docx]

|  | **PC1** | **PC2** | **PC3** | **PC4** | **PC5** | **PC6** | **PC7** | **PC8** | **PC9** |
| --- | --- | --- | --- | --- | --- | --- | --- | --- | --- |
| **1.X** | 0.0289 | 0.0843 | -0.1606 | 0.0079 | 0.1092 | 0.1165 | 0.0182 | 0.0692 | -0.2323 |
| **1.Y** | -0.0243 | -0.0020 | 0.0867 | 0.0408 | -0.1156 | 0.1050 | 0.0458 | 0.0146 | -0.0925 |
| **1.Z** | 0.0073 | 0.0546 | 0.0356 | -0.0610 | 0.1239 | 0.1399 | 0.1120 | -0.0038 | 0.0105 |
| **2.X** | -0.0511 | 0.1076 | -0.1861 | -0.0103 | 0.1586 | 0.1324 | -0.0758 | 0.0017 | -0.0169 |
| **2.Y** | 0.0139 | 0.0235 | 0.0829 | 0.0379 | **0.2001** | -0.0908 | 0.1423 | -0.0351 | -0.1545 |
| **2.Z** | 0.0399 | 0.0614 | 0.0741 | -0.0155 | 0.0212 | -0.1203 | 0.0656 | 0.1005 | -0.0976 |
| **3.X** | -0.0447 | 0.0936 | -0.1604 | -0.1407 | 0.0706 | 0.0869 | 0.1060 | 0.1593 | -0.0258 |
| **3.Y** | 0.0082 | -0.0486 | -0.1246 | -0.0562 | -0.0645 | 0.1569 | -0.0273 | -0.1432 | 0.0358 |
| **3.Z** | 0.0420 | 0.0831 | 0.0791 | 0.0615 | 0.0948 | -0.1226 | -0.0645 | 0.0600 | -0.0710 |
| **4.X** | 0.1076 | -0.0563 | -0.1679 | -0.0638 | -0.2506 | -0.1390 | 0.0821 | -0.0655 | 0.0288 |
| **4.Y** | -0.1141 | -0.0049 | -0.0378 | 0.0649 | 0.0967 | -0.0574 | 0.0943 | 0.0774 | 0.0458 |
| **4.Z** | 0.0968 | 0.1123 | 0.0413 | -0.0296 | -0.0360 | 0.0432 | -0.0875 | -0.1463 | 0.0510 |
| **5.X** | 0.0858 | -0.0130 | -0.1036 | -0.0143 | -0.0819 | -0.0685 | 0.1592 | **0.2095** | **0.2050** |
| **5.Y** | 0.1388 | -0.0760 | -0.0014 | -0.1168 | -0.1563 | 0.0142 | 0.0107 | -0.0046 | 0.1715 |
| **5.Z** | 0.1031 | 0.0611 | 0.0504 | -0.1217 | 0.0745 | -0.0551 | -0.0900 | 0.0618 | 0.0062 |
| **6.X** | 0.1307 | -0.0403 | 0.1820 | -0.2390 | 0.0970 | -0.1105 | 0.1630 | -0.0456 | -0.1266 |
| **6.Y** | 0.0338 | -0.0428 | 0.0934 | 0.0214 | 0.0371 | -0.0376 | -0.2680 | 0.1109 | -0.0853 |
| **6.Z** | 0.0217 | 0.1558 | 0.1574 | -0.0650 | 0.0109 | -0.1048 | -0.0546 | 0.0400 | -0.0165 |
| **7.X** | 0.1956 | 0.0169 | 0.2109 | -0.2307 | -0.0142 | -0.0462 | 0.1646 | -0.0077 | -0.0087 |
| **7.Y** | -0.1748 | -0.0813 | -0.1486 | -0.0626 | 0.1006 | -0.1033 | **0.2189** | -0.0337 | 0.0866 |
| **7.Z** | -0.0410 | 0.1379 | 0.1215 | -0.0753 | 0.0149 | -0.1486 | 0.0045 | 0.0092 | 0.0448 |
| **8.X** | -0.0599 | -0.0764 | -0.0392 | -0.0793 | 0.0722 | 0.1764 | -0.0073 | 0.1471 | -0.0319 |
| **8.Y** | -0.0881 | 0.0054 | 0.1439 | 0.0494 | -0.0496 | -0.0111 | -0.0906 | 0.0647 | 0.1236 |
| **8.Z** | -0.0404 | 0.0158 | -0.0854 | 0.0037 | 0.0173 | -0.0492 | 0.0412 | 0.0378 | 0.1455 |
| **9.X** | -0.0827 | 0.0229 | 0.1059 | -0.1601 | 0.0482 | 0.1817 | -0.1910 | -0.0970 | -0.0601 |
| **9.Y** | 0.0828 | -0.0512 | -0.1238 | 0.0437 | 0.0051 | -0.0509 | **0.2272** | -0.0917 | -0.1356 |
| **9.Z** | -0.0368 | -0.0598 | -0.0154 | -0.0406 | 0.0406 | -0.1094 | 0.0596 | 0.0369 | 0.0623 |
| **10.X** | -0.0433 | 0.0998 | -0.0265 | 0.0334 | -0.0685 | -0.0895 | -0.1842 | 0.1089 | 0.0371 |
| **10.Y** | 0.0510 | -0.2730 | 0.0600 | 0.0174 | -0.1334 | -0.1200 | 0.0611 | 0.1481 | -0.1520 |
| **10.Z** | -0.0085 | 0.0227 | 0.0376 | 0.0300 | 0.1226 | 0.0183 | 0.0323 | -0.1790 | 0.0364 |
| **11.X** | -0.1274 | 0.0658 | -0.0154 | **0.2230** | -0.2688 | 0.0705 | -0.1496 | 0.0378 | 0.0805 |
| **11.Y** | -0.0383 | **0.2988** | -0.1364 | -0.0206 | -0.0056 | 0.0812 | 0.0522 | 0.0111 | -0.0826 |
| **11.Z** | -0.0909 | 0.0986 | 0.0282 | **0.3681** | -0.1820 | **0.2126** | 0.1129 | 0.0793 | -0.1821 |
| **12.X** | 0.0344 | 0.0059 | 0.0063 | -0.0505 | 0.0042 | 0.0289 | -0.0042 | -0.1110 | 0.1361 |
| **12.Y** | 0.0675 | 0.0003 | -0.0355 | -0.0926 | 0.0502 | 0.0148 | -0.0464 | 0.0434 | -0.0380 |
| **12.Z** | -0.0463 | -0.0869 | -0.0472 | 0.0447 | 0.0548 | 0.0598 | 0.0269 | -0.1359 | 0.0883 |
| **13.X** | 0.0449 | 0.0474 | -0.0862 | 0.0269 | 0.0819 | -0.0458 | -0.0714 | 0.0154 | 0.0567 |
| **13.Y** | -0.0228 | -0.0147 | -0.0208 | 0.1475 | -0.0489 | -0.1516 | -0.0152 | -0.1048 | 0.0149 |
| **13.Z** | -0.0954 | -0.1063 | -0.1295 | 0.1119 | -0.1603 | 0.0293 | -0.0080 | -0.1945 | 0.0284 |
| **14.X** | -0.1587 | -0.0391 | 0.0456 | -0.0318 | -0.1498 | -0.0182 | -0.0971 | -0.0717 | -0.0815 |
| **14.Y** | -0.1646 | 0.0255 | 0.0124 | 0.0772 | -0.1363 | -0.0915 | -0.0134 | 0.0188 | 0.0117 |
| **14.Z** | -0.0043 | -0.1310 | -0.1567 | -0.2221 | -0.1119 | -0.0740 | 0.0102 | -0.2841 | 0.0121 |
| **15.X** | -0.2090 | 0.0335 | 0.1464 | -0.0173 | -0.1280 | 0.0455 | 0.0928 | -0.0329 | 0.0216 |
| **15.Y** | 0.1041 | -0.0867 | -0.1182 | -0.0118 | 0.1174 | -0.0902 | -0.0725 | -0.1119 | -0.0572 |
| **15.Z** | -0.0271 | -0.0630 | -0.0461 | **0.2790** | 0.1413 | -0.0419 | 0.1148 | -0.1005 | -0.0869 |
| **16.X** | -0.0561 | -0.1966 | -0.0204 | 0.0020 | -0.1054 | -0.1522 | -0.0290 | 0.0101 | -0.1462 |
| **16.Y** | -0.0650 | **0.2767** | 0.1035 | 0.0699 | 0.1328 | 0.1701 | 0.0960 | 0.0036 | -0.0117 |
| **16.Z** | 0.0838 | -0.1001 | -0.0540 | 0.0363 | -0.0748 | 0.0438 | -0.0358 | 0.1766 | -0.0114 |
| **17.X** | -0.0412 | -0.0527 | 0.0290 | -0.0205 | -0.0147 | 0.0187 | 0.0392 | 0.0458 | -0.1446 |
| **17.Y** | 0.0344 | -0.0547 | 0.0067 | -0.1075 | -0.0212 | 0.0509 | 0.0723 | 0.1571 | -0.1575 |
| **17.Z** | 0.1211 | -0.0614 | 0.0100 | 0.0359 | -0.0572 | 0.0986 | -0.1093 | 0.0684 | 0.0078 |
| **18.X** | -0.0003 | 0.0772 | -0.0376 | -0.0980 | -0.0254 | -0.1840 | -0.0623 | 0.0892 | 0.0636 |
| **18.Y** | -0.0850 | 0.0650 | 0.0505 | -0.2106 | -0.0118 | -0.0774 | 0.1135 | -0.0853 | 0.1753 |
| **18.Z** | 0.0092 | 0.0021 | -0.0415 | 0.0364 | 0.1559 | 0.0305 | -0.0824 | 0.0639 | **0.2640** |
| **19.X** | -0.0976 | 0.0386 | -0.1300 | -0.0564 | -0.3055 | 0.0232 | -0.0363 | -0.1035 | 0.0254 |
| **19.Y** | 0.2087 | -0.1717 | 0.0855 | -0.0877 | 0.0108 | 0.0950 | -0.1911 | -0.1015 | 0.1248 |
| **19.Z** | 0.0430 | -0.0241 | -0.0971 | 0.0453 | **0.1848** | -0.0157 | -0.0155 | 0.0438 | 0.1846 |
| **20.X** | -0.0281 | 0.0463 | 0.0029 | 0.0841 | 0.0582 | -0.0234 | 0.0960 | 0.0755 | 0.0543 |
| **20.Y** | -0.1264 | 0.1063 | 0.1712 | 0.0374 | 0.0338 | 0.0992 | -0.2242 | -0.2898 | 0.0344 |
| **20.Z** | 0.0522 | -0.0847 | -0.0245 | 0.1597 | -0.0797 | -0.0232 | 0.0610 | 0.1005 | 0.0297 |
| **21.X** | -0.0832 | -0.0333 | -0.0718 | 0.0508 | 0.1063 | -0.0818 | 0.0281 | -0.0534 | -0.1050 |
| **21.Y** | 0.2062 | 0.0884 | -0.0459 | 0.0752 | -0.2206 | -0.1416 | -0.0512 | 0.1436 | 0.1465 |
| **21.Z** | 0.0822 | 0.0042 | 0.0243 | 0.0039 | 0.0099 | -0.1687 | -0.0582 | -0.1317 | -0.0910 |
| **22.X** | 0.0361 | -0.2010 | -0.0845 | 0.0224 | 0.1145 | 0.0045 | -0.0912 | -0.0705 | -0.0824 |
| **22.Y** | 0.1108 | -0.0733 | -0.0553 | 0.0763 | 0.0515 | 0.0852 | -0.0034 | 0.0376 | 0.0736 |
| **22.Z** | -0.0882 | 0.0121 | 0.1206 | -0.1122 | -0.1095 | 0.0805 | -0.0559 | -0.0166 | -0.2931 |
| **23.X** | 0.1164 | -0.1295 | -0.1125 | 0.0260 | **0.1711** | 0.1861 | 0.0114 | -0.0290 | -0.0571 |
| **23.Y** | -0.1440 | 0.0317 | 0.0212 | -0.0229 | -0.0148 | -0.0775 | -0.0274 | 0.0153 | 0.0879 |
| **23.Z** | -0.1380 | -0.0157 | -0.0428 | -0.2592 | -0.0005 | -0.0340 | -0.2551 | 0.1235 | -0.1282 |
| **24.X** | -0.0465 | 0.0770 | 0.0439 | 0.1752 | 0.0577 | -0.2681 | 0.0260 | -0.1836 | -0.0227 |
| **24.Y** | -0.2939 | 0.0324 | -0.0034 | -0.0565 | 0.1224 | -0.0372 | -0.0331 | 0.0836 | 0.0202 |
| **24.Z** | 0.0234 | 0.1968 | **0.2371** | -0.0875 | -0.1403 | -0.0224 | 0.1122 | 0.0094 | 0.0485 |
| **25.X** | -0.1139 | 0.1249 | -0.0403 | 0.1554 | 0.1448 | -0.2330 | -0.1235 | -0.0597 | 0.0299 |
| **25.Y** | **0.2960** | -0.0062 | -0.1114 | 0.1675 | 0.0251 | 0.0105 | -0.1977 | 0.0144 | -0.1211 |
| **25.Z** | 0.0316 | -0.0157 | 0.0186 | 0.0010 | -0.1159 | 0.0149 | **0.2832** | -0.0486 | -0.1269 |
| **26.X** | -0.0756 | -0.3373 | **0.3899** | 0.1237 | 0.0436 | **0.2216** | 0.1146 | -0.1087 | 0.1255 |
| **26.Y** | -0.0312 | -0.0546 | -0.0435 | -0.0131 | 0.0798 | -0.0180 | -0.0091 | -0.1670 | -0.1404 |
| **26.Z** | -0.0260 | -0.0843 | -0.0010 | -0.0584 | -0.0835 | 0.1660 | -0.0732 | 0.0700 | -0.0773 |
| **27.X** | -0.0089 | -0.2047 | **0.2513** | 0.1458 | 0.1450 | -0.0334 | 0.0043 | **0.2325** | **0.2296** |
| **27.Y** | 0.0140 | -0.0019 | 0.1183 | 0.0178 | -0.0080 | 0.0008 | -0.0688 | **0.2563** | -0.1663 |
| **27.Z** | 0.0898 | -0.0669 | 0.0154 | 0.0086 | 0.0494 | 0.0883 | -0.0666 | -0.0468 | 0.0734 |
| **28.X** | 0.2456 | 0.1387 | 0.0431 | 0.0686 | -0.0525 | 0.0526 | -0.1087 | -0.1124 | 0.0279 |
| **28.Y** | **0.0837** | 0.0341 | 0.0345 | 0.0645 | -0.0877 | 0.0640 | 0.1713 | -0.0659 | 0.0886 |
| **28.Z** | -0.0423 | -0.0591 | -0.0741 | 0.0085 | 0.0535 | -0.0933 | -0.0530 | 0.0736 | -0.0208 |
| **29.X** | **0.3022** | **0.3000** | -0.0140 | 0.0675 | -0.0181 | 0.1480 | 0.1260 | -0.0498 | 0.0196 |
| **29.Y** | -0.0815 | 0.0554 | -0.0644 | -0.1500 | 0.0110 | **0.2085** | 0.0338 | 0.0339 | 0.1535 |
| **29.Z** | -0.1620 | -0.0596 | -0.2359 | -0.0868 | -0.0187 | 0.1576 | 0.0732 | 0.1330 | 0.1096 |
