# Supplementary figures and images for: Six new species of Pristimantis (Anura: Strabomantidae) from Llanganates National Park and Sangay National Park in Amazonian cloud forests of Ecuador
Source: PeerJ. 2022 Oct 17;10:e13761. doi: 10.7717/peerj.13761 (PMC9583859; doi:10.7717/peerj.13761)

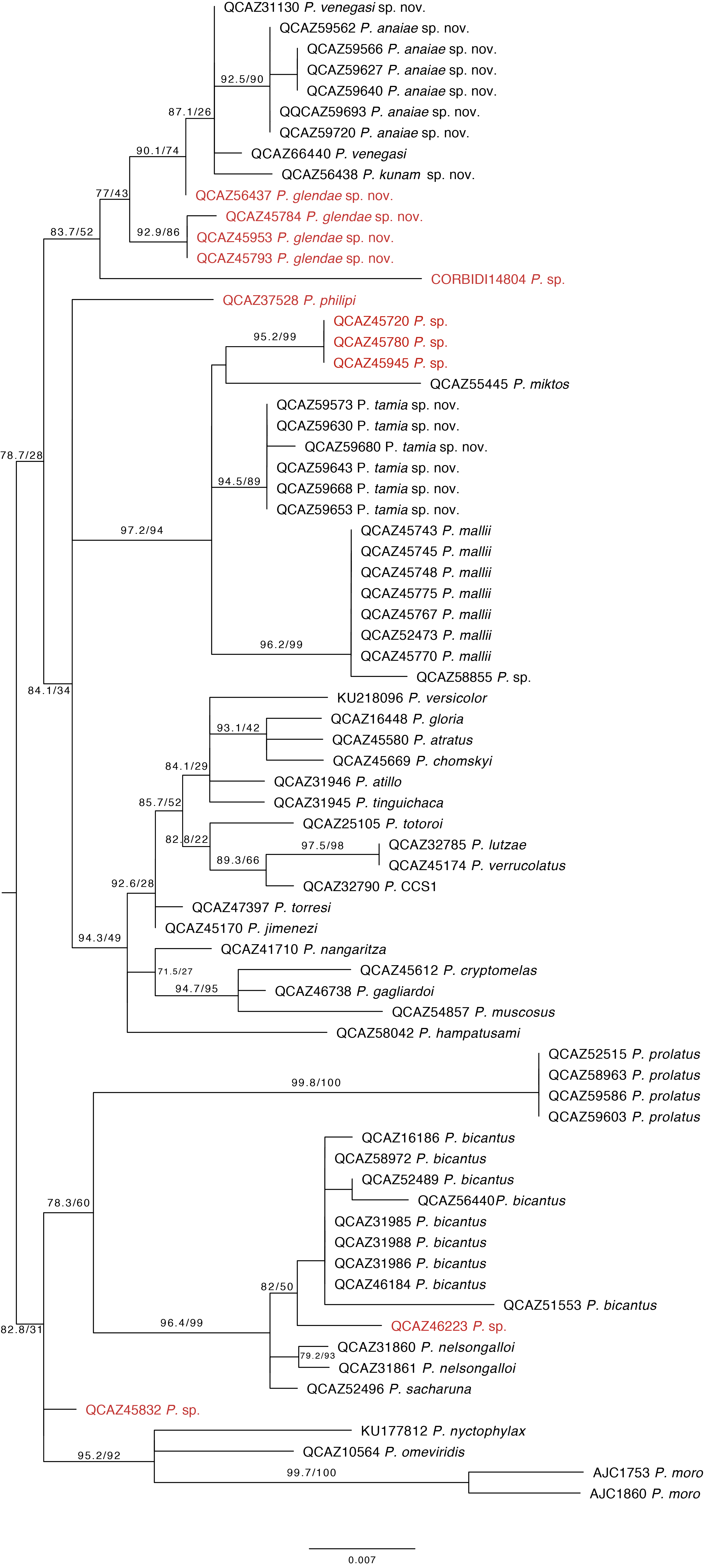

Supplement: Supplemental Information 7 — Maximum likelihood tree obtained for gene RAG1. Support values are on the corresponding branches: aLRT values above the slash and bootstrap below; missing values indicate values below 50 (aLRT and bootstrap). The phylogeny was derived from an analysis of 582 bp for 103 samples of nuclear (gene fragments RAG1) DNA sequences. For each specimen, museum number or, in unavailable, GenBank accession number is shown, as well as its locality. Outgroup is not shown. Abbreviations: CCS = confirmed candidate species, UCS = unconfirmed candidate species, ECU = Ecuador. In red, the inconsistencies found. [file peerj-10-13761-s007.png]
